# Pharmacological interventions on smoking cessation: A systematic review and network meta-analysis

**DOI:** 10.3389/fphar.2022.1012433

**Published:** 2022-10-24

**Authors:** Xue Shang, Kangle Guo, Fenfen E., Xinxin Deng, Yongsheng Wang, Ziyi Wang, Yanan Wu, Meng Xu, Chaoqun Yang, Xiuxia Li, Kehu Yang

**Affiliations:** ^1^ Health Technology Assessment Center/Evidence-Based Social Science Research Center, School of Public Health, Lanzhou University, Lanzhou, China; ^2^ Evidence Based Medicine Center, School of Basic Medical Sciences, Lanzhou University, Lanzhou, China; ^3^ Key Laboratory of Evidence Based Medicine and Knowledge Translation of Gansu Province, Lanzhou, China; ^4^ Gansu Provincial Hospital, Lanzhou, China

**Keywords:** pharmacotherapy, smoking cessation, Systematic review, network meta-analysis, tobacco

## Abstract

**Objective:** A network meta-analysis based on randomized controlled trials was conducted to investigate the effects of pharmacological interventions on smoking cessation.

**Methods:** English databases were searched to obtain randomized controlled trials reporting the effect of pharmacological interventions on smoking cessation. The risk of bias for the included trials was assessed using Cochrane Handbook tool. Stata 15.1 software was used to perform network meta-analysis, and GRADE approach was used to assess the evidence credibility on the effects of different interventions on smoking cessation.

**Results:** A total of 159 studies involving 60,285 smokers were included in the network meta-analysis. The analysis involved 15 interventions and which yielded 105 pairs of comparisons. Network meta-analysis showed that varenicline was more helpful for smoking cessation than other monotherapies, such as nicotine replacement therapy [Odds Ratio (OR) = 1.42, 95% confidence interval (CI) (1.16, 1.73)] and bupropion [OR = 1.52, 95% CI (1.22, 1.89)]. Furthermore, combined interventions were superior to monotherapy in achieving smoking cessation, such as varenicline plus bupropion over bupropion [OR = 2.00, 95% CI (1.11, 3.61)], varenicline plus nicotine replacement therapy over nicotine replacement therapy [OR = 1.84, 95% CI (1.07, 3.18)], and nicotine replacement therapy plus mecamylamine over naltrexone [OR = 6.29, 95% CI (1.59, 24.90)]. Finally, the surface under the cumulative ranking curve value indicated that nicotine replacement therapy plus mecamylamine had the greatest probability of becoming the best intervention.

**Conclusion:** Most pharmacological interventions demonstrated a benefit in smoking cessation compared with placebo, whether monotherapy or combination therapy. Moreover, confirmed evidence suggested that some combination treatments, such as varenicline plus bupropion and nicotine replacement therapy plus mecamylamine have a higher probability of being the best smoking cessation in

## Introduction

The tobacco epidemic remains a medical, social, economic, and public health problem. As of 2019, there were about 847 million male smokers and 157 million female smokers worldwide, according to the World Health Organization’s 2021 report (Burki, 2021). Tobacco use remains a major contributor to the global burden of disease, responsible for an estimated 12% of deaths among 30-year-olds worldwide, killing 8 million people globally each year, including 1.2 million non-smokers exposed to second-hand smoke (GBD 2017 Risk Factor Collaborators, 2017; Bernabe-Ortiz and Carrillo-Larco, 2021). Deaths from tobacco use are mainly caused by some smoking-related diseases, including malignant tumors (such as lung cancer), cardiovascular diseases (such as heart disease), respiratory diseases (such as chronic obstructive pulmonary disease), tuberculosis, stroke, diabetes, and gastrointestinal diseases (Zheng et al., 2018; WHO, 2020). Moreover, the economic burden of smoking cannot be ignored. A systematic review was performed to assess the economic burden of smoking, and the results showed that smoking-related diseases were responsible for 1.5%–6.8% of the national health system expenditures and 0.22%–0.88% of GDP of a country (Rezaei et al., 2016). Correspondingly, in an effort to reduce smoking, the World Health Organization is supporting 100 million smokers to quit smoking for good through its “Commit to Quit” campaign, launched on World No Tobacco Day. Therefore, providing some support for smokers to quit smoking is one of the effective ways to achieve this goal.

In general, among the numerous smoking cessation interventions, pharmacological treatments are relatively mature and widespread in clinical practice. It is important to note that pharmacological therapy is a systematic intervention that includes many types of drugs, such as nicotine replacement therapy, Bupropion, and Varenicline (West, 2003; Hartmann-Boyce et al., 2018; Jordan and Xi, 2018). For these specific drugs, the smoking cessation effects have been investigated more often in randomized controlled trials, but the limitation is that the evidence is relatively isolated. Therefore, systematic reviews based on randomized controlled trials provide higher evidence for smoking cessation, especially in combination with quantitative analysis using network meta-analysis. By reviewing published studies, the results showed that in 2009, Strassmann et al. (2009) conducted a network meta-analysis of smoking cessation in chronic obstructive pulmonary disease populations, which involved nicotine replacement therapy, but comparisons between pharmacological interventions were not reported. In 2013, a network meta-analysis was published in the Cochrane Library, which comprehensively investigated the smoking cessation effects of numerous pharmacological interventions (Cahill et al., 2013). However, the randomized controlled trials included in this analysis were derived from published systematic reviews, and the scope of the search was limited to the Cochrane Database of Systematic Reviews.

Gradually, the abstinence effects of pharmacological treatments in specific populations of smokers were found in several reviews. For example, in 2004, a study by Wagena et al. (2004) first reported the effectiveness of Nicotine, Bupropion, and a combination intervention for smoking cessation in a COPD population. Similarly, several similar studies have successively reported the effects of pharmacological interventions (Jiménez-Ruiz and Fagerström, 2013; Tønnesen, 2013; Tashkin, 2015). Moreover, numerous studies have found greater benefits (e.g., lower mortality) of pharmacological interventions (nicotine and bupropion) for smoking cessation in cardiovascular disease populations, with studies finding higher rates of cessation with active treatment (Ludvig et al., 2005; Eisenberg et al., 2010; Grandi et al., 2013). Several studies have also reported the smoking cessation effect of Varenicline in patients with alcohol dependence, alcohol use disorder and alcoholism (Erwin and Slaton, 2014; Oon-Arom et al., 2019; Guo et al., 2021). Additionally, studies by Yousefi et al. (2011), Tsoi et al. (2013) found that Bupropion and Varenicline helped improve withdrawal in smokers with schizophrenia. The study by Claire et al. (2020) was based on 11 studies investigating the effect of Nicotine on smoking cessation during pregnancy. In 2021, a study by Yan and Goldman (2021) reported that Bupropion was more effective than other drugs in improving short-term adolescent withdrawal. As far as the current network meta-analysis results are concerned, in 2016, a network meta-analysis conducted by Roberts et al. (2016) reported the efficacy of pharmacotherapy for smoking cessation in adults with serious mental illness. Then in 2017, another similar study was published, while its target population was patients with cardiovascular disease (Suissa et al., 2017). In 2020, Siskind et al. (2020) also conducted a network meta-analysis reporting on pharmacological interventions for smoking cessation among people with schizophrenia spectrum disorders. However, these network meta-analyses involving smoking cessation effects in different populations are relatively limited in the number of trials included and the types of pharmacological treatments reported. Inversely, the analysis by Mishra et al. investigated the effect of pharmacological interventions for smoking cessation in healthy adults, it is worth noting that the data in this network meta-analysis came from 97 randomized controlled trials (Mishra et al., 2021). Undoubtedly, this study may miss data on non-healthy populations (e.g., diseased populations), which could affect the comprehensiveness and applicability of the findings.

Overall, there are currently fewer network meta-analyses of pharmacological interventions for smoking cessation than traditional meta-analyses (which are based more on direct comparisons). Moreover, in the published reviews, most network meta-analyses are only for specific populations or compare only a few limited drugs, which are relatively incapable of providing comprehensive and high-level evidence. Therefore, a comprehensive evidence review of the effect of pharmacological interventions on smoking cessation is of great practical importance. In this network meta-analysis, the purpose is to include all randomized controlled trials of pharmacotherapy for smoking cessation, comprehensively compare the differences in abstinence effects of different pharmacological interventions and seek the best intervention, in order to provide reference for clinical smoking cessation practice.

## Materials and methods

### Guidance and search strategy

This study was strictly conducted in accordance with the Preferred Reporting Items for Systematic Reviews and Meta-analyses (PRISMA-NMA) reporting guideline Extension Statement for Reporting of Systematic Reviews Incorporating Network Meta-analyses of Interventions (Hutton et al., 2015).

PubMed, The Cochrane Library, Web of Science, and Embase databases were searched from the date of their inception to 25 June 2022. Meanwhile, a supplementary search was conducted to obtain relevant trials by forward citation tracing and backward citation tracing of the included studies. The main search strategies were (medicine OR drug OR pharmaco*) AND (smok* OR cigarette OR tobacco OR nicotine) AND (cessation OR quit* OR abstinence OR stop* OR withdraw) AND (random* OR randomized controlled trial OR blind OR double). See Supplementary Table S1 for a more detailed presentation of the search strategy.

### Inclusion and exclusion criteria

Studies with the following criteria were included: 1) randomized controlled trials evaluating the efficacy of pharmacological interventions on smoking cessation; 2) the study population was smokers who continued to smoke while receiving treatment, where the threshold of daily smoking was identified in each trial, which was generally ≥ 1 cigarette per day; 3) the interventions included different pharmacological treatments, there was no limit to the number of arms, with at least one arm receiving one type of drug intervention, either monotherapy or combination, and 4) the main outcome measures were continuous abstinence rate or point abstinence rate, these outcomes were generally confirmed with biochemical validation based on self-reported smoking status; 5) as for duplicate publication (i.e., duplicate publication can be broadly defined as the publication of two or more articles of seemly identical material that share most of the same authors) (Lundberg, 1993), we selected the study with the largest sample size or with the longest follow-up or with the most comprehensive outcomes according to the definition of duplicate publication which were not fraudulent (e.g., a manuscript extends an original database by 50% or more) (Büchler and Farthmann, 2001).

Studies were excluded if 1) non-pharmacological interventions (e.g., behavioral interventions and some physical therapies) were used as comparators or combinations, or 2) they were fraudulent duplicate publications, had incomplete data (e.g., only continuous smoking cessation data are available), were only protocols.

### Literature selection and data extraction

Relevant literature was imported into EndNote X9 software, and duplicate articles were eliminated. Two independent reviewers screened the titles and read through the abstracts of the extracted articles for preliminary inclusion consideration. After extracting the irrelevant articles, the authors read through the full texts of the remaining studies. Trials and other irrelevant studies were further removed and reasons for exclusion were noted to identify studies that ultimately met the criteria. Each study was strictly evaluated against inclusion criteria, and any discrepancies were settled by consensus.

A pre-set standardized form was used by two reviewers who independently extracted the main information. The main data was as follows: 1) basic information, including first author, country, and year of publication. 2) The characteristics of participants, including sample size, age, gender, cigarettes per day, physical condition (with or without other diseases). 3) Intervention details, including the name of treatment, dosage of pharmacotherapy, treatment duration, process, and follow-up period. 4) Outcome indicators, including continuous abstinence rate (defined as continuous abstinence during the intervention or from the end of the intervention to the follow-up time point) or 7-day point prevalence abstinence (defined as no smoking within 7 days before the follow-up time point), validation method of abstinence (self-report or biochemical validation), the number of abstinences at different follow-up periods. For follow-up, we usually selected follow-up data for about 6 months (e.g., 24 weeks), and if the time point is not reported in a trial, data from other time points (e.g., 12 or 48 weeks) was considered.

### Risk of bias

The risk of bias in the included studies was assessed by two independent reviewers using Cochrane Collaboration’s bias risk assessment tool (Sterne et al., 2019), and disagreements were resolved by consensus. Seven domains were considered in the evaluation process, including sequence generation, allocation concealment, blinding of the participants and personnel, blinding of outcome assessments, incomplete outcome data, selective outcome reporting, and other potential sources of bias. Studies were judged to have a low-risk bias if all items were low risk. When one item had unclear risk bias, the study was rated as having an unclear risk of bias. When one item was high risk, the study was rated as having a high risk of bias (Hendarto et al., 2019; Luo et al., 2020; Li et al., 2022).

### Data analysis

Stata 15.1 software (network package and network graphs package) was used to conduct network meta-analysis (Lin et al., 2017; Xu et al., 2018). The network package performed the network meta-analysis based on the frequentist framework using random-effects models. The approach was to test the research hypothesis, as this was simpler than the problem of establishing prior probability (Hutton et al., 2014). This approach is not complex and has few limitations for ordinary researchers using network meta-analysis (Shim et al., 2017). A network diagram with nodes and lines was constructed to represent all interventions, where the size of nodes represents the number of populations, and the thickness of lines between nodes represents the number of studies. In the analysis, the number of quitters per arm and the total sample size were obtained, so the OR with 95% CI was used to estimate the effect size. The results of network meta-analysis were summarized based on all possible pairwise comparisons, including mixed comparisons (direct effects merged indirect effects) and indirect comparisons.

The node-splitting test was used to assess local inconsistency between direct and indirect comparisons. Differences between direct and indirect coefficients (*via* the *p*-value) were used to estimate the inconsistency: if *p* < 0.05, local inconsistency existed. If inconsistency was observed, non-transitivity was suspected to exist, and potential modifiers influencing treatment effect were examined (Spineli, 2019). The smoking cessation effect of different interventions was estimated based on the surface under the cumulative ranking curve. The surface under the cumulative ranking curve value ranges from 0% to 100%, where a surface under the cumulative ranking curve value of 100% indicates that the treatment was the most effective, and the smaller the value, the poorer the treatment effect.

### Certainty assessment

The quality of evidence associated with all paired comparisons was assessed using the Grades of Recommendation, Assessment, Development, and Evaluation system (Guyatt G. et al., 2011). Five downgrade factors (i.e., the risk of bias, inconsistency, imprecision, indirectness and publication bias), were considered to rate the level of evidence. Each factor was judged as “not serious” (not degraded), “serious” (degraded by one level), or “very serious” (degraded by two levels); finally, a high, moderate, low, or very low level of evidence quality was identified (Schünemann et al., 2020).

## Results

### Literature screening process and results

**FIGURE 1:**
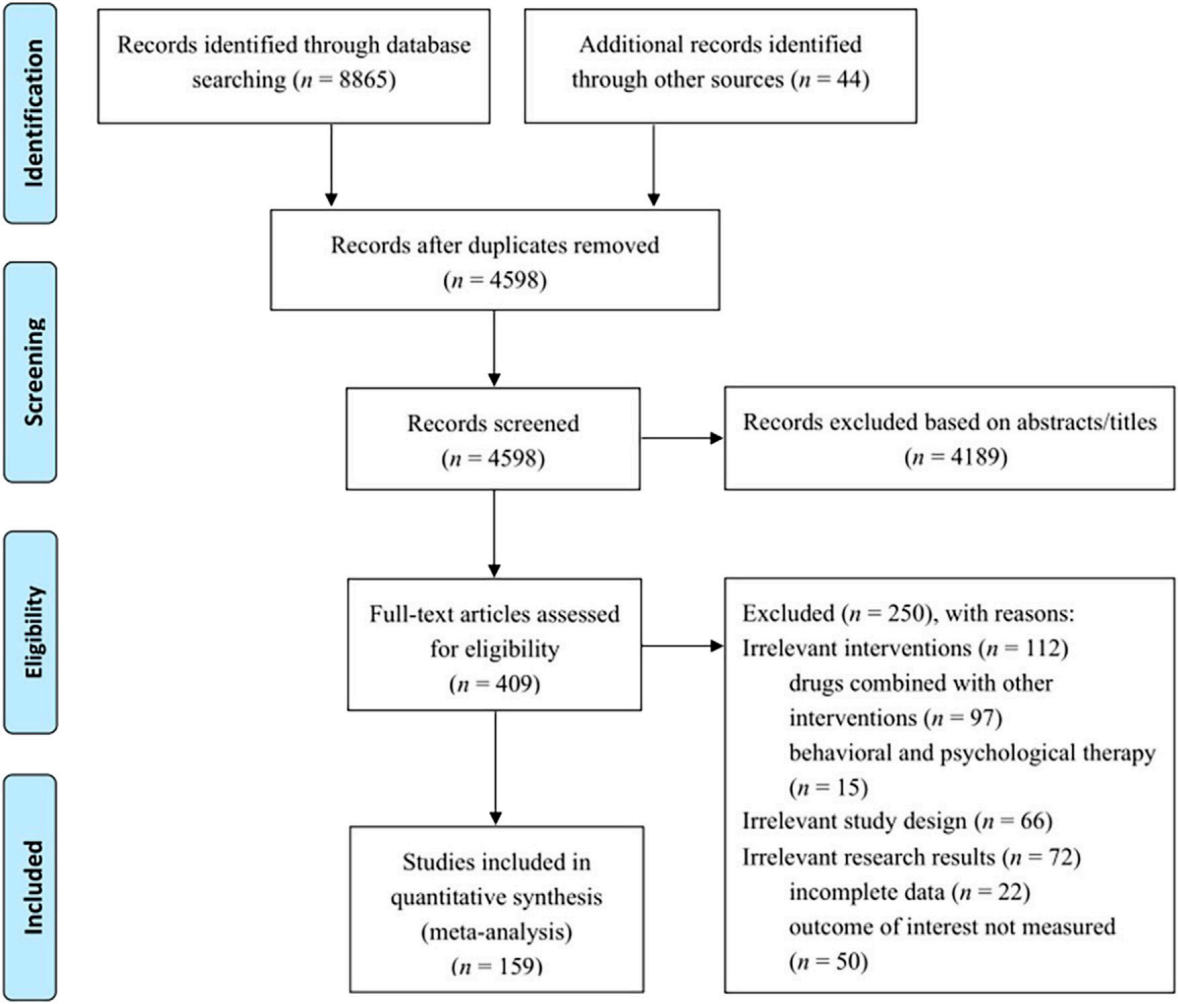
Literature screening flow chart.

As shown in Figure 1, the initial electronic search identified 8,909 potentially relevant studies. After removal of 4311 duplicate, 4,598 records were screened based on reading of titles and abstracts, which led to the exclusion of 4,189 records. Of the remaining 409 publications that were eligible for full-text review, 250 studies were excluded based on inclusion and exclusion criteria. Finally, a total of 159 randomized controlled trials, including 35 trials with backward citation tracing, were included in the network meta-analysis. A reference list of included trials is provided in the Supplementary Material.

### Description of included studies

For all included 159 trials, a total of 60,285 smokers were involved. In terms of the physical characteristics of the participants, 104 of the trials reported on the normal population, and the rest reported smokers with alcohol dependence (20 trials), mental disease (10 trials), cardiovascular diseases (seven trials), chronic obstructive pulmonary disease (five trials), pregnancy (three trials), posttraumatic stress disorder (two trials), substance use disorder (two trials), cancer (two trials), AIDS (one trial), asthma (one trial), tuberculosis (one trial), and medical disease (one trial). Moreover, a total of 15 interventions were reported, including 11 monotherapies (Bupropion, Clonidine, Cytisine, Fluoxetine, Nicotine replacement therapy, Naltrexone, Nortriptyline, Selegiline, Topiramate, Varenicline, Placebo) and four combination interventions (Bupropion + Nicotine replacement therapy, Mecamylamine + Nicotine replacement therapy, Varenicline + Bupropion, Varenicline + Nicotine replacement therapy). Meanwhile, the most of trials reported the consumption of tobacco, and the reported results showed that the average number of cigarettes smoked per day exceeded 10. The treatment duration for most trials lasted for 12 weeks (90 trials), some were 8 weeks (17 trials), 7 weeks (nine trials), 24 weeks (seven trials), 4 weeks (four trials), or 10 weeks (four trials), and the reported follow-up were centered at 24 (39 trials), 26 (10 trials), 48 (28 trials), or 53 weeks (34 trials). The outcome measure was continuous abstinence rate (137 trials) or 7-day point abstinence rate (106 trials), and the abstinence outcomes were generally confirmed with biochemical validation based on expired carbon monoxide level (140 trials), salivary cotinine concentration (12 trials), urine cotinine concentration (12 trials), serum cotinine concentration (two trials), plasma cotinine concentration (four trials), or self-reported zero smoking (two trials). See Supplementary Table S2 for more details.

### Risk of bias

A total of 16 studies were rated as high risk of bias for the incorrect randomization, non-assigned concealment, non-blinded assessment, or incomplete data. 11 studies were rated as low risk of bias, and the remaining 132 studies were rated as unclear risk of bias because of insufficient information and unclear reporting. See Supplementary Table S3 for more details.

### Network diagram

**FIGURE 2:**
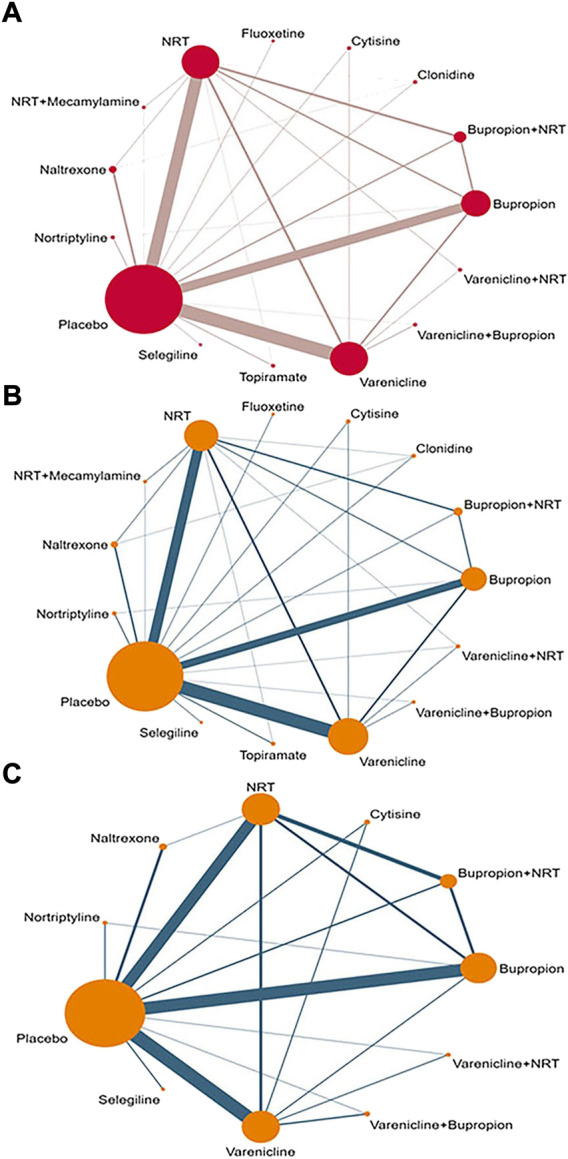
The network diagram for all interventions. **(A)** Overall abstinence rate; **(B)** Continuous abstinence rate; **(C)** 7-point continuous abstinence rate; NRT: Nicotine replacement therapy.

A network diagram was for overall abstinence rate performed based on 15 interventions, forming a total of 105 pairs of comparisons (including 28 pairs of direct comparisons and 77 pairs of indirect comparisons). In all pairwise comparisons, Varenicline vs. Placebo had the highest frequency (49 trials), followed by Nicotine replacement therapy vs. Placebo (39 trials) and Bupropion vs. Placebo (34 trials). Accordingly, population in Placebo group had the largest sample size (21,818 participants), followed by Varenicline (11,414 participants), Nicotine replacement therapy (10,671 participants), and Bupropion (7,324 participants) (Figure 2A). Furthermore, a subgroup network diagram based on continuous abstinence rate and 7-point continuous abstinence rate was performed separately. For continuous abstinence rate, the same interventions as the overall outcome were involved, the difference was that 123 studies were included (Figure 2B). For 7-point continuous abstinence rate, only 90 trials involving 11 interventions were included in the network diagram (Figure 2C).

### Inconsistency test

A global inconsistency test was performed and no difference was found (*p* = 0.825). Furthermore, to better explore local inconsistencies, a node-splitting test was conducted. A total of 28 pairwise comparisons involving loop were analyzed, the results showed that the significant inconsistency was found in Nicotine replacement therapy vs. Naltrexone (*p* = 0.017) and Naltrexone vs. Placebo (*p* = 0.011) (Table 1).

**TABLE 1:** Local inconsistency test based on side-split.

Side	Direct		Indirect		Difference		*P*>|z|
	Coef.	Std. Err.	Coef.	Std. Err.	Coef.	Std. Err.	
Bupropion - Bupropion + NRT	0.093	0.195	0.249	0.305	−0.156	0.362	0.666
Bupropion - NRT	0.040	0.219	0.081	0.131	−0.041	0.256	0.871
Bupropion - Nortriptyline	−0.151	0.575	0.215	0.398	−0.366	0.698	0.600
Bupropion - Placebo	−0.514	0.099	−0.624	0.228	0.111	0.249	0.658
Bupropion - Varenicline	0.379	0.210	0.432	0.132	−0.053	0.248	0.831
Bupropion + NRT - NRT	−0.240	0.205	0.251	0.279	−0.491	0.349	0.159
Bupropion + NRT - Placebo	−0.469	0.247	−0.826	0.218	0.357	0.333	0.284
Clonidine - NRT	0.941	0.627	0.274	0.399	0.667	0.740	0.367
Clonidine - Naltrexone	−1.117	0.797	0.190	0.459	−1.307	0.937	0.163
Clonidine - Placebo	−0.108	0.403	−0.202	0.594	0.094	0.717	0.896
Cytisine - Placebo	−0.373	0.393	−1.043	0.379	0.670	0.545	0.219
Cytisine - Varenicline	−0.080	0.370	0.590	0.401	−0.670	0.545	0.219
Fluoxetine - Placebo	−0.209	0.439	−1.059	93.837	0.850	93.838	0.993
NRT - NRT + Mecamylamine	1.415	0.749	0.404	1.651	1.011	1.857	0.586
NRT - Naltrexone	−1.929	0.599	−0.397	0.252	−1.531	0.641	0.017
NRT - Placebo	−0.618	0.092	−0.546	0.174	−0.072	0.198	0.714
NRT - Topiramate	−0.518	0.838	−0.158	0.542	−0.359	0.996	0.718
NRT - Varenicline	0.207	0.209	0.392	0.119	−0.185	0.241	0.441
NRT - Varenicline + NRT	0.720	0.421	0.528	0.371	0.192	0.561	0.732
NRT + Mecamylamine - Placebo	−1.268	0.894	−2.673	1.117	1.405	1.493	0.347
Naltrexone - Placebo	−0.188	0.237	1.664	0.685	−1.852	0.725	0.011
Nortriptyline - Placebo	−0.693	0.340	−0.077	1.019	−0.615	1.081	0.569
Placebo - Selegiline	0.135	0.635	1.182	132.188	−1.048	132.190	0.994
Placebo - Topiramate	0.525	0.503	−0.967	1.593	1.492	1.746	0.393
Placebo - Varenicline	0.982	0.086	0.792	0.188	0.189	0.208	0.361
Placebo - Varenicline + Bupropion	1.405	0.676	1.175	0.341	0.230	0.788	0.770
Varenicline - Varenicline + Bupropion	0.225	0.288	1.415	1.332	−1.190	1.363	0.382
Varenicline - Varenicline + NRT	0.188	0.355	0.380	0.434	−0.192	0.561	0.732

NRT: Nicotine replacement therapy.

### Network meta-analysis

As shown in Table 2A, the estimated effect of network meta-analysis of overall abstinence rate for each intervention on smoking cessation was generated. The network meta-analysis showed that compared with placebo, nine interventions yielded the benefits of quitting smoking, such as Varenicline [OR = 2.58, 95% CI (2.22, 3.01)] and Nicotine replacement therapy [OR = 1.83, 95% CI (1.56, 2.14)]. Meanwhile, Varenicline has greater withdrawal benefits than Naltrexone [OR = 2.61, 95% CI (1.64, 4.16)] and Clonidine [OR = 2.25, 95% CI (1.15, 4.39)]. Then, Varenicline combined with Nicotine replacement therapy is superior to monotherapy such as Bupropion [OR = 1.98, 95% CI (1.13, 3.48)], Nicotine replacement therapy [OR = 1.84, 95% CI (1.07, 3.18)], Naltrexone [OR = 3.40, 95% CI (1.69, 6.84)], and Clonidine [OR = 2.94, 95% CI (1.26, 6.83)] in smoking cessation. In addition, Varenicline + Bupropion intervention was also superior to Naltrexone [OR = 3.45, 95% CI (1.68, 7.07)], Nicotine replacement therapy [OR = 1.87, 95% CI (1.04, 3.35)], Clonidine [OR = 2.97, 95% CI (1.25, 7.05)] and Bupropion [OR = 2.00, 95% CI (1.11, 3.61)]. Compared with Naltrexone, the abstinence superiority was found in Bupropion [OR = 1.72, 95% CI (1.07, 2.76)], Nicotine replacement therapy [OR = 1.84, 95% CI (1.16, 2.93)], Cytisine [OR = 2.08, 95% CI (1.04, 4.16)], and Bupropion + Nicotine replacement therapy [OR = 1.97, 95% CI (1.15, 3.39)]. Moreover, Nicotine replacement therapy + Mecamylamine was superior to Naltrexone [OR = 6.29, 95% CI (1.59, 24.90)], Fluoxetine [OR = 5.05, 95% CI (1.06, 24.12)] and Clonidine [OR = 5.43, 95% CI (1.27, 23.26)]. It is worth noting that Varenicline was superior to Nicotine replacement therapy [OR = 1.42, 95% CI (1.16, 1.73)] and Bupropion [OR = 1.50, 95% CI (1.22, 1.89)].

**TABLE 2:** The results of network meta-analysis for all pairwise comparisons.

(A) Overall abstinence rate														
**VAR + NRT**	1.01 (0.47, 2.18)	0.77 (0.45, 1.31)	0.42 (0.15, 1.17)	0.34 (0.09, 1.32)	0.30 (0.17, 0.51)	0.56 (0.24, 1.27)	0.29 (0.15, 0.59)	1.85 (0.45, 7.55)	0.54 (0.31, 0.93)	0.37 (0.13, 1.01)	0.61 (0.29, 1.30)	0.34 (0.15, 0.79)	0.58 (0.31, 1.08)	0.51 (0.29, 0.89)
0.99 (0.46, 2.13)	**VAR + BUP**	0.76 (0.44, 1.31)	0.41 (0.14, 1.18)	0.34 (0.09, 1.32)	0.29 (0.17, 0.52)	0.55 (0.24, 1.28)	0.29 (0.14, 0.60)	1.83 (0.44, 7.56)	0.54 (0.30, 0.96)	0.36 (0.13, 1.01)	0.60 (0.28, 1.30)	0.34 (0.14, 0.80)	0.57 (0.30, 1.09)	0.50 (0.28, 0.90)
1.30 (0.76, 2.23)	1.32 (0.76, 2.29)	**Varenicline**	0.54 (0.22, 1.33)	0.44 (0.13, 1.55)	0.39 (0.33, 0.45)	0.73 (0.38, 1.38)	0.38 (0.24, 0.61)	2.41 (0.65, 8.95)	0.71 (0.58, 0.87)	0.48 (0.20, 1.14)	0.80 (0.47, 1.36)	0.44 (0.23, 0.87)	0.76 (0.54, 1.07)	0.66 (0.53, 0.82)
2.40 (0.85, 6.77)	2.43 (0.85, 6.96)	1.84 (0.75, 4.52)	**Topiramate**	0.82 (0.18, 3.76)	0.71 (0.29, 1.73)	1.34 (0.45, 3.95)	0.71 (0.26, 1.90)	4.44 (0.92, 21.44)	1.30 (0.53, 3.18)	0.88 (0.26, 3.02)	1.47 (0.52, 4.13)	0.82 (0.27, 2.45)	1.39 (0.55, 3.56)	1.21 (0.49, 2.99)
2.94 (0.76, 11.44)	2.98 (0.76, 11.70)	2.26 (0.64, 7.91)	1.23 (0.27, 5.64)	**Selegiline**	0.87 (0.25, 3.03)	1.64 (0.41, 6.59)	0.86 (0.23, 3.24)	5.44 (0.90, 32.99)	1.60 (0.45, 5.59)	1.08 (0.24, 4.89)	1.80 (0.46, 6.97)	1.00 (0.25, 4.08)	1.71 (0.47, 6.16)	1.49 (0.42, 5.22)
3.37 (1.96, 5.80)	3.41 (1.93, 6.02)	2.58 (2.22, 3.01)	1.40 (0.58, 3.40)	1.14 (0.33, 3.97)	**Placebo**	1.87 (1.00, 3.50)	0.99 (0.64, 1.54)	6.22 (1.69, 22.94)	1.83 (1.56, 2.14)	1.23 (0.52, 2.92)	2.06 (1.20, 3.52)	1.15 (0.60, 2.20)	1.95 (1.42, 2.68)	1.70 (1.43, 2.03)
1.80 (0.79, 4.12)	1.82 (0.78, 4.24)	1.38 (0.72, 2.63)	0.75 (0.25, 2.22)	0.61 (0.15, 2.46)	0.53 (0.29, 1.00)	**Nortriptyline**	0.53 (0.25, 1.14)	3.32 (0.78, 14.12)	0.97 (0.51, 1.86)	0.66 (0.23, 1.91)	1.10 (0.48, 2.50)	0.61 (0.25, 1.51)	1.04 (0.52, 2.09)	0.91 (0.48, 1.72)
3.40 (1.69, 6.84)	3.45 (1.68, 7.07)	2.61 (1.64, 4.16)	1.42 (0.53, 3.81)	1.16 (0.31, 4.33)	1.01 (0.65, 1.57)	1.89 (0.88, 4.07)	**Naltrexone**	6.29 (1.59, 24.90)	1.84 (1.16, 2.93)	1.24 (0.47, 3.28)	2.08 (1.04, 4.16)	1.16 (0.54, 2.49)	1.97 (1.15, 3.39)	1.72 (1.07, 2.76)
0.54 (0.13, 2.21)	0.55 (0.13, 2.27)	0.42 (0.11, 1.54)	0.23 (0.05, 1.09)	0.18 (0.03, 1.12)	0.16 (0.04, 0.59)	0.30 (0.07, 1.28)	0.16 (0.04, 0.63)	**NRT + MEC**	0.29 (0.08, 1.08)	0.20 (0.04, 0.95)	0.33 (0.08, 1.35)	0.18 (0.04, 0.79)	0.31 (0.08, 1.19)	0.27 (0.07, 1.02)
1.84 (1.07, 3.18)	1.87 (1.04, 3.35)	1.42 (1.16, 1.73)	0.77 (0.31, 1.88)	0.63 (0.18, 2.20)	0.55 (0.47, 0.64)	1.03 (0.54, 1.96)	0.54 (0.34, 0.86)	3.41 (0.93, 12.54)	**NRT**	0.67 (0.28, 1.62)	1.13 (0.65, 1.97)	0.63 (0.32, 1.22)	1.07 (0.78, 1.47)	0.93 (0.75, 1.16)
2.73 (0.99, 7.57)	2.77 (0.99, 7.76)	2.10 (0.87, 5.03)	1.14 (0.33, 3.92)	0.93 (0.20, 4.22)	0.81 (0.34, 1.92)	1.52 (0.52, 4.41)	0.80 (0.31, 2.11)	5.05 (1.06, 24.12)	1.48 (0.62, 3.56)	**Fluoxetine**	1.67 (0.60, 4.61)	0.93 (0.32, 2.74)	1.58 (0.63, 3.97)	1.38 (0.57, 3.33)
1.64 (0.77, 3.48)	1.66 (0.77, 3.57)	1.26 (0.73, 2.15)	0.68 (0.24, 1.92)	0.56 (0.14, 2.16)	0.49 (0.28, 0.83)	0.91 (0.40, 2.08)	0.48 (0.24, 0.96)	3.03 (0.74, 12.39)	0.89 (0.51, 1.55)	0.60 (0.22, 1.65)	**Cytisine**	0.56 (0.24, 1.30)	0.95 (0.51, 1.77)	0.83 (0.47, 1.45)
2.94 (1.26, 6.83)	2.97 (1.25, 7.05)	2.25 (1.15, 4.39)	1.22 (0.41, 3.67)	1.00 (0.25, 4.06)	0.87 (0.45, 1.67)	1.63 (0.66, 4.03)	0.86 (0.40, 1.85)	5.43 (1.27, 23.26)	1.59 (0.82, 3.08)	1.07 (0.36, 3.16)	1.79 (0.77, 4.17)	**Clonidine**	1.70 (0.83, 3.50)	1.48 (0.76, 2.91)
1.72 (0.93, 3.20)	1.75 (0.92, 3.33)	1.32 (0.94, 1.87)	0.72 (0.28, 1.83)	0.59 (0.16, 2.12)	0.51 (0.37, 0.70)	0.96 (0.48, 1.93)	0.51 (0.30, 0.87)	3.19 (0.84, 12.14)	0.93 (0.68, 1.29)	0.63 (0.25, 1.58)	1.05 (0.57, 1.96)	0.59 (0.29, 1.21)	**BUP + NRT**	0.87 (0.63, 1.20)
1.98 (1.13, 3.48)	2.00 (1.11, 3.61)	1.52 (1.22, 1.89)	0.82 (0.33, 2.03)	0.67 (0.19, 2.36)	0.59 (0.49, 0.70)	1.10 (0.58, 2.09)	0.58 (0.36, 0.94)	3.66 (0.98, 13.62)	1.07 (0.86, 1.34)	0.72 (0.30, 1.74)	1.21 (0.69, 2.12)	0.67 (0.34, 1.32)	1.15 (0.83, 1.58)	**Bupropion**

(B) Continuous abstinence rate														
**VAR + NRT**	1.08 (0.51, 2.32)	0.83 (0.51, 1.34)	0.45 (0.17, 1.19)	0.41 (0.12, 1.41)	0.31 (0.19, 0.51)	0.58 (0.28, 1.23)	0.31 (0.15, 0.63)	2.04 (0.53, 7.84)	0.63 (0.38, 1.04)	0.39 (0.15, 0.98)	0.63 (0.32, 1.24)	0.36 (0.17, 0.78)	0.57 (0.31, 1.04)	0.58 (0.35, 0.96)
0.92 (0.43, 1.98)	**VAR + BUP**	0.76 (0.42, 1.39)	0.41 (0.15, 1.17)	0.37 (0.10, 1.37)	0.29 (0.16, 0.53)	0.54 (0.24, 1.24)	0.29 (0.13, 0.63)	1.88 (0.47, 7.61)	0.58 (0.31, 1.09)	0.36 (0.13, 0.97)	0.58 (0.27, 1.25)	0.33 (0.14, 0.78)	0.53 (0.26, 1.07)	0.53 (0.28, 1.00)
1.21 (0.75, 1.95)	1.31 (0.72, 2.37)	**Varenicline**	0.54 (0.23, 1.27)	0.49 (0.15, 1.55)	0.38 (0.33, 0.44)	0.71 (0.39, 1.26)	0.37 (0.22, 0.64)	2.46 (0.69, 8.74)	0.76 (0.62, 0.94)	0.47 (0.21, 1.05)	0.76 (0.48, 1.23)	0.44 (0.24, 0.81)	0.69 (0.47, 1.02)	0.70 (0.56, 0.87)
2.23 (0.84, 5.92)	2.42 (0.85, 6.87)	1.85 (0.78, 4.37)	**Topiramate**	0.91 (0.22, 3.76)	0.70 (0.30, 1.64)	1.31 (0.47, 3.62)	0.69 (0.26, 1.86)	4.56 (1.00, 20.74)	1.41 (0.60, 3.32)	0.87 (0.27, 2.77)	1.42 (0.54, 3.74)	0.81 (0.29, 2.28)	1.28 (0.51, 3.21)	1.29 (0.54, 3.06)
2.47 (0.71, 8.58)	2.68 (0.73, 9.81)	2.05 (0.64, 6.51)	1.10 (0.27, 4.60)	**Selegiline**	0.78 (0.25, 2.44)	1.44 (0.40, 5.18)	0.77 (0.22, 2.69)	5.04 (0.92, 27.67)	1.56 (0.49, 4.97)	0.96 (0.24, 3.87)	1.56 (0.45, 5.41)	0.89 (0.25, 3.26)	1.42 (0.42, 4.71)	1.42 (0.45, 4.55)
3.18 (1.96, 5.17)	3.45 (1.88, 6.34)	2.64 (2.27, 3.06)	1.42 (0.61, 3.32)	1.29 (0.41, 4.06)	**Placebo**	1.86 (1.06, 3.28)	0.99 (0.59, 1.64)	6.50 (1.84, 22.88)	2.01 (1.69, 2.38)	1.23 (0.56, 2.73)	2.02 (1.25, 3.25)	1.15 (0.64, 2.09)	1.82 (1.27, 2.63)	1.83 (1.52, 2.21)
1.71 (0.81, 3.60)	1.85 (0.81, 4.25)	1.42 (0.79, 2.54)	0.77 (0.28, 2.12)	0.69 (0.19, 2.49)	0.54 (0.31, 0.95)	**Nortriptyline**	0.53 (0.25, 1.14)	3.49 (0.88, 13.88)	1.08 (0.60, 1.95)	0.66 (0.25, 1.76)	1.08 (0.52, 2.27)	0.62 (0.27, 1.41)	0.98 (0.50, 1.91)	0.99 (0.55, 1.77)
3.22 (1.60, 6.52)	3.50 (1.58, 7.74)	2.67 (1.57, 4.55)	1.44 (0.54, 3.88)	1.31 (0.37, 4.59)	1.01 (0.61, 1.69)	1.89 (0.88, 4.04)	**Naltrexone**	6.59 (1.70, 25.59)	2.04 (1.20, 3.46)	1.25 (0.49, 3.22)	2.04 (1.02, 4.11)	1.17 (0.55, 2.49)	1.85 (0.99, 3.45)	1.86 (1.08, 3.20)
0.49 (0.13, 1.88)	0.53 (0.13, 2.15)	0.41 (0.11, 1.44)	0.22 (0.05, 1.00)	0.20 (0.04, 1.09)	0.15 (0.04, 0.54)	0.29 (0.07, 1.14)	0.15 (0.04, 0.59)	**NRT + MEC**	0.31 (0.09, 1.09)	0.19 (0.04, 0.84)	0.31 (0.08, 1.19)	0.18 (0.04, 0.71)	0.28 (0.08, 1.03)	0.28 (0.08, 1.01)
1.58 (0.96, 2.61)	1.72 (0.92, 3.21)	1.31 (1.07, 1.61)	0.71 (0.30, 1.67)	0.64 (0.20, 2.04)	0.50 (0.42, 0.59)	0.93 (0.51, 1.67)	0.49 (0.29, 0.84)	3.23 (0.92, 11.36)	**NRT**	0.61 (0.27, 1.39)	1.00 (0.61, 1.66)	0.57 (0.31, 1.06)	0.91 (0.63, 1.31)	0.91 (0.72, 1.15)
2.58 (1.02, 6.55)	2.80 (1.03, 7.62)	2.14 (0.95, 4.80)	1.15 (0.36, 3.69)	1.04 (0.26, 4.22)	0.81 (0.37, 1.80)	1.51 (0.57, 4.00)	0.80 (0.31, 2.06)	5.27 (1.19, 23.36)	1.63 (0.72, 3.67)	**Fluoxetine**	1.63 (0.65, 4.13)	0.93 (0.35, 2.53)	1.48 (0.62, 3.55)	1.49 (0.66, 3.37)
1.58 (0.81, 3.08)	1.71 (0.80, 3.66)	1.31 (0.81, 2.10)	0.71 (0.27, 1.86)	0.64 (0.18, 2.21)	0.50 (0.31, 0.80)	0.92 (0.44, 1.93)	0.49 (0.24, 0.98)	3.22 (0.84, 12.36)	1.00 (0.60, 1.64)	0.61 (0.24, 1.55)	**Cytisine**	0.57 (0.27, 1.22)	0.90 (0.50, 1.64)	0.91 (0.55, 1.51)
2.76 (1.28, 5.94)	2.99 (1.28, 7.01)	2.29 (1.24, 4.22)	1.23 (0.44, 3.47)	1.12 (0.31, 4.07)	0.87 (0.48, 1.57)	1.61 (0.71, 3.67)	0.86 (0.40, 1.82)	5.63 (1.40, 22.61)	1.74 (0.95, 3.20)	1.07 (0.40, 2.89)	1.75 (0.82, 3.74)	**Clonidine**	1.58 (0.79, 3.17)	1.59 (0.85, 2.97)
1.74 (0.96, 3.18)	1.89 (0.93, 3.83)	1.45 (0.98, 2.13)	0.78 (0.31, 1.96)	0.71 (0.21, 2.35)	0.55 (0.38, 0.79)	1.02 (0.52, 1.99)	0.54 (0.29, 1.01)	3.56 (0.97, 13.13)	1.10 (0.76, 1.59)	0.68 (0.28, 1.62)	1.11 (0.61, 2.01)	0.63 (0.32, 1.27)	**BUP + NRT**	1.01 (0.70, 1.45)
1.73 (1.04, 2.90)	1.88 (1.00, 3.53)	1.44 (1.15, 1.79)	0.78 (0.33, 1.84)	0.70 (0.22, 2.24)	0.55 (0.45, 0.66)	1.01 (0.57, 1.82)	0.54 (0.31, 0.93)	3.54 (0.99, 12.61)	1.09 (0.87, 1.38)	0.67 (0.30, 1.52)	1.10 (0.66, 1.82)	0.63 (0.34, 1.17)	0.99 (0.69, 1.43)	**Bupropion**

(C) 7-point continuous abstinence rate										
**VAR + NRT**	0.95 (0.41, 2.19)	0.87 (0.46, 1.62)	0.42 (0.10, 1.73)	0.37 (0.19, 0.70)	0.56 (0.22, 1.46)	0.40 (0.17, 0.97)	0.55 (0.28, 1.08)	0.77 (0.34, 1.75)	0.88 (0.41, 1.87)	0.64 (0.32, 1.25)
1.05 (0.46, 2.43)	**VAR + BUP**	0.91 (0.52, 1.60)	0.44 (0.11, 1.78)	0.39 (0.21, 0.69)	0.59 (0.24, 1.48)	0.43 (0.18, 0.98)	0.58 (0.31, 1.08)	0.81 (0.37, 1.76)	0.92 (0.46, 1.87)	0.67 (0.36, 1.25)
1.15 (0.62, 2.15)	1.09 (0.63, 1.91)	**Varenicline**	0.49 (0.14, 1.74)	0.42 (0.34, 0.52)	0.65 (0.31, 1.35)	0.47 (0.25, 0.88)	0.63 (0.48, 0.84)	0.88 (0.51, 1.52)	1.01 (0.65, 1.58)	0.73 (0.54, 0.98)
2.37 (0.58, 9.77)	2.25 (0.56, 9.04)	2.06 (0.57, 7.39)	**Selegiline**	0.87 (0.25, 3.06)	1.33 (0.31, 5.64)	0.96 (0.24, 3.87)	1.31 (0.36, 4.69)	1.82 (0.46, 7.17)	2.08 (0.56, 7.79)	1.51 (0.42, 5.41)
2.73 (1.43, 5.21)	2.59 (1.44, 4.67)	2.37 (1.92, 2.94)	1.15 (0.33, 4.06)	**Placebo**	1.53 (0.76, 3.10)	1.11 (0.61, 2.01)	1.50 (1.20, 1.88)	2.10 (1.21, 3.62)	2.40 (1.60, 3.58)	1.74 (1.39, 2.16)
1.78 (0.69, 4.63)	1.69 (0.68, 4.23)	1.55 (0.74, 3.22)	0.75 (0.18, 3.18)	0.65 (0.32, 1.32)	**Nortriptyline**	0.72 (0.29, 1.81)	0.98 (0.47, 2.05)	1.37 (0.56, 3.33)	1.56 (0.70, 3.49)	1.13 (0.55, 2.33)
2.47 (1.03, 5.96)	2.35 (1.02, 5.42)	2.15 (1.14, 4.05)	1.04 (0.26, 4.20)	0.90 (0.50, 1.65)	1.39 (0.55, 3.50)	**Naltrexone**	1.36 (0.72, 2.56)	1.90 (0.84, 4.26)	2.17 (1.06, 4.44)	1.57 (0.83, 2.97)
1.82 (0.93, 3.57)	1.72 (0.93, 3.20)	1.58 (1.19, 2.10)	0.77 (0.21, 2.75)	0.66 (0.53, 0.83)	1.02 (0.49, 2.13)	0.73 (0.39, 1.38)	**NRT**	1.39 (0.78, 2.50)	1.59 (1.08, 2.36)	1.15 (0.87, 1.53)
1.30 (0.57, 2.97)	1.24 (0.57, 2.69)	1.13 (0.66, 1.95)	0.55 (0.14, 2.16)	0.48 (0.28, 0.82)	0.73 (0.30, 1.78)	0.53 (0.23, 1.18)	0.72 (0.40, 1.29)	**Cytisine**	1.14 (0.58, 2.24)	0.83 (0.46, 1.49)
1.14 (0.54, 2.43)	1.08 (0.53, 2.19)	0.99 (0.63, 1.54)	0.48 (0.13, 1.80)	0.42 (0.28, 0.62)	0.64 (0.29, 1.43)	0.46 (0.23, 0.94)	0.63 (0.42, 0.93)	0.87 (0.45, 1.71)	**BUP + NRT**	0.72 (0.49, 1.08)
1.57 (0.80, 3.10)	1.49 (0.80, 2.78)	1.37 (1.02, 1.84)	0.66 (0.18, 2.38)	0.58 (0.46, 0.72)	0.88 (0.43, 1.82)	0.64 (0.34, 1.20)	0.87 (0.65, 1.15)	1.21 (0.67, 2.17)	1.38 (0.92, 2.06)	**Bupropion**

BUP: Bupropion; NRT: Nicotine replacement therapy; MEC: Mecamylamine; VAR: Varenicline.

Note: All effect sizes were presented using OR values and 95% confidence intervals.

In each column, each effect size was the result of that intervention compared to any other intervention.

A subgroup network meta-analysis based on different outcomes and populations was conducted. For subcategory outcomes, continuous abstinence rate and 7-point continuous abstinence rate were analyzed separately. In the continuous abstinence rate, a total of 15 interventions were involved, the network meta-analysis results showed that Varenicline + Nicotine replacement therapy, Varenicline + Bupropion, and Nicotine replacement therapy + Mecamylamine all were superior to Placebo, Naltrexone, Fluoxetine, and Clonidine (See Table 2B). In the 7-point continuous abstinence rate, a total of 11 interventions were involved, the network meta-analysis results showed that Varenicline + Nicotine replacement therapy, Varenicline + Bupropion, and Bupropion + Nicotine replacement therapy all were superior to Placebo and Naltrexone. Moreover, Varenicline monotherapy was superior to Nicotine replacement therapy [OR = 1.58, 95% CI (1.19, 2.09)] and Bupropion [OR = 1.37, 95% CI (1.02, 1.83)] (See Table 2C). For the subcategory populations, more specific types of smokers, such as alcohol dependence, chronic obstructive pulmonary disease, and mental disorders were analyzed independently. In these specific populations, the main reported interventions included Varenicline, Bupropion, Nicotine replacement therapy, and Placebo. In general, both Varenicline and Bupropion were superior to Placebo in most populations, but in smokers with asthma and cancer, neither Varenicline vs. Placebo nor Bupropion vs. Placebo showed significant statistical differences. Interestingly, Bupropion combined with Nicotine replacement therapy was found to be superior to Placebo [OR = 16.14, 95% CI (2.91, 89.58)] and Bupropion [OR = 6.86, 95% CI (1.23, 38.29)] in a population with mental illness. See Table 3 for more details.

**TABLE 3:** Meta-analysis of smoking cessation effects of different interventions in specific populations of smokers.

Population	Comparison	Relation	Study	OR	95% CI	*p* Value
COPD	VAR vs. PLA	Direct	2	4.46	(3.21, 6.21)	*p* < 0.05
	BUP vs. PLA	Direct	1	2.26	(1.07, 4.81)	*p* < 0.05
	NOR vs. PLA	Direct	1	1.95	(0.90, 4.23)	*p >* 0.05
	NOR vs. BUP	Direct	1	0.53	(0.09, 3.00)	*p >* 0.05
	VAR vs. BUP	Direct	1	2.47	(1.23, 4.92)	*p* < 0.05
	VAR vs. NOR	Indirect	—	1.9	(0.33, 10.82)	*p >* 0.05
Mental illness	VAR vs. PLA	Direct	5	3.03	(2.04, 4.51)	*p* < 0.05
	BUP vs. PLA	Direct	4	2.56	(1.40, 4.68)	*p* < 0.05
	BUP + NRT vs. NRT	Direct	1	3	(0.52, 17.16)	*p >* 0.05
	NRT vs. PLA	Direct	1	3.42	(1.78, 6.56)	*p* < 0.05
	VAR vs. BUP	Direct	1	2.29	(1.34, 3.88)	*p* < 0.05
	VAR vs. NRT	Direct	1	1.35	(0.83, 2.19)	*p >* 0.05
	BUP vs. NRT	Direct	2	2.08	(0.12, 36.42)	*p >* 0.05
	BUP + NRT vs. PLA	Indirect	—	16.14	(2.91, 89.58)	*p* < 0.05
	BUP + NRT vs. VAR	Indirect	—	4.75	(0.88, 25.51)	*p >* 0.05
	BUP + NRT vs. BUP	Indirect	—	6.86	(1.23, 38.29)	*p* < 0.05
Cardiovascular disease	BUP vs. PLA	Direct	4	1.94	(1.03, 3.66)	*p* < 0.05
	VAR vs. PLA	Direct	2	2.47	(1.07, 5.70)	*p* < 0.05
	NRT vs. PLA	Direct	1	1.97	(0.97, 4.01)	*p >* 0.05
	VAR vs. BUP	Indirect	—	1.88	(0.44, 8.04)	*p >* 0.05
Cancer	VAR vs. PLA	Direct	1	1.16	(0.58, 2.30)	*p >* 0.05
	BUP vs. PLA	Direct	1	1.07	(0.56, 2.06)	*p >* 0.05
HIV	VAR vs. PLA	Direct	1	2.51	(1.05, 6.01)	*p* < 0.05
Posttraumatic Stress Disorder	BUP vs. PLA	Direct	1	2.67	(0.21, 33.49)	*p >* 0.05
	NRT vs. PLA	Direct	1	2.07	(0.35, 12.22)	*p >* 0.05
Substance Use Disorders	VAR vs. NRT	Direct	2	3.61	(1.17, 11.13)	*p* < 0.05
Asthma	VAR vs. PLA	Direct	1	1.25	(0.29, 5.31)	*p >* 0.05
Tuberculosis	CYT vs. PLA	Direct	1	1.09	(0.93, 1.28)	*p >* 0.05
Medical illnesses smokers	BUP + NRT vs. NRT	Direct	1	2.33	(1.03, 5.25)	*p >* 0.05
Alcohol dependence	VAR vs. PLA	Direct	6	2.71	(1.32, 5.58)	*p* < 0.05
	NAL vs. PLA	Direct	4	1.36	(0.81, 2.28)	*p >* 0.05
	BUP vs. PLA	Direct	3	0.73	(0.41, 1.29)	*p >* 0.05
	TOP vs. PLA	Direct	2	1.3	(0.56, 3.02)	*p >* 0.05
	NRT vs. PLA	Direct	1	3.09	(1.12, 8.54)	*p* < 0.05
	VAR + NRT vs. NRT	Direct	1	2.06	(0.97, 4.37)	*p >* 0.05
	VAR vs. CYT	Direct	1	0.65	(0.31, 1.38)	*p >* 0.05
	NRT vs. NAL	Indirect	—	2.18	(0.57, 8.37)	*p >* 0.05
	BUP vs. NAL	Indirect	—	0.5	(0.18, 1.36)	*p >* 0.05
	NAL vs. VAR	Indirect	—	0.7	(0.23, 2.14)	*p >* 0.05
	NAL vs. VAR + NRT	Indirect	—	0.22	(0.04, 1.15)	*p >* 0.05
	NAL vs. TOP	Indirect	—	0.41	(0.34, 3.59)	*p >* 0.05
	NRT vs. VAR	Indirect	—	1.53	(0.36, 6.53)	*p >* 0.05
	NRT vs. TOP	Indirect	—	2.4	(0.54, 10.70)	*p >* 0.05
	BUP VS. VAR + NRT	Indirect	—	0.11	(0.02, 0.57)	*p >* 0.05
	BUP VS. VAR	Indirect	—	0.35	(0.11, 1.11)	*p >* 0.05
	BUP VS. TOP	Indirect	—	0.54	(0.16, 1.80)	*p >* 0.05
	VAR vs. VAR + NRT	Indirect	—	0.32	(0.06, 1.79)	*p >* 0.05
	TOP vs. VAR + NRT	Indirect	—	0.2	(0.03, 1.18)	*p >* 0.05
	TOP vs. VAR	Indirect	—	0.64	(0.17, 2.34)	*p >* 0.05
	BUP vs. NRT	Indirect	—	0.23	(0.06, 0.88)	*p >* 0.05
	PLA vs. VAR + NRT	Indirect	—	0.16	(0.04, 0.69)	*p >* 0.05
	BUP vs. CYT	Indirect	—	0.23	(0.05, 1.00)	*p >* 0.05
	CYT vs. TOP	Indirect	—	2.41	(0.49, 11.86)	*p >* 0.05
	CYT vs. PLA	Indirect	—	3.11	(0.87, 11.17)	*p >* 0.05
	CYT vs. NAL	Indirect	—	2.19	(0.51, 9.34)	*p >* 0.05
	CYT vs. NRT	Indirect	—	1.01	(0.18, 5.63)	*p >* 0.05
	CYT vs. VAR + NRT	Indirect	—	0.49	(0.07, 3.46)	*p >* 0.05

COPD: Chronic Obstructive Pulmonary Disease; Bupropion: BUP; NRT: Nicotine replacement therapy; Cytisine: CYT; Naltrexone: NAL; Topiramate: TOP; Placebo: PLA; Nortriptyline: NOR; Varenicline: VAR.

### Probability ranking

**FIGURE 3:**
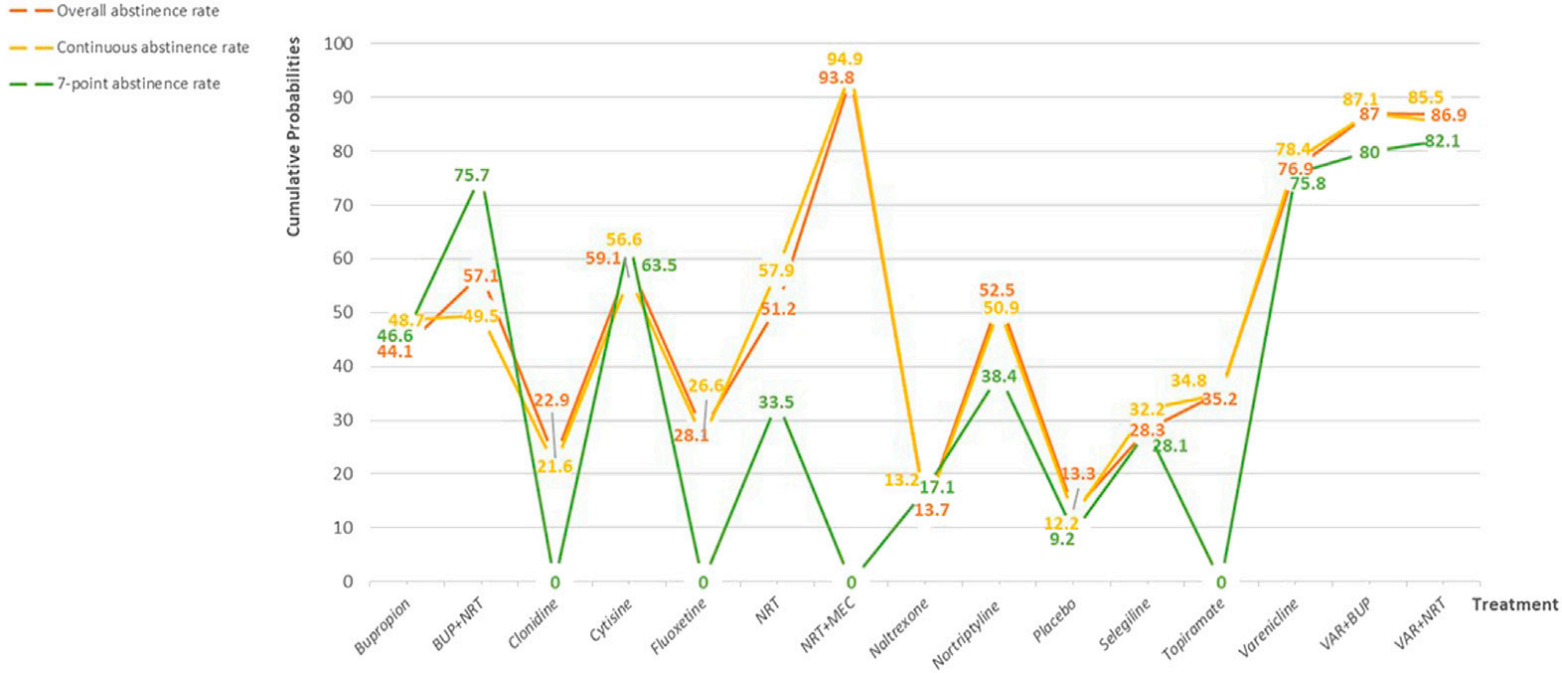
Probability ranking for all interventions. NRT: Nicotine replacement therapy; VAR: Varenicline; BUP: Bupropion.

The surface under the cumulative ranking curve for all interventions is showed in Figure 3. For overall abstinence rate, the value predicted the possibility of different interventions as the best treatment and the result showed that Mecamylamine + Nicotine replacement therapy had the greatest probability (93.8%) of becoming the best intervention. For other interventions, the smoking cessation effect were ranked as follows, Varenicline + Bupropion (87.0%), Varenicline + Nicotine replacement therapy (86.9%), Varenicline (76.9%), Cytisine (59.1%), Bupropion + Nicotine replacement therapy (57.1%), Nortriptyline (52.5%), Nicotine replacement therapy (51.2%), Bupropion (44.1%), Topiramate (35.2%), Selegiline (28.3%), Fluoxetine (28.1%), Clonidine (22.9%), Naltrexone (13.7%), and Placebo (13.3%). In the continuous abstinence rate, the first four interventions were in the same order as overall abstinence rate, the difference was that Naltrexone was in fifth place (57.9%). In 7-point continuous abstinence rate, Varenicline + Nicotine replacement therapy had the greatest probability (82.1%) of becoming the best intervention, followed by Varenicline + Bupropion (80.0%).

### Certainty of evidence

The evidence quality for all 105 comparisons was evaluated by the GRADE system. In the comparison of 28 pairs of mixed effects (the combination of direct and indirect effects), eight as moderate-level evidence, five as low-level evidence, and 15 as very-low-level evidence. For 77 pairs of indirect comparisons, 23 comparisons were rated as moderate-level evidence, 27 as low-level evidence, and 27 as very-low-level evidence. See Supplementary Table S4 for more details.

## Discussion

For all interventions and associated pair-wise comparisons, the network meta-analysis results showed that most interventions yielded the benefits of smoking cessation compared with placebo, whether monotherapy or combination therapy. Meanwhile, the results showed that Varenicline, Bupropion, Nicotine replacement therapy, Varenicline + Nicotine replacement therapy, Varenicline + Bupropion, Mecamylamine + Nicotine replacement therapy, and Bupropion + Nicotine replacement therapy were superior to Naltrexone. It was worth mentioning that, in the probability ranking of all interventions, only Naltrexone was similar to placebo, which means that it has the smallest probability of being the best intervention. Therefore, it is necessary to investigate the difference in effect between Naltrexone and placebo. The network meta-analysis in this study showed that no significant differences were found between Naltrexone and placebo. Similarly, as early as 2006, a systematic review published in the Cochrane Library evaluated the efficacy of opioid antagonists (Naltrexone) in promoting long-term smoking cessation, the review failed to detect a significant difference in quit rates between naltrexone and placebo based on the synthesis of the four trials (David et al., 2006). Moreover, a systematic review by David et al. (2014) in 2014 further confirmed no beneficial effect of naltrexone on short-term or long-term smoking abstinence. In the comparison of other monotherapy, it is notable that Varenicline is superior to Nicotine replacement therapy, Clonidine and Bupropion. Among them, a network meta-analysis by Cahill et al. supported our findings, and their results showed that Varenicline was superior to single forms of Nicotine replacement therapy, and to Bupropion (Cahill et al., 2013). For all monotherapies in the analysis, Bupropion, Fluoxetine, and Nortriptyline all are antidepressants, the results showed the antidepressants Bupropion and Nortriptyline aided long-term smoking cessation, the same finding was reported in a review published in the Cochrane Library (Hughes et al., 2014).

In addition, the smoking cessation effect of some combined interventions was worthy of attention. In this network meta-analysis, four combined interventions were reported, including Varenicline + Nicotine replacement therapy, Varenicline + Bupropion, Nicotine replacement therapy + Mecamylamine, and Bupropion + Nicotine replacement therapy. For Varenicline + Nicotine replacement therapy, our analysis showed that it was superior to the six monotherapies, and it was third in the probability ranking. Differently, in a recent network meta-analysis, the authors determined the clinical effectiveness of smoking cessation medicines and e-cigarettes, and the results revealed that Varenicline plus nicotine replacement therapy was ranked first for sustained abstinence (Thomas et al., 2021). Although there were some differences in these two reviews, this also illustrated the potential smoking cessation benefits of the varenicline plus nicotine replacement therapy. As for Varenicline + Bupropion, the treatment was ranked second in all interventions, and the findings in this network meta-analysis showed that compared with Bupropion monotherapy, combination treatment with Varenicline and Bupropion could significantly improve the abstinence rate, but no statistical difference was found compared with Varenicline. However, a meta-analysis published in 2019 assessing the effects of the combination therapy of varenicline and bupropion in smoking cessation, the results showed the combination treatment was superior to Varenicline monotherapy (Zhong et al., 2019). This difference may be due to the incorporation of newer trials in our analysis, as well as potential heterogeneity (inconsistencies in populations and interventions) between studies.

In subgroup analyses, we investigated the effect of smoking cessation in 11 specific populations, in these populations, more trials reported smokers with chronic obstructive pulmonary disease, mental illness, cardiovascular disease, and alcohol dependence. For chronic obstructive pulmonary disease, our network meta-analysis showed that both Varenicline and Bupropion were superior to Placebo. Similarly, a review by Antoniu et al. (2021) showed the benefits of these two interventions for smoking cessation. However, in an earlier systematic review, the authors showed that Bupropion did not result significantly higher prolonged abstinence rates. In patients with mental illness, the results of this analysis showed that Varenicline, Bupropion, Nicotine replacement therapy, and Bupropion + Nicotine replacement therapy showed better withdrawal effects than placebo. A network meta-analysis by Roberts et al. (2016) also demonstrated that bupropion and Varenicline are effective and tolerable for smoking cessation in adults with severe mental illness. Interestingly, compared to the above studies, our study also found that Bupropion + Nicotine replacement therapy exhibited better withdrawal effects in psychiatric patients, which was significantly better than Bupropion, but no difference compared with Varenicline and Nicotine replacement therapy. Given that direct comparisons between these interventions are based on a limited number of studies, the quality of the evidence for this finding needs to be validated by more trials. For smokers with cardiovascular disease, our results found a significant withdrawal advantage for varenicline and bupropion, which is consistent with Karine’s findings (Suissa et al., 2017). For alcohol dependent smokers, more types of comparisons were reported, including seven pairs of direct comparisons and 21 pairs of indirect comparisons. The results showed that both Varenicline and Nicotine replacement therapy were superior to Placebo. Noteworthy, Guo et al. (2021) study also found that Varenicline can promote smoking cessation in alcohol-dependent people, while Naltrexone, Topiramate and Bupropion have no significant effect. In addition, withdrawal benefits of varenicline have been found in limited trials in patients with AIDS and substance use disorders, and are inconclusive in tuberculosis, asthma, post-traumatic stress disorder, and medical conditions. More high-quality studies are needed to validate this in the future.

Furthermore, assessing the quality of evidence for the findings is an important basis for practice. The first is that potential risk of bias of the included studies might be a critical determinant (Guyatt et al., 2011c). The risk of bias assessment revealed that over 80% of studies included in the review were rated as unclear risk of bias due to insufficient information and unclear reporting. These results directly lead to more trials being judged to be at high risk of bias, which can affect the reliability of the synthetic effects. Therefore, in the evaluation of the quality of evidence, it will lead to different degrees of downgrading, and while reducing the quality of the evidence, it may also affect the generalizability of the evidence (Guyatt et al., 2011c). In addition, other factors such as inconsistency and imprecision also contributed to weakening the quality of the evidence for the outcomes. For inconsistency, the heterogeneity on population, intervention, and confirmation of outcomes between included trials might be the main sources (Guyatt et al., 2011b). Moreover, there were significant differences in sample size between included trials, especially with small sample sizes in some trials, which could result in wide confidence intervals (imprecision) in the combined effect sizes (Guyatt et al., 2011a). These issues should receive further attention from future research and in health practice.

Based on this network meta-analysis, certain detailed improvements were found to be more promising for future research. Firstly, when conducting clinical trials, more details can be considered in the selection of smokers. For example, the physical condition of the smokers (with or without disease), the number of cigarettes smoked per day, and age may affect the effectiveness of the intervention. For example, some published studies showed that smokers with chronic obstructive pulmonary disease to have specific smoking characteristics that differentiated them from the rest of smokers and which complicated smoking cessation (Jiménez Ruiz et al., 2012; Zhang et al., 2022). In addition, smoker’s psychological conditions or preparation may influence withdrawal, Ussher et al. proposed measuring dependence and motivation of smokers to predict both short-term and medium-term outcomes of attempts to stop smoking in treatment-seeking smokers involved in a clinical trial, while Watson et al. assessed the effectiveness of anxiety and depression levels in predicting smoking cessation, these attempts have shown the importance of quitting intention and psychological state of smokers (Ussher et al., 2016; Watson et al., 2020). Therefore, researcher can minimize the differences between these factors in the participant selection process. Secondly, clarifying the details of intervention implementation plays an important role in practice. For example, a systematic review by Lindson et al. (2019) determined the effectiveness and safety of different forms, deliveries, doses, durations and schedules of nicotine replacement therapy, for achieving long-term smoking cessation, there was high-certainty evidence that using combination treatment vs. single-form nicotine replacement therapy, and high dose vs. low dose nicotine gum, could increase the chances of successfully stopping smoking. Overall, detailed reports on the dosage, frequency, and duration of specific pharmacological interventions should be fully considered. Finally, in terms of biochemical verification of tobacco abstinence, Benowitz et al. (2020) pointed out that biochemical verification could increase right and validity compared to self-reported smoking abstinence. However, for biomarkers such as exhaled carbon monoxide level, it needs to be assessed in the context of potential environmental exposures. As the degree of air pollution will affect the measurement of carbon monoxide level, it will lead to differences in the setting of parts per million value among researchers in different regions (Tual et al., 2010; Zhang et al., 2013; Maga et al., 2017), so future researchers could fully consider environmental factors when conducting biochemical verification.

The impact of some limitations on this study needs to be clarified. Although many clinical trials were included in this study, there is a possibility that some trials and interventions may be missed, considering the need for each intervention in the network meta-analysis to be interconnected. In addition, the included trials differed in follow-up time and types of outcome measures. In general, most trials reported a follow-up period of more than 6 months, but in some trials the follow-up period was insufficient. Collectively, these differences introduce potential bias to the level of evidence for the findings of this network meta-analysis. Moreover, from the network graph formed by all interventions, there are indirect comparisons between more interventions, which also means that direct comparisons between these interventions are lacking in clinical trials. Therefore, future trials of more intervention types are recommended to further clarify and validate existing findings.

## Conclusion

The results of this network meta-analysis showed that most pharmacological interventions demonstrated a benefit in smoking cessation compared with placebo, whether monotherapy or combination therapy. Among all monotherapies, Varenicline showed a higher level of evidence of smoking cessation. Furthermore, confirmed evidence suggested that some combination treatments, such as Varenicline plus Bupropion and Nicotine replacement therapy plus Mecamylamine have a higher probability of being the best smoking cessation interventions.

## Data Availability

The original contributions presented in the study are included in the article/Supplementary Material, further inquiries can be directed to the corresponding authors.
