# Supplementary material for: Pharmacological interventions on smoking cessation: A systematic review and network meta-analysis
Source: Front Pharmacol. 2022 Oct 24;13:1012433. doi: 10.3389/fphar.2022.1012433 (PMC9638092; doi:10.3389/fphar.2022.1012433)
Supplement: Supplementary file 1 [file Presentation1.pdf]

Supplementary Material

Supplementary Tables

Supplementary table 1. Search strategy (An example of PubMed database)

#1	"smok*"[All Fields] OR ("cigarett"[All Fields] OR "cigarette s"[All Fields] OR "cigaretts"[All Fields] OR "tobacco products"[MeSH Terms] OR ("tobacco"[All Fields] AND "products"[All Fields]) OR "tobacco products"[All Fields] OR "cigarette"[All Fields] OR "cigarettes"[All Fields]) OR ("tobacco"[MeSH Terms] OR "tobacco"[All Fields] OR "tobacco products"[MeSH Terms] OR ("tobacco"[All Fields] AND "products"[All Fields]) OR "tobacco products"[All Fields] OR "tobaccos"[All Fields] OR "tobacco s"[All Fields]) OR ("nicotine"[MeSH Terms] OR "nicotine"[All Fields] OR "nicotine s"[All Fields] OR "nicotines"[All Fields])
#2	"cessation"[All Fields] OR "cessations"[All Fields] OR "quit"[All Fields] OR ("quiting"[All Fields] OR "quits"[All Fields] OR "quitted"[All Fields] OR "quitting"[All Fields]) OR ("abstinence"[All Fields] OR "abstinences"[All Fields] OR "abstinent"[All Fields] OR "abstinents"[All Fields]) OR "stop"[All Fields] OR "stoping"[All Fields] OR ("withdraw"[All Fields] OR "withdrawal"[All Fields] OR "withdrawals"[All Fields] OR "withdrawing"[All Fields] OR "withdraws"[All Fields]) OR ("give"[All Fields] AND "up"[All Fields]) OR ("resist"[All Fields] OR "resistance"[All Fields] OR "resistances"[All Fields] OR "resistant"[All Fields] OR "resistants"[All Fields] OR "resisted"[All Fields] OR "resistence"[All Fields] OR "resistences"[All Fields] OR "resistent"[All Fields] OR "resistibility"[All Fields] OR "resisting"[All Fields] OR "resistive"[All Fields] OR "resistively"[All Fields] OR "resistivities"[All Fields] OR "resistivity"[All Fields] OR "resists"[All Fields]) OR ("withstand"[All Fields] OR "withstanding"[All Fields] OR "withstands"[All Fields])
#3	"randomized controlled trial"[Publication Type] OR "randomized controlled trials as topic"[MeSH Terms] OR "randomized controlled trial"[All Fields] OR "randomised controlled trial"[All Fields] OR ("controlled clinical trial"[Publication Type] OR "controlled clinical trials as topic"[MeSH Terms] OR "controlled clinical trials"[All Fields]) OR ("random allocation"[MeSH Terms] OR ("random"[All Fields] AND "allocation"[All Fields]) OR "random allocation"[All Fields] OR "random"[All Fields] OR "randomization"[All Fields] OR "randomized"[All Fields] OR "randomisation"[All Fields] OR "randomisations"[All Fields] OR "randomise"[All Fields] OR "randomised"[All Fields] OR "randomising"[All Fields] OR "randomizations"[All Fields] OR "randomize"[All Fields] OR "randomizes"[All Fields] OR "randomizing"[All Fields] OR "randomness"[All Fields] OR "randoms"[All Fields]) OR ("clinical trial"[Publication Type] OR "clinical trials as topic"[MeSH Terms] OR "clinical trials"[All Fields]) OR ("clinical trials as topic"[MeSH Terms] OR ("clinical"[All Fields] AND "trials"[All Fields] AND "topic"[All Fields]) OR "clinical trials as topic"[All Fields] OR "RCT"[All Fields]

Supplementary Table 2. Basic characteristics of included studies

Study	Country	Population	Intervention	Sample	Gender M/F	Age†	Cigarettes per day†	Implementation details (process, dosage or duration)	Follow -up	Outcome	Abstinence measurement
King 2022[1]	USA	Drink Heavily	Varenicline+NRT<br>Placebo+NRT	61<br>61	35/26<br>32/29	44.0±12.9<br>44.0±12.0	11.8±6.3<br>11.8±7.1	**Varenicline** 0.5 mg/day for 3 days, 0.5 mg twice daily for 4 days, then 1.0 mg twice daily through 12 weeks<br>**NRT** 14 mg /day for the first 6 weeks, then 7 mg/day for 4 weeks	—	①	Exhaled carbon monoxide level ⩽ 10 ppm
King 2006[2]	USA	Normal smokers	Naltrexone+NRT<br>Placebo+NRT	52<br>58	26/26<br>30/28	44.23±10.2<br>42.97±12.1	22.28±8.0<br>19.85±6.9	**Naltrexone** 25 mg/day for the first 3 days prior to the quit date, then 50 mg/day on the quit date and next 8 weeks<br>**NRT** beginning on the quit date, 21 mg/day for 2 weeks, 14 mg/day for 1 week, then 7 mg/day for 1 week	24wk	①	Exhaled carbon monoxide level ⩽ 10 ppm
Hughes 2003[3]	USA	History of Alcoholism	NRT<br>Placebo	61<br>54	44/17<br>34/20	43±8<br>43±9	30±11<br>29±11	**NRT** 21 mg/day for 6 weeks, 14 mg/day for 2 weeks, 7 mg/day for 2 weeks, and placebo for 2 weeks	26wk	①	Exhaled carbon monoxide level ⩽ 10 ppm
Hoogsteder 2014[4]	Netherlands	Normal smokers	NRT+Varenicline<br>Placebo+Varenicline	278<br>280	130/148<br>119/161	47.2 ± 8.4<br>47.6 ± 8.9	20 (15.3-24.4)<br>20 (16.8-25.0)	**NRT** injections with 400 µg NicVAX at 2, 2, 6, 10, 14 and 24weeks<br>**Varenicline** 0.5 mg/day for 3 days, 0.5 mg twice daily for 4 days, then 1.0 mg twice daily through 12 weeks	52wk	①②	Exhaled carbon monoxide level ⩽ 10 ppm
Nakamura 2017[5]	Japan	Normal smokers	Varenicline<br>Placebo	107<br>103	76/31<br>71/32	41.0±11.6<br>44.1±13.2	22.4±9.4<br>21.1±7.2	**Varenicline** 0.5 mg/day for 3 days, 0.5 mg twice daily for 4 days, then 1.0 mg twice daily through 24 weeks	52wk	①②	Exhaled carbon monoxide level ⩽ 10 ppm
Hughes 2011[6]	USA	Normal smokers	Varenicline<br>Placebo	107<br>111	65/42<br>63/48	45±13<br>45±13	19±9<br>17±7	**Varenicline** 0.5 mg/day for 3 days, 0.5 mg twice daily for 4 days, then 1.0 mg twice daily through 2-8 weeks	24wk	①②	Exhaled carbon monoxide level ⩽ 10 ppm
Pfeifer 2019[7]	Switzerland	Severe Alcohol Dependence	Varenicline<br>Placebo	15<br>13	13/2<br>11/2	45.73±9.27<br>44.15±6.84	26.2±13.4<br>25.9±10.2	**Varenicline** 0.5 mg/day for 3 days, 0.5 mg twice daily for 4 days, then 1.0 mg twice daily through 12 weeks.	—	①	Exhaled carbon monoxide level
Gray 2014[8]	USA	Normal smokers	Varenicline<br>NRT	67<br>73	0/67<br>0/73	30.7±7.9<br>33.0±7.4	16.9±7.7<br>17.0±6.5	**Varenicline** 0.5 mg/day on days -7 through -5, 0.5 mg twice daily on days -4 through - 1, then 1.0 mg twice daily through 4 weeks<br>**NRT** 21 mg/day for 4 weeks	—	①	Exhaled carbon monoxide level ⩽ 10 ppm
Bohadana 2019[9]	Israel	Normal smokers	Varenicline<br>Placebo	121<br>121	88/33<br>89/32	48.2±12.8<br>47.9±14.4	24.5±13.4<br>24.9±13.4	**Varenicline** 0.5 mg/day for 3 days and 0.5 mg twice daily for 4 days, then 1 mg twice daily through 6 weeks. Following preloading, 1 mg twice daily for 12 weeks	30wk	①②	Exhaled carbon monoxide level ⩽5 ppm<br>Urine cotinine concentration ⩽1 mg/mL

Study	Country	Population	Intervention	Sample	Gender M/F	Age†	Cigarettes per day†	Implementation details (process, dosage or duration)	Follow -up	Outcome	Abstinence measurement
Sun 2009[10]	China	Normal smokers	NRT<br>Placebo	101<br>110	94/7<br>106/4	43.22±11.42<br>39.63±11.30	23.98±10.41<br>23.55±9.98	**NRT** Highly dependent smokers,4mg per hour, up to a maximum of 40mg/day; Less dependent smokers 2mg per hour, up to a maximum of 40mg/day, through 11 weeks	—	①	Exhaled carbon monoxide level Urine cotinine concentration
Gray 2020[11]	USA	Normal smokers	Varenicline<br>Placebo	109<br>100	70/38<br>63/36	16.0±2.0<br>15.8±1.8	12.8±7.5<br>12.0±6.0	**Varenicline** 1 mg twice daily, or 0.5 mg twice daily if bodyweight ≤55 kg through 12 weeks	52wk	①②	Urine cotinine testing(threshold 200 ng/mL)
Kalman 2011 [12]	USA	alcohol dependence	Bupropion+NRT<br>Placebo+NRT	73<br>70	119/24	47.8±10.5<br>49.2±7.5	20.1±9.1<br>21.7±12.8	**Bupropion** 150 mg/day for 3 days, then 150 mg twice daily through 8 weeks **NRT** starting on quit day, 21 mg/day for 4 weeks, 14 mg/day for 2 weeks, then 7 mg/day for 1 week	24wk	①②	Exhaled carbon monoxide level ≤8 ppm Salivary cotinine levels ≤ 15 ng/ml
Jamerson 2001[13]	USA	Normal smokers	Bupropion<br>NRT<br>Bupropion+NRT<br>Placebo	244<br>244<br>245<br>160	464<br>429	44 (42-44)	27 (25-28)	**Bupropion** 150 mg/day for the first 3 days, then 150 mg twice daily through days 63 **NRT** 21 mg/day for weeks 2 to 7, 14 mg and then 7 mg for the final 2 weeks	48wk	①②	Exhaled carbon monoxide level ≤10 ppm
Scherphof 2014[14]	Netherlands	Normal smokers	NRT<br>Placebo	135<br>122	—	16.7±1.13	≥7 cigarettes	**NRT** for 6-9 weeks (depending on the number of cigarettes participants smoked at first, concentrations of the patches were based on the manufacturer's instructions leaflet of the nicotine patches)	48wk	①	Salivary cotinine levels
Benjamin 2010[15]	USA	Normal smokers	Naltrexone+NRT<br>Placebo+NRT	87<br>85	25/62<br>24/61	43.2±10.0<br>45.5±11.25	22.1±10.27<br>22.2±8.62	**Naltrexone** 12.5 mg for 1 day, then 25 mg for 27 weeks (1 week pre-and 26 weeks post-quit) **NRT** 21 mg/day for 6 weeks, then 14 mg for 2 weeks (beginning on their quit date)	26wk	①②	Exhaled carbon monoxide level ≤10 ppm
Nides 2021[16]	USA	Normal smokers	Cytisinicline<br>Placebo	51<br>51	23/28<br>20/31	49.8±11.46<br>49.0±13.94	18.5±5.96<br>18.2±6.07	**Cytisinicline** 1.5 mg and 3 mg, downward titration or 3 times daily through 8weeks	—	①②	Exhaled carbon monoxide level ≤10 ppm
Dalsgarð 2003[17]	Denmark	Normal smokers	Bupropion<br>Placebo	221<br>114	56/165<br>29/85	42.5±9.9<br>44.3±9.4	18.9±5.5<br>19.3±6.4	**Bupropion** 150 mg/day for the first 3 days, then 150 mg twice daily through 7 weeks	26wk	①	Exhaled carbon monoxide level ≤10 ppm
Tønnesen 2003[18]	Denmark	Normal smokers	Bupropion<br>Placebo	527<br>180	48/479<br>50/130	42.4±9.8<br>41.9±9.5	22.4±8.2<br>23.5±9.8	**Bupropion** 150 mg/day for the first 3 days, then 150 mg twice daily through 7 weeks	52wk	①②	Exhaled carbon monoxide level ≤10 ppm
Ebbert 2014[19]	USA	Normal smokers	Varenicline+Bupropion<br>Varenicline	249<br>257	113/136<br>126/131	42.2±12.2<br>41.9±12.7	19.5±7.3<br>19.7±7.9	**Varenicline** 0.5 mg/day for 3 days, 0.5 mg twice daily for 4 days, then 1.0 mg twice daily through 12 weeks **Bupropion** 150 mg/day for 3 days, then 150 mg twice daily through 12 weeks	52wk	①②	Exhaled carbon monoxide level ≤8 ppm

Study	Country	Population	Intervention	Sample	Gender M/F	Age†	Cigarettes per day†	Implementation details (process, dosage or duration)	Follow -up	Outcome	Abstinence measurement
Covey 1999[20]	USA	Normal smokers	Naltrexone<br>Placebo	30<br>38	10/20<br>15/23	33.8±8.2<br>39.7±8.0	30.3±10.1<br>34.3±11.9	**Naltrexone** 25 mg at least three days prior to quit day, then 50 mg through 4 weeks. If the 50 mg dose had been well tolerated up to that time, dosing was increased to 75 mg. At the Week 4 (end-of-treatment) visit, the patient received instructions for tapering naltrexone (25 mg every 3 days)	24wk	①	Plasma cotinine < 15 ng/mL
Oncken 2019[21]	USA	Pregnant Smokers	NRT<br>Placebo	70<br>67	0/70<br>0/67	26.97±5.45<br>28.24±6.30	8.66±4.71<br>8.04±4.59	**NRT** smokers were instructed to begin by using 1–4 cartridge inhalers per day through 6 weeks, followed by a 6 weeks taper period, each cartridge inhaler estimated to release 4 mg of nicotine	32wk	①②	Exhaled carbon monoxide level ≤4 ppm
Oncken 2008[22]	USA	Pregnant Smokers	NRT<br>Placebo	100<br>94	0/100<br>0/94	25.5±6.8<br>24.7±5.4	10.2±6.6<br>8.7±5.7	**NRT** Subjects were instructed to not chew more than 20 pieces per day, through 6 weeks, followed by a 6 weeks taper period. The gum contains nicotine 2 mg	34wk	①②	Urinary cotinine concentration
Caldwell 2016[23]	New Zealand	Normal smokers	NRT<br>Placebo	246<br>256	124/122<br>124/132	45.2±11.4<br>45.0±11.0	19.0±6.7<br>19.2±6.5	**NRT (Patch)** 21 mg/day for 18 weeks, 14 mg/day for 2 weeks, 7 mg/day for 2 weeks<br>**(Inhaler)** Subjects started with 100 µg/puff and moved on to 200 µg/puff once they had developed tolerance to the upper airway effects of the lower dose	24wk	①②	Exhaled carbon monoxide level ≤10 ppm
Bohadana 2000[24]	France	Normal smokers	NRT<br>Placebo	200<br>200	99/101<br>97/103	37.1±8.1<br>37.4±8.8	26.1±11.0<br>23.5±8.6	**NRT** received the nicotine inhaler plus nicotine patch (delivering 15 mg of nicotine per 16 hours) for 6 weeks, then inhaler plus placebo patch for 6 weeks, then inhaler alone for 14 weeks	48wk	①	Exhaled carbon monoxide level ≤10 ppm
Hjalmerson 1997[25]	Sweden	Normal smokers	NRT<br>Placebo	123<br>124	47/76<br>42/82	48.0+10.6<br>47.0±9.5	21.7+8.1<br>21.0+7.8	**NRT** minimum dosage of 4 inhalers per day. After 12 weeks participants were advised to decrease their inhaler use	48wk	①	Exhaled carbon monoxide level ≤10 ppm<br>Saliva cotinine concentrations
Hurt 1994[26]	USA	Normal smokers	NRT<br>Placebo	120<br>120	62/58<br>67/53	42.8+11.1<br>43.6±10.6	28.8+9.4<br>30.6±9.4	**NRT** 22 mg/day for 8 weeks	48wk	①②	Exhaled carbon monoxide level ≤8 ppm
WGSTNP 1994[27]	USA	Coronary artery disease	NRT<br>Placebo	77<br>79	62/15<br>62/17	56.0±7.5<br>55.9±8.1	32.3±9.4<br>31.1±11.0	**NRT** 14 mg/day for 1 week, patients who had smoked more than seven cigarettes were able to increase to 21 mg/d, other patients remain unchanged, all through 5 weeks	—	①	Exhaled carbon monoxide level ≤8 ppm
Etter 2001[28]	Switzerland	Normal smokers	NRT<br>Placebo	265<br>269	143/122<br>132/137	Mean: 43.2<br>Mean: 41.7	29.8±10.3<br>29.4±9.4	**NRT** 25 mg and delivers 15 mg nicotine over 16 hours, a nicotine gum contains 4 mg and delivers 2 mg nicotine, and a nicotine inhaler (a plug contains 10 mg	24wk	①②	Exhaled carbon monoxide level

Study	Country	Population	Intervention	Sample	Gender M/F	Age†	Cigarettes per day†	Implementation details (process, dosage or duration)	Follow -up	Outcome	Abstinence measurement
								and delivers 5 mg nicotine) all through 6 months			
Rose 1998[29]	USA	Normal smokers	NRT+Mecamylamine<br>NRT<br>Mecamylamine<br>Placebo	20<br>20<br>20<br>20	11/9<br>10/10<br>11/9<br>7/13	41.0±7.2<br>39.4±9.9<br>40.1±6.9<br>41.4±8.1	25.4±9.0<br>35.0±11.2<br>29.6±7.7<br>29.7±11.1	**Mecamylamine** four weeks before quitting, 10 mg/day, then continuing for 6 weeks after the target quit-smoking date<br>**NRT** 4 weeks before quitting, 21 mg/day, then continuing for 6 weeks after the target quit-smoking date, then 14 mg day for 1 week, then 7mg/day for 1 week	24wk	①②	Exhaled carbon monoxide level ≤8 ppm
Aveyard 2008[30]	Britain	Normal smokers	Nortriptyline<br>Placebo	445<br>456	239/206<br>243/213	43.2±11.5<br>44.0±12.4	21.4±8.1<br>21.4±8.1	**Nortriptyline** 1 to 2 weeks before quit day participants used 25 mg of either drug for 3 days, 50 mg for 4 days, and 75 mg thereafter, a dose found effective in previous trials. The participants took the maximum dose for 6 weeks and then reduced the dose over 1 week	48wk	①②	Exhaled carbon monoxide level ≤10 ppm<br>Salivary cotinine concentration ≤15 ng/ml
Wong 2013[31]	Canada	Normal smokers	Varenicline<br>Placebo	151<br>135	83/68<br>68/67	51.9±11.8<br>53.3±11.4	17.8±8.2<br>17.0±7.5	**Varenicline** 0.5 mg/day for 3 days, 0.5 mg twice daily for 4 days, then 1.0 mg twice daily for 8-12 weeks	48wk	①②	Exhaled carbon monoxide level<br>Urinary cotinine concentration
Ebbert 2011[32]	USA	Normal smokers	Varenicline<br>Placebo	38<br>38	38/0<br>38/0	40.7±10.1<br>41.0±12.4	4.0±3.5<br>3.2±2.0	**Varenicline** 0.5 mg twice daily for 4 days, then 1.0 mg twice daily through 12 weeks	24wk	①②	Salivary cotinine concentration ≤50ng/ml
Cooper 2004[33]	USA	Normal smokers	NRT<br>Placebo	146<br>148	0/146<br>0/148	38.4±10.8<br>39.0±10.2	≥10 cigarettes	**NRT** beginning at the quit date, 10 to 12 pieces per day for 8weeks, each piece of nicotine gum was 2 mg	48wk	①	Exhaled carbon monoxide level ≤10 ppm
George 2002[34]	USA	Schizophrenia	Bupropion<br>Placebo	16<br>16	10/6<br>8/8	45.4±11.9<br>40.9±9.4	25.0±11.5<br>23.3±9.5	**Bupropion** began the second week, 150 mg/day for 3 days, then 150 mg twice daily through 10 weeks.	24wk	①②	Exhaled carbon monoxide level ≤10 ppm
Etter 2006[35]	Switzerland	Normal smokers	NRT<br>Placebo	265<br>269	143/122<br>132/137	Mean: 43.2<br>Mean: 41.7	29.8±10.3<br>29.4±9.4	**NRT** choose among a nicotine transdermal patch (contains 25 mg and delivers 15 mg nicotine over 16 hours), a nicotine gum (contains 4 mg and delivers 2 mg nicotine), and a nicotine inhaler (a plug contains 10 mg and delivers 5 mg nicotine), or a combination of these, all through 6 months	5 years	①	Exhaled carbon monoxide level

Study	Country	Population	Intervention	Sample	Gender M/F	Age†	Cigarettes per day†	Implementation details (process, dosage or duration)	Follow -up	Outcome	Abstinence measurement
Etter 2004[36]	Switzerland	Normal smokers	NRT<br>Placebo	265<br>269	143/122<br>132/137	Mean: 43.2<br>Mean: 41.7	29.8±10.3<br>29.4±9.4	**NRT** choose among a nicotine transdermal patch (contains 25 mg and delivers 15 mg nicotine over 16 hours), a nicotine gum (contains 4 mg and delivers 2 mg nicotine), and a nicotine inhaler (a plug contains 10 mg and delivers 5 mg nicotine), or a combination of these, all through 6 months	2 years	①②	Exhaled carbon monoxide level
George 2006[37]	USA	Normal smokers	Selegiline<br>Placebo	20<br>20	8/12<br>7/13	49.7±7.0<br>48.3±10.3	23.0±10.0<br>22.4±8.1	**Selegiline** 5 mg/day for the first week, then 5 mg twice daily for 7weeks, then 5 mg/day for 1 week	24wk	①②	Exhaled carbon monoxide level ≤10 ppm
Hertzberg 2001[38]	USA	Chronic Posttraumatic Stress Disorder	Bupropion<br>Placebo	10<br>5	10/0<br>5/0	50（47-58)	33（15-99)	**Bupropion** 150 mg/day for 3 or 4 days and increased to a final dose of 150 mg twice daily, all through 12 weeks	24wk	①	Exhaled carbon monoxide level
Killen 2004[39]	USA	Adolescent Smokers	NRT+Bupropion<br>NRT	103<br>108	71/32<br>74/34	14.23±9.34<br>14.14±9.56	15.12±5.33<br>15.65±6.40	**NRT** 21 mg patches during weeks 1-4, 14 mg patches in weeks 5-6, and 7 mg patches in weeks 7-8. However, if participants smoked between 10 and 15 cigarettes per day, they wore 14 mg patches in weeks 1-6, and 7 mg patches in weeks 7-8<br>**Bupropion** 150 mg/day through 9 weeks	26wk	②	Exhaled carbon monoxide level ≤9 ppm<br>Saliva cotinine level < 20 ng/ml
Croghan 2007[40]	USA	Normal smokers	NRT<br>Bupropion<br>NRT+Bupropion	566<br>567<br>567	228/338<br>229/338<br>227/340	42.6±11.65<br>42.7±11.35<br>43.1±11.56	23.0±8.82<br>23.5±10.22<br>23.4±9.47	**NRT** up to 16 cartridges per day for 12 weeks<br>**Bupropion** 300 mg/day for 12 weeks	36wk	①②	Exhaled carbon monoxide level ≤8 ppm
Tsukahara 2015[41]	Japan	Adult smokers	Varenicline<br>NRT	14<br>14	12/2<br>11/3	45.4±12.98<br>46.8±10.71	27.9±10.87<br>25.4±7.96	**Varenicline** 0.5–2 mg/day for 3 days, 0.5 mg/day for days 4–7, 1 mg/day for days 8–84<br>**NRT** 52.5–17.5 mg/day: 52.5 mg for 4 weeks, 35 mg for 2 weeks, then 17.5 mg for 2 weeks	24wk	①	Exhaled carbon monoxide level ≤8 ppm
Schnoll 2019[42]	USA	Cancer patients	Varenicline<br>Placebo	105<br>102	59/46<br>43/59	60.0±9.5<br>58.0±9.4	13.2±8.6<br>13.5±7.9	**Varenicline** Day 1-Day 3 (0.5mg once daily); Day 4– 7 (0.5mg twice daily), and Day 8-Day 84 (1.0mg twice daily)	52wk	①②	Exhaled carbon monoxide level ≤10 ppm
Piper 2009[43]	USA	Normal smokers	Placebo<br>Bupropion<br>Lozenge<br>Patch<br>Bupropion+ Lozenge<br>Patch+ Lozenge	189<br>264<br>260<br>262<br>262<br>267	78/111<br>110/154<br>109/151<br>109/153<br>108/154<br>114/153	43.1±11.4<br>43.9±11.7<br>45.3±10.4<br>44.9±11.6<br>45.3±10.4<br>44.2±11.1	21.0±8.3<br>21.4±8.2<br>21.6±9.1<br>21.4±9.2<br>21.0±8.5<br>21.93±9.6	**Bupropion** (150 mg, bid for 9 weeks total), **Lozenge**（2 or 4 mg, for 12 weeks post-quit), **Patch** (24-hour patch, 21, 14, and 7mg, titrated down over 8 weeks post-quit), **Patch** (24-hour patch, 21, 14, and 7mg, titrated down over 8 weeks post-quit) **+ Lozenge** (2 or 4 mg, for 12 weeks post-quit) combination therapy, **Bupropion** (150 mg, bid for 9 weeks total）**+ Lozenge** (2 or 4 mg, for 12 weeks post-quit) combination therapy	24wk	②	Exhaled carbon monoxide level ≤10 ppm

Study	Country	Population	Intervention	Sample	Gender M/F	Age†	Cigarettes per day†	Implementation details (process, dosage or duration)	Follow -up	Outcome	Abstinence measurement
Rennard 2011[44]	USA	Normal smokers	Varenicline<br>Placebo	493<br>166	296/197<br>99/67	43.9±12.5<br>43.2±12.2	21.3(10-70)<br>21.5(10-65)	**Varenicline** 0.5 mg once daily for 3 days, then 0.5 mg twice daily for 4 days, through 12week	24wk	①②	Exhaled carbon monoxide level ≤10 ppm
Wong 1999[45]	USA	Normal smokers	Naltrexone<br>Naltrexone+NRT<br>Placebo<br>NRT	23<br>26<br>26<br>25	47/53	42.1±10.9	27.8± 11.8	**Naltrexone** 50 mg once daily<br>**NRT** 21 mg/24-hour for the first 8 weeks and 14 mg/24-hour for the remaining 4 weeks	24wk	①②	Exhaled carbon monoxide level ≤8 ppm
Stapleton 2012[46]	UK	Normal smokers	NRT<br>Bupropion<br>NRT+Bupropion	418<br>409<br>244	200/218<br>186/223<br>115/129	40.8±11.9<br>40.7±11.7<br>41.2±12.1	20.7±8.7<br>19.8±8.1<br>20.3±9.7	**Bupropion** 150 mg for the first 6 days and 300 mg for the remain der of the 8-week course	24wk	①	Exhaled carbon monoxide level ≤10 ppm
Evins 2007[47]	USA	Schizophrenia	Bupropion+NRT<br>NRT	25<br>26	—	44.8±9.2<br>43.6±10.9	28.1±14.3<br>24.7±10.1	**Bupropion** 150 mg once daily for 7 days, then twice daily for 11 weeks	48wk	①②	Exhaled carbon monoxide level ≤8 ppm
Hays 2009[48]	USA	Alcohol abuse or dependence	Bupropion<br>Placebo	56<br>54	44/12<br>42/12	43.9±10.7<br>44.0±9.3	28.3±10.9<br>27.8±11.0	**Bupropion** 150 mg/day for 3 days followed by 150 mg twice daily	76wk	②	Exhaled carbon monoxide level ≤8 ppm
Franks 1989[49]	USA	Normal smokers	Clonidine<br>Placebo	92<br>93	41/51<br>41/52	39.1±9.4<br>37.9±8.9	31.6±11.2<br>31.6±11.8	**Clonidine hydrochloride** while continuing to smoke, participants took 0.1-mg tablets of clonidine in a dosage that increased every other day as tolerated to 0.2 mg twice a day	—	①	Exhaled carbon monoxide level ≤12 ppm
Elbert 2007[50]	USA	Normal smokers	NRT+ Mecamylamine<br>NRT	180<br>180	86/94<br>88/92	41.8(20-69)<br>41.2(19-70)	Mean: 28.8<br>Mean: 28.8	**21 mg NRT** + 6 mg **mecamylamine**, 21 mg **nicotine** + 3 mg **mecamylamine**, 21 mg **nicotine**, through 8weeks	—	①	Exhaled carbon monoxide level ＜10 ppm
Williams Jill M 2012[51]	USA	Schizophrenia	Varenicline<br>Placebo	84<br>43	65/19<br>33/10	40.2±11.9<br>43.0±10.2	23.5（15-50)<br>22.3（15-50）	**Varenicline** 0.5mg on day 1-3 of week 1, followed by two 0.5 mg tablet/d (1 morning,1 evening) for the next 4 days, through 12weeks	24wk	②	Exhaled carbon monoxide level ≤10 ppm
Paul M. Cinciripini 2018[52]	USA	Normal smokers	Varenicline+Bupropion<br>Varenicline<br>Placebo	163<br>166<br>56	95/68<br>98/68<br>32/24	49.36±9.38<br>48.75±10.89<br>48.48±10.33	19.64±9.49<br>19.02±9.49<br>19.71±11.20	**Varenicline** (0.5 mg/day for days 1–3, 0.5 mg bid for days 4–7, and 1 mg bid thereafter)<br>**Bupropion** (150 mg/day for days 1–3, 150 mg bid thereafter), through 12weeks	48wk	①②	Exhaled carbon monoxide level ＜ 4 ppm
Zawertailo Laurie 2020[53]	Canada	Alcohol-Dependent Smokers	Varenicline<br>Placebo	16<br>15	11/5<br>11/3	46.1±11.8<br>40.6±9.5	—	**Varenicline** 0.5 mg once per day for the first 3 days, 0.5 mg 2 times a day for the next 4 days, then to 1 mg 2 times a day for the next 11 weeks	26wk	②	Exhaled carbon monoxide level ≤10 ppm
James 2010[54]	USA	Normal smokers	Varenicline<br>Placebo	13<br>18	11/2<br>14/4	36.5±12<br>34.4±12	Mean:18.2<br>Mean:19.1	**Varenicline** 2 mg/ day, 5 mg/day during days 1–3 of Week 2, and 5 mg twice per day during days 4–7, through 12weeks	—	①	Expired carbon monoxide level < 8 ppm

Study	Country	Population	Intervention	Sample	Gender M/F	Age†	Cigarettes per day†	Implementation details (process, dosage or duration)	Follow -up	Outcome	Abstinence measurement
Scherphof 2014[55]	Netherlands	Adolescents	NRT<br>Placebo	135<br>122	55/80<br>66/56	16.56±1.11<br>16.70±1.16	≥7 cigarettes	**NRT** adolescents who were smoking more than 20 cigarettes a day received a higher patch dose and continued use for 9 weeks (**patches** 3 weeks 21 mg/day, 3 weeks 14 mg/day and 3 weeks 7 mg/day), whereas adolescents smoking less than 20 cigarettes a day used a lower dose for a period of 6 weeks (**patches** 3 weeks 14 mg/day and 3 weeks 7 mg/day)	—	①	Self reported
Sarah 2018[56]	Canada、USA	Acute coronary syndrome	Varenicline<br>Placebo	151<br>151	112/39<br>115/36	54.7±8.4<br>55.3±10.3	21.9±10.9<br>21.0±10.3	**Varenicline** (0.5 mg daily for 3 days, then 0.5 mg twice daily for 4 days, followed by 1.0 mg for 11 weeks)	52wk	①	Exhaled carbon monoxide level ⩽10 ppm
Ned 2009[57]	USA	Alcohol abuse or dependence	NRT<br>Placebo	45<br>51	32/13<br>36/15	45.1±10.2<br>44.8±10.1	—	**NRT** 21 mg nicotine patch daily for 8 weeks, followed by one 14 mg patch daily for 2 weeks, then followed by one 7 mg patch daily for 2 weeks, for a total of 12 weeks of nicotine patch therapy	48wk	①	Exhaled carbon monoxide level ＜ 10 ppm
Tonstad 2006[58]	Norway	Normal smokers	Varenicline<br>Bupropion<br>placebo	352<br>329<br>344	—	—	—	**Varenicline** 1 mg twice daily, **Bupropion** 150 mg twice daily for 12 weeks	52wk	①	Exhaled carbon monoxide level ⩽10 ppm
Eva 2009[59]	Czech Republic	Normal smokers	NRT<br>Placebo	209<br>105	89/120<br>42/63	46.1±10.5<br>46.6±10.0	25.7±9.8<br>25.2±8.2	**NRT** (inhaler 10 mg or placebo) or (gum 4 mg or placebo), through 24weeks	48wk	①	Exhaled carbon monoxide level ＜ 10 ppm
Nides 2006[60]	USA	Normal smokers	Varenicline<br>Bupropion<br>Placebo	126<br>126<br>123	63/63<br>57/69<br>64/59	41.9±10.6<br>40.5±10.8<br>41.6±10.4	20.3±7.7<br>19.5±6.9<br>21.5±8	**Varenicline** (0.3 mg once daily, 1.0 mg once daily, or 1.0 mg twice daily), **Bupropion** (150 mg twice daily), through 12weeks	52wk	①	Exhaled carbon monoxide level ⩽10 ppm
Duška 2003[61]	Croatia	Health Care Workers	NRT<br>Placebo	56<br>56	19/37<br>19/37	34.4±4.7<br>33.8±4.4	24.1±5.8<br>22.5±5.7	**NRT** patches are generally applied over 24 h and then substituted, delivering either 20-25 mg of nicotine per 24 h (30 cm^2 of active area), 13-15 mg/24 h (20 cm^2), or 7-8 mg/24 h (10 cm^2)	5 years	①	Exhaled carbon monoxide level ＜ 11 ppm
Sue 2014[62]	UK	Normal smokers	NRT<br>Placebo	521<br>529	0/521<br>0/529	26.4 ±6.2<br>26.2 ±6.1	13 (10–20)<br>15 (10–20)	**NRT** 15 mg per 16 hours	2 years	①	Exhaled carbon monoxide level ⩽8 ppm
Fagerstrom 2010[63]	Sweden	Normal smokers	Varenicline<br>Placebo	213<br>218	189/24<br>196/22	43.9±12.0<br>43.9±12.0	15.4±5.8<br>15.9±7.7	**Varenicline** 0.5 mg once daily for three days, then0.5 mg twice daily for four days), followed by 1 mg twice daily, through week 12	26wk	①	Exhaled carbon monoxide level ⩽10 ppm
Costa 2002[64]	Brazil	Normal smokers	Nortriptyline<br>Placebo	68<br>76	—	18 to 65 years	＞15 cigarettes	**Nortriptyline** dose was increased weekly from one tablet (25 mg), reaching a dose of three tablets (75 mg) until the end of the study (42 days)	—	①	Exhaled carbon monoxide level
Paul 2016[65]	USA	Posttraumatic stress disorder	NRT<br>Placebo	32<br>31	14/18<br>15/16	42.3±10.7<br>42.8±7.9	—	**NRT** starting with 21 mg/24 h nicotine patches, through 6 weeks	—	①	Exhaled carbon monoxide level ⩽10 ppm

Study	Country	Population	Intervention	Sample	Gender M/F	Age†	Cigarettes per day†	Implementation details (process, dosage or duration)	Follow -up	Outcome	Abstinence measurement
Hays 2001[66]	USA	Normal smokers	Bupropion<br>Placebo	214<br>215	97/117<br>112/103	47.0±9.7<br>45.4±9.2	27.4±10.6<br>26.2±9.6	**Bupropion** 300 mg/d, for 7 weeks	78wk	①	Exhaled carbon monoxide level ≤10 ppm
Steinberg 2011[67]	USA	Normal smokers	Varenicline<br>Placebo	40<br>39	24/16<br>23/16	51 (22-78)	≥10 cigarettes	**Varenicline** 0.5 mg daily for 3 days, then 0.5 mg twice daily for 4 days, then 1 mg twice daily, through 12 weeks	24wk	①②	Exhaled carbon monoxide level ≤8 ppm
Oncken 2013[68]	USA	Normal smokers	Placebo<br>Topiramate<br>NRT	19<br>19<br>19	8/11<br>9/10<br>6/13	45.4±7.6<br>46.1±11.1<br>50.1±9.6	22.1±6.9<br>21.1±6.1<br>20.9±7.2	**Topiramate** titrated up over 5 weeks (25mg/day for 1 week, 25mg twice daily for 1 week, 50mg twice daily for 1 week, 75mg twice daily for 1 week, and 100mg twice daily for 5 weeks)	—	①②	Exhaled carbon monoxide level ≤10 ppm
Prochazka 1992[69]	USA	Normal smokers	Clonidine<br>Placebo	106<br>107	53/53<br>50/57	44.4 (23-73)<br>43.9 (26-67)	—	**Clonidine** transdermally at a rate of 0.1 mg and 0.2 mg per day	—	①②	Exhaled carbon monoxide level≤ 8 ppm<br>Salivary cotinine ≤20 ng/mL
Stapleton 2011[70]	UK	Normal smokers	NRT<br>Placebo	506<br>255	229/277<br>96/159	40.1±10.5<br>40.9±10.0	22.7±8.2<br>24.0±8.1	**NRT** maximum of 3 doses/hour or 32 doses/day (They were advised to use the spray whenever they felt an urge to smoke up)	—	②	Exhaled carbon monoxide level
Steinberg 2009[71]	USA	Medically Ill Smokers	NRT+Bupropion<br>NRT	63<br>64	23/40<br>22/42	7±11.1<br>26±41.2<br>24±38.1<br>6±9.5	21 ±33.3<br>42±66.7	**NRT** patches (21mg/d for 6 weeks, followed by 14 mg/d for 2 weeks and then 7 mg/d for 2 weeks), nicotine patch starting at 21 mg/d, a nicotine oral inhaler (to be used as needed), and sustained release **Bupropion**, 150 mg/d.	26wk	②	Exhaled carbon monoxide level ≤8 ppm
Jamshid 2003[72]	Iran	Normal smokers	NRT<br>Clonidine<br>Naltrexone	57<br>57<br>57	57/0<br>57/0<br>57/0	17~64 years	≥10 cigarettes	**NRT** gum 2 mg pieces for the first 6 weeks, 2 mg every 2 to 4 h for the next 3 weeks, and 2 mg every 4 to 8 h for the remaining 15 weeks), **Naltrexone** (50 mg), and **Clonidine** (0.4 mg)	24wk	①	Exhaled carbon monoxide level
Elin 2006[73]	UK	Normal smokers	NRT<br>Placebo	49<br>49	18/31<br>22/27	14.9<br>14.7	—	**NRT** 15 mg/10 mg/5 mg for two weeks each for a maximum of 6 weeks	—	②	Carbon monoxide validation＜5 ppm
Bankole 2005[74]	USA	Alcohol-Dependent Smokers	Topiramate<br>Placebo	45<br>49	34/11<br>36/13	21~65 years	—	**Topiramate** (escalating dose from 25 to 300 mg/d)	—	②	Serum cotinine level cutoff level was 28 ng/mL
Hajek 2011[75]	UK	Normal smokers	Varenicline<br>Placebo	53<br>48	34/19<br>31/17	45.9±11.4<br>45.3±10.9	19.5±9.8<br>18.2±8.9	**1 mg of varenicline** tartrate per day for their first week of varenicline use (study week 1 for the varenicline arm and study week 4 for the placebo arm) and 2 mg/d from the second week onward, through 12 weeks	—	②	Exhaled carbon monoxide level

Study	Country	Population	Intervention	Sample	Gender M/F	Age†	Cigarettes per day†	Implementation details (process, dosage or duration)	Follow -up	Outcome	Abstinence measurement
Piper 2008[76]	USA	Normal smokers	Bupropion+NRT<br>Bupropion<br>Placebo	228<br>224<br>156	101/127<br>89/135<br>66/90	—	≥10 cigarettes	**Bupropion** sustained release (150mg, b.i.d.) ＋ active 4mg **NRT gum**, **Bupropion** sustained release ＋ **placebo**, or **placebo bupropion** sustained release＋ **placebo gum**, through 24weeks	48wk	②	Exhaled carbon monoxide level
Tuisku 2016[77]	Finland	Normal smokers	Placebo<br>NRT<br>Varenicline	86<br>94<br>60	42/44<br>45/49<br>30/30	20 (18.0-23.3)<br>21 (19.0-23.0）<br>21 (19.0-23.8）	10 (8.0-15.0）<br>10 (7.0-14.3）<br>18 (15.0-20.0）	**NRT** 10 mg/16 h for 8 weeks, **varenicline** for 12 weeks	52wk	②	Saliva cotinine level ≤10 ng/ml
Evan 2018[78]	USA	Normal smokers	Varenicline<br>Placebo	37<br>25	31/6<br>20/5	32.2±6.0<br>32.6±9.3	9.3±5.7<br>7.4±3.0	**Varenicline** was titrated to 2 mg a day (1 mg, BID) over days 1–8, through 4 weeks	—	①	Exhaled carbon monoxide level<br>Urine cotinine level≤ 100ng/ml
Stein 2013[79]	USA	Methadone-maintained smokers	NRT<br>Varenicline<br>Placebo	133<br>137<br>45	65/68<br>63/74<br>28/17	40.3±9.3<br>39.2±9.7<br>40.6±10.6	19.1±7.8<br>19.5±8.5<br>21.1±10.4	**Varenicline** 0.5mg for 3 days, then two 0.5 mg pills for 4 days, 1 mg twice daily after 1 week<br>**NRT**, given at 4week intervals for up to 24 weeks of therapy (For participants who smoked >30 cigarettes per day the treatment began at 42 mg, and for participants smoking <30 cigarettes per day the treatment began at 21 mg)	24wk	②	Exhaled carbon monoxide level
Ebbert 2016[80]	USA	Light Smokers	Varenicline<br>Placebo	45<br>48	23/22<br>14/34	37.1±11.7<br>37.2±11.3	7.9±1.5<br>7.5±1.5	**Varenicline** 0.5mg once daily for 3 days, then increased to 0.5mg twice daily for days 4–7 to a target dose of 1mg twice daily for a total of 12 weeks of treatment	24wk	②	Exhaled carbon monoxide level ≤8 ppm
Antoniu 2011[81]	Romania	chronic obstructive pulmonary disease	Varenicline<br>Placebo	248<br>251	—	Mean: 57	24-25 cigarettes	**Varenicline** (0.5 mg once daily for 3 days, 0.5 mg b.i.d. for 4 days, then 1 mg b.i.d. for a total of 12 weeks) or matching placebo for 12 weeks and underwent follow up for 40 weeks	52wk	①②	Exhaled carbon monoxide level ≤10 ppm
Chen 2009[82]	USA	Normal smokers	Varenicline<br>Placebo	165<br>168	159/6<br>163/5	Mean: 39<br>Mean: 38.5	Mean: 20.3<br>Mean: 21.3	**Varenicline** 1 mg bd, consisted of a 12-week treatment (all subjects received one 0.5 mg tablet per day for the first 3 days; two 0.5 mg tablets per day for the next 4 days, and two 1 mg tablets from day 8)	24wk	①②	Exhaled carbon monoxide level ≤10 ppm
Hurt 2018[83]	USA	alcohol dependent smokers	Varenicline<br>Placebo	16<br>17	10/6<br>11/6	40.2±11.9<br>38.8±10.4	19.1±7.5<br>21.6±7.3	**Varenicline** 1 mg twice daily, 12 weeks（0.5 mg once daily for 3 days, which increased to 0.5 mg twice daily for days 4 to 7, and then to a target dosage of 1 mg twice daily for 11 weeks）	24wk	②	Exhaled carbon monoxide level ≤8 ppm

Study	Country	Population	Intervention	Sample	Gender M/F	Age†	Cigarettes per day†	Implementation details (process, dosage or duration)	Follow -up	Outcome	Abstinence measurement
Peter 2009[84]	Norway	Normal smokers	Varenicline<br>Placebo	602<br>606	595/613	45.3±10.5	—	**Varenicline** 1 mg twice daily oral dosing, twice daily, 12 weeks	52wk	①	Exhaled carbon monoxide level ≤10 ppm
Nides 2008[85]	USA	Normal smokers	Varenicline<br>Bupropion<br>Placebo	696<br>671<br>685	366/330<br>398/273<br>384/301	43.5±11.3<br>42.5±11.8<br>42.5±11.7	21.8（10-70）<br>21.4（10-65）<br>21.5（10-80）	**Varenicline** 0.5 mg once daily for days 1-3, 0.5 mg BID for days 4-7, then 1 mg BID through week 12)<br>**Bupropion** was titrated to full dosage over 3 days (150 mg once daily for days 1-3, then 150 mg BID through week 12)	52wk	①	Exhaled carbon monoxide level ≤10 ppm
Kevin 2011[86]	USA	Older adolescents	Varenicline<br>Bupropion	15<br>14	7/8<br>8/6	19.1±0.6<br>18.7±1.5	0.9 ± 2.1<br>3.1 ± 4.0	**Varenicline** participants ⩾55 kg received 0.5 mg daily for 3 days, 0.5 mg twice daily for 4 days, and then 1 mg twice daily thereafter, <55 kg received 0.5 mg daily for 7 days and then 0.5 mg twice daily thereafter<br>**Bupropion** participants received 150 mg daily for 7 days and then 300 mg daily thereafter	—	②	Exhaled carbon monoxide level Urine cotinine concentration
Damaris 2017[87]	USA	Substance use disorders with or without depression depression	Varenicline<br>Nicotine	77<br>60	42/35<br>31/29	40.0±10.5<br>39.2±9.7	20.4±11.1<br>18.3±9.3	Twelve weeks of **Varenicline** (2 mg/day, after 1-week dose run-up) **or NRT** (21 mg/day decreasing to 7 mg/day)	24wk	①②	Exhaled carbon monoxide level ⩽ 4 ppm Salivary cotinine level ⩽ 15 ng/ml
Omara 2018[88]	UK	Normal smokers	Varenicline<br>Placebo	253<br>257	213/40<br>216/41	49.9±14.6<br>48.4±15.6	12.3±9.0<br>12.7±9.4	**Varenicline** for 12 weeks: 0.5 mg for 1 week (once on days 1–3, twice on days 4–7) and 1 mg for the subsequent 11 weeks (twice daily)	25wk	①	Exhaled carbon monoxide level ≤10 ppm
Aubin 2008[89]	France	Normal smokers	Varenicline<br>NRT	376<br>370	182/194<br>185/185	42.9±10.5<br>42.9±12.0	23.0 (15–80)<br>22.4(11–60)	**Varenicline** up titrated to 1 mg twice daily for 12 weeks<br>**NRT** (21 mg/day reducing to7 mg/day) for 10 weeks	52wk	①②	Exhaled carbon monoxide level ≤10 ppm
Daniel 2012[90]	USA	Normal smokers	Bupropion<br>NRT<br>Bupropion+NRT<br>Placebo	264<br>267<br>262<br>189	405/577	44.67±11.0 8	—	**Bupropion**, **NRT** lozenge, NRT patch, NRT patch + NRT lozenge, **Bupropion + NRT** lozenge or Placebo<br>All medications were provided for 8 weeks post-quit except the NRT lozenge which was provided for 12 weeks post-quit	—	①②	Exhaled carbon monoxide level < 10 ppm
Yudkin 1996[91]	UK	Normal smokers	NRT<br>Placebo	842<br>844	393/449	24-65 years	—	**NRT** patches in reducing sizes, were used over 12 weeks	52wk	①	Exhaled carbon monoxide level ≤10 ppm<br>Salivary cotinine concentration
David 2006[92]	USA	Normal smokers	Varenicline<br>Bupropion<br>Placebo	352<br>329<br>344	176/176<br>192/137	42.5±11.1<br>42.0±11.7	21.1±9.47<br>21.0±8.52	**Varenicline** 0.5mg/d for days 1 to 3, 0.5 mg twice per day for days 4 to7, then 1mg twice per day through week 12<br>**Bupropion** 150 mg/d for days 1 to 3,	52wk	①②	Exhaled carbon monoxide level ≤10 ppm

Study	Country	Population	Intervention	Sample	Gender M/F	Age†	Cigarettes per day†	Implementation details (process, dosage or duration)	Follow -up	Outcome	Abstinence measurement
								then 150 mg twice per day through week 12			
Fagerström 2010[93]	Sweden	Normal smokers	Varenicline<br>Placebo	447<br>446	389/58	39.3±10.7	22.3(10–60)	**Varenicline** dose was titrated over 1 week (0.5 mg once daily for days 1-3, 0.5 mg BID for days 4-7), then 1 mg BID from day 8, through week 12	24wk	①②	Exhaled carbon monoxide level ≤10 ppm
Mark 2012[94]	Canada	Acute myocardial infarction	Bupropion<br>Placebo	192<br>200	328/64	54.5±10.4	23.2±10.8	**Bupropion** was administered as 150 mg daily for 3 days, followed by 150 mg twice daily for the remainder of the 9-week treatment.	48wk	①②	Exhaled carbon monoxide level ≤10 ppm
Lisa 2004[95]	USA	Normal smokers	Bupropion<br>Placebo	214<br>215	—	≥18 years	≥15 cigarettes	**Bupropion** at a dose of 300 mg per day for 7 weeks (150 mg per day for the first 3 days, followed by 150 mg twice a day)	104wk	②	Exhaled carbon monoxide level ≤10 ppm
Aubin 2004[96]	France	Normal smokers	Bupropion<br>Placebo	340<br>164	150/190<br>74/90	41 years	≥10 cigarettes	**Bupropion** was increased from 150 mg once daily to 150 mg twice daily for day 7	24wk	①②	Exhaled carbon monoxide level
Wagena 2005[97]	Netherlands	COPD	Bupropion<br>Nortriptyline<br>Placebo	86<br>80<br>89	34/52<br>44/36	51.1±8.3<br>51.2±9.1	24.2±9.4<br>22.2±7.6	**Bupropion** 150 mg once daily, for days 1 through 6, followed by 150 mg twice daily for days 7 through 84<br>**Nortriptyline** 25 mg once daily, for days 1 through 3, followed by 50 mg once daily for days 3 through 7, and then 75 mg once daily for days 8 through 84	26wk	①②	Urinary cotinine levels ≤60 ng/mL
Megan 2007[98]	USA	Normal smokers	Bupropion+NRT<br>Bupropion<br>Placebo	228<br>224<br>156	256/352	41.78±11.34	22.44±9.87	**Bupropion** (150 mg, twice daily) +4 mg **NRT** gum,<br>Bupropion (150 mg, twice daily)	48wk	①②	Exhaled carbon monoxide level < 10 ppm
Schneider 1995[99]	USA	Normal smokers	NRT<br>Placebo	128<br>127	66/62<br>74/53	39.9±7.7	28.8±10.9	**NRT** A single squirt into a nostril contains 0.5 mg of nicotine<br>A dose is equal to two squirts—one in each nostril—for a total of 1.0 mg	48wk	①②	Exhaled carbon monoxide level < 8 ppm
Shiffman 2002[100]	UK	Normal smokers	NRT<br>Placebo	459<br>458	393/524	41.11±12.06	17.7±8.2	**NRT** 4 mg lozenge was assigned to those who smoked their first cigarette within 30 minutes of waking, and the 2 mg lozenge was assigned to all others	52wk	①②	Exhaled carbon monoxide level ≤10 ppm
Masakazu 2007[101]	Japan	Normal smokers	Varenicline<br>Placebo	128<br>129	93/35	40.2±12.3	24.9±10.3	**Varenicline** at doses of 0.25 mg BID, 0.5 mg BID, and 1 mg BID with placebo after 12 weeks of treatment	52wk	①②	Exhaled carbon monoxide level ≤10 ppm
Raymond 2008[102]	USA	Normal smokers	Varenicline<br>Placebo	157<br>155	79/78	41.5±11.3	22.2±10.6	**Varenicline** in fixed doses (Week 1, titrated from 0.5 to 1.0 mg/day), followed by a self-regulated flexible schedule (weeks 2-12, 0.5 to 2.0 mg/day)	52wk	①②	Exhaled carbon monoxide level ≤10 ppm
Mercié 2018[103]	France	HIV	Varenicline<br>Placebo	123<br>125	100/23	47±9	19±7	**Varenicline** titrated to two 0.5 mg doses twice daily for 12 weeks	48wk	①	Exhaled carbon monoxide level ≤10 ppm

Study	Country	Population	Intervention	Sample	Gender M/F	Age†	Cigarettes per day†	Implementation details (process, dosage or duration)	Follow -up	Outcome	Abstinence measurement
Nancy 2010[104]	USA	Cardiovascular disease	Varenicline<br>Placebo	355<br>359	267/88	57±8.6	22.1 (10–60)	**Varenicline** 0.5 mg once daily for 3 days, 0.5 mg twice daily for 4 days, and then 1.0 mg twice daily for a total of 12 weeks	52wk	①②	Exhaled carbon monoxide level ≤10 ppm
Cheryl 2006[105]	USA	Normal smokers	Varenicline<br>Placebo	259<br>129	187/201	43.0 ± 9.4<br>42.9 ±10.1<br>43.5 ±10.5	20.9 ± 8.1<br>21.3 ± 8.1<br>20.4 ± 7.2	**Varenicline** 0.5 mg twice daily nontitrated (ie, 0.5 mg twice daily for 12 weeks); 0.5 mg twice daily titrated (ie, 0.5 mg once daily for 7 days, then 0.5 mg twice daily for 11 weeks)	44wk	①②	Exhaled carbon monoxide level ≤10 ppm
Catherine 2020[106]	USA	Anxiety disorders	Varenicline<br>Bupropion<br>NRT<br>Placebo	176<br>180<br>175<br>181	—	18–75 years	≥10 cigarettes	**Varenicline** 1 mg twice daily<br>**Bupropion** 150 mg twice daily<br>**NRT** 21 mg/day with taper, for 12 weeks treatment	24wk	①②	Exhaled carbon monoxide level ≤10 ppm
Mitchell 2020[107]	Sweden	Normal smokers	NRT<br>Placebo	597<br>161	265/332	51.5±11.7	18.1±8.5	**NRT** 1 mg per spray, for 12-week treatment	26wk	①②	Exhaled carbon monoxide level < 10 ppm
Spring 1995[108]	USA	Normal smokers	Fluoxetine<br>Placebo	49<br>48	—	40.5±1.4<br>41.0±1.3	27.3±1.6<br>27.5±1.6	**Fluoxetine** 30mg for 15 weeks	—	①	Exhaled carbon monoxide level < 8 ppm
Neal Doran 2019[109]	USA	Depression	Varenicline<br>Placebo	256<br>126<br>130<br>269	37/89<br>37/90	45.5±11.3<br>45.3±10.6	17.8±8<br>18.9±7.5	**Varenicline** 1 mg twice daily for 12 weeks	52wk	①②	Exhaled carbon monoxide level ≤10 ppm
Murphy 2017[110]	USA	Substance Use Disorders	Varenicline<br>NRT	59<br>51	29/30<br>30/21	40±10.7<br>39.6±9.5	21±12<br>18.4±9.4	**Varenicline** 0.5 mg per day for 3 days, followed by 0.5 mg twice per day for 4 days, followed by 12 weeks of VAR (1 mg twice per day)<br>**NRT** received 12 weeks of NicoDerm patches (4 weeks each of 21 mg/day, 14 mg/day, and 7 mg/day)	—	①②	Exhaled carbon monoxide level ≤10 ppm<br>Salivary cotinine ≤15 ng/ml
Tashkin 2011[111]	USA	COPD	Varenicline<br>Placebo	250<br>254	155/93	57 years	24 to 25 cigarettes	**Varenicline** 0.5 mg once daily for 3 days, 0.5 mg bid for 4 days, then 1.0 mg bid, for a total of 12 weeks	52wk	①②	Exhaled carbon monoxide level ≤10 ppm
Robert 2013[112]	USA	Depression	Varenicline<br>Placebo	256<br>269	97/159	45.4±10.9	21.9±7.5	**Varenicline** 0.5 mg/d for 3 days, 0.5 mg twice daily for 4 days, then 1 mg twice daily for the following 11 weeks	52wk	①②	Exhaled carbon monoxide level ≤10 ppm
Timothy 2016[113]	USA	Normal smokers	NRT<br>Varenicline	241<br>424	125/116<br>222/202	49.4±10.9<br>48.5±11.8	16.4±7.8<br>17.7±7.1	**NRT** 8 weeks of 21 mg, then 2 weeks of 14 mg, and then 2 weeks of 7 mg patches (those smoking 5–10 cigs/day prequit received 10 weeks of 14 mg patches and then 2 weeks of 7 mg patches)<br>**Varenicline** regimen was a 0.5 mg pill 1/day for 3 days, a 0.5 mg pill b.i.d. for 4 days, and a 1 mg pill b.i.d. for 3 days	52wk	①②	Exhaled carbon monoxide level ≤ 9 ppm

Study	Country	Population	Intervention	Sample	Gender M/F	Age†	Cigarettes per day†	Implementation details (process, dosage or duration)	Follow -up	Outcome	Abstinence measurement
Blondal 1999[114]	Sweden	Normal smokers	Fluoxetine<br>Placebo	48<br>52	17/31	41 (25-63)	27±1	**Fluoxetine** start taking a daily dose of 10 mg of fluoxetine 16 days before stopping smoking, then 20 mg 10 days before quitting, continuing for up to at least 3 months	48wk	①	Exhaled carbon monoxide level < 10 ppm
Timothy 2021[115]	USA	Normal smokers	Varenicline<br>Varenicline+NRT	311<br>311	144/167<br>145/166	48.9±12.3<br>49.9±11.5	16.2±7.4<br>16±7.7	**Varenicline** 0.5 mg pill for 3 days, two 0.5 mg pills for 4 days, and two 1 mg pills thereafter (to either week 11 or week 23)<br>**Varenicline+ NRT** Varenicline treatment started with one 0.5-mg pill for 3 days, two 0.5-mg pills for 4 days, and two 1-mg pills thereafter, nicotine patch use started in week 2 and involved use of one 14-mg patch/d until either 12 weeks or 24 weeks	52wk	①②	Exhaled carbon monoxide level ⩽ 6 ppm
David 1995[116]	USA	Normal smokers	NRT<br>Placebo	32<br>28	34/26	43.7±9.5	≥25 cigarettes	**NRT** use 12 pieces per day of their assigned dosage formulation, 4 mg, 2 mg, or 0.5 mg (placebo) of nicotine polacrilex	—	①②	Serum cotinine level ⩽250 ng/mL<br>Exhaled carbon monoxide level ⩽15 ppm
Shiffman 2005[117]	USA	Normal smokers	NRT<br>Placebo	450<br>451	—	43.1±10.2	30.6±10.4	**NRT** patch 21 mg/day for the first 6 weeks of treatment	24wk	①	Exhaled carbon monoxide level ⩽8 ppm
Ebbert 2015[118]	USA	Normal smokers	Varenicline<br>Placebo	760<br>750	425/335	44.7±11.8	20.6±8.5	**Varenicline** dosage of 0.5 mg once daily for 3 days, increasing to 0.5 mg twice daily for days 4 to 7, and of 1 mg twice daily for 12weeks	52wk	①②	Exhaled carbon monoxide level ⩽10 ppm
Christian 2015[119]	Denmark	Asthma	Varenicline<br>Placebo	26<br>25	33/18	31.3±5.5	—	**Varenicline** Day 1-3: 0.5 mg once daily, Day 4-7, 0.5 mg twice daily, Day 8 to week 12, 1.0 mg twice daily<br>ICS period, week 12 to week 24, Inhaled Budesonide 400 mg twice daily	24wk	①	Exhaled carbon monoxide level ⩽10 ppm
Agneta 1994[120]	Sweden	Normal smokers	NRT<br>Placebo	125<br>123	53/72	44.9±11.5	21.2±5.9	**NRT** 0.5 mg per single spray, through 12weeks	48wk	①②	expired carbon monoxide less than 10 ppm
Serena 2006[121]	Norway	Normal smokers	Varenicline<br>Placebo	603<br>607	303/300	45.4±10.4	20.7±7.3	**Varenicline** 0.5 mg daily for 3 days, 0.5 mg twice daily for 4 days, then 1 mg twice daily for 11 weeks	52wk	①②	Exhaled carbon monoxide level ⩽10 ppm
Ryan 2021[122]	Australia	Normal smokers	Cytisine<br>Varenicline	725<br>727	363/362<br>347/380	42.8±13.1<br>42.9±12.3	18.3±8<br>17.8±8	**Cytisine** 1.5-mg/d capsules for a 25-day<br>**Varenicline** 0.5-mg tablet and then 2 tablets for days 4-7, on day 8, they quit smoking and started taking 1-mg tablet taken twice daily for an 84-day (12 weeks) treatment	28wk	①②	Exhaled carbon monoxide level ⩽ 9 ppm

Study	Country	Population	Intervention	Sample	Gender M/F	Age†	Cigarettes per day†	Implementation details (process, dosage or duration)	Follow -up	Outcome	Abstinence measurement
Andrea 2022[123]	USA	Drink Heavily	Varenicline+NRT<br>NRT	61<br>61	35/26<br>32/29	44±12.9<br>44±12	11.8±6.3<br>11.8±7.1	**Varenicline** 0.5 mg once daily for 3 days, 0.5 mg twice daily for 4 days, and 1.0 mg twice daily, through 12 weeks of treatment<br>**NRT** 14 mg patches to use daily for the first 6 weeks, then 7 mg patches to use daily for 4 weeks	—	①②	Exhaled carbon monoxide level ⩽10 ppm
Roldano 2007[124]	Italy	Normal smokers	Bupropion<br>Placebo	400<br>193	248/152	Mean: 49.4<br>Mean: 48.5	21.1±8.7	**Bupropion** 150 mg/d for 6 days followed by 150 mg twice a day for 7 weeks	52wk	①②	Exhaled carbon monoxide level ⩽10 ppm
Andrea 2011[125]	USA	Normal smokers	Selegiline<br>Placebo	51<br>50	52/49	48.5 ± 11.0	22.2 ± 7.1	**Selegiline** 5mg/d for 7days, the dose of medication was increased to 5mg twice daily on Day 8. received 9 weeks of medication with a maximum dose of 10 mg/day	24wk	①②	Exhaled carbon monoxide level ＜ 10 ppm<br>Plasma cotinine level <15 ng/ml
Kathryn 2007[126]	USA	Normal smokers	Varenicline<br>Placebo	251<br>126	127/124	48.2±12.3	23.2(10-90)	**Varenicline** 1 mg twice daily for 12 weeks.	52wk	①②	Exhaled carbon monoxide level ⩽10 ppm
Christopher 2017[127]	USA	Heavy-Drinking	Naltrexone<br>Placebo	75<br>75	42/33	—	16.9 ± 7.2	**Naltrexone** 21 mg for two weeks, followed by 14 mg for 2 weeks and then 7 mg for 2 weeks	26wk	①②	Exhaled carbon monoxide level ⩽ 4 ppm
Dahlia 2011[128]	USA	Normal smokers	Varenicline<br>Placebo	55<br>55	33/22	33.4 ±11.8	21.3(10-40)	**Varenicline** equivalents were administered at 5 mg once daily (first 3 days), then 5 mg twice daily (b.i.d., next 4 days), followed by 11 weeks of 1 mg BID	—	①②	Exhaled carbon monoxide level ⩽10 ppm
Evins 2005[129]	USA	Schizophrenia	Bupropion<br>Placebo	25<br>28	39/14	46±9.4	34.2±20.4	**Bupropion** 150 mg/d for 7 days, 300mg/d, for 11weeks	—	①②	Exhaled carbon monoxide level ＜ 9 ppm
Omara 2020[130]	UK	Tuberculosis	Cytisine<br>Placebo	1239<br>1233	1277/12<br>1221/12	42.5±14.3<br>42.4±14.2	11.1±8.6	**Cytisine** orally as six 1.5 mg capsules per day, which was gradually reduced to 1.5 mg (one capsule) by day 25, with a quit date set for day 5, through 12weeks	48wk	①②	Exhaled carbon monoxide level ⩽10 ppm
Stephanie 2006[131]	Germany	Normal smokers	Naltrexone<br>Placebo	93<br>93	48/45	45.2±1.16	28.1±1.18	**Naltrexone** 12.5 mg for 1 day, 25 mg for 1 day, 50 mg for 2 days, and 100 mg thereafter	48wk	①②	Exhaled carbon monoxide level ⩽10 ppm
Hurt 1997[132]	USA	Normal smokers	Bupropion<br>Placebo	153<br>153	128/178	44.1±10.5	26.2±8.5	**Bupropion** at a dose of 100 mg per day (50 mg twice a day), 150 mg per day (150 mg each morning and placebo each evening), or 300 mg per day (150 mg per day for 3 days, followed by 150 mg twice a day)	48wk	①	Exhaled carbon monoxide level ⩽10 ppm

Study	Country	Population	Intervention	Sample	Gender M/F	Age†	Cigarettes per day†	Implementation details (process, dosage or duration)	Follow -up	Outcome	Abstinence measurement
Josep 2014[133]	Spain	Normal smokers	Varenicline+NRT<br>Varenicline	170<br>171	95/75<br>102/69	44.1±14.8<br>46.2±13.1	⩾20 cigarettes	**Varenicline+NRT** 0.5 mg once daily for 3 days, then 0.5 mg twice daily for 4 days, followed by 1 mg twice daily for 11 weeks<br>**Varenicline** 0.5 mg once daily for 3 days, then 0.5 mg twice daily for 4 days, followed by 1 mg twice daily for 11 weeks	24wk	①②	Exhaled carbon monoxide level ＜ 10 ppm
Ebbert 2014[134]	USA	Normal smokers	Varenicline+Bupropion<br>Varenicline	249<br>257	136/113<br>131/126	42.2±12.2<br>41.9±12.7	19.5±7.3<br>19.7±7.9	**Varenicline** 0.5 mg once daily for 3 days, increasing to 0.5 mg twice daily for days 4 to 7, and 1 mg twice daily for 11 weeks<br>**Bupropion** titrated 1 tablet (150 mg) by mouth once per day for days 1 to 3, then 1 tablet by mouth twice per day for 12 weeks	52wk	①②	Exhaled carbon monoxide level ⩽8 ppm
Brent 2014[135]	New Zealand	Normal smokers	NRT<br>Placebo	716<br>707	341/375<br>318/389	45.6±11.4	45.6±11.4<br>45.3±11.9	**NRT** 21 mg for the first 18 weeks, followed by 14 mg, and then 7 mg in the final 4 weeks	48wk	①②	Exhaled carbon monoxide level ＜ 10 ppm
Brent 2016[136]	New Zealand	Normal smokers	NRT<br>Placebo	246<br>256	124/122	45.2±11.4	19±6.7	**NRT** patches for 5 months, use 21 mg/24 h for 18 weeks, 14 mg/24 h for 2 weeks, and 7 mg/24 h for 2 weeks	24wk	①②	Exhaled carbon monoxide level
Tatiana 2017[137]	USA	Pregnant Smokers	Bupropion<br>Placebo	30<br>35	0/65	24.5±5.56	—	**Bupropion** assigned to a 12-week, twice a day treatment with 150 mg	24wk	①②	Exhaled carbon monoxide of <4 ppm<br>Urinary cotinine level of < 50 ng/mL
Lowell 2007[138]	USA	Normal smokers	Bupropion<br>Placebo	113<br>112	—	37.9±9.1	—	**Bupropion** titrated to 150 mg twice daily for 12 weeks	52wk	①②	Urine cotinine of < 50 ng/mL.
Tonstad 2003[139]	Norway	Cardiovascular disease	Bupropion<br>Placebo	313<br>313	463/163	55.6±9.2	25.2±12.2	**Bupropion** 150 mg/day on days 1-3, 150 mg twice daily on days 4-49	52wk	①②	Exhaled carbon monoxide level
Gonzales 2001[140]	USA	Normal smokers	Bupropion<br>Placebo	226<br>224	117/109	44.5±11.8	—	**Bupropion** 150 mg daily on days 1-3 and then 150 mg twice daily for the 12 weeks treatment	—	①	Exhaled carbon monoxide level ⩽10 ppm
Robert 2010[141]	USA	Cancer patients	Bupropion<br>Placebo	114<br>132	49/65<br>81/51	Mean: 52.6<br>Mean: 54	Mean: 16<br>Mean: 18.3	**Bupropion** dose of 300 mg/day (two 150 mg tablets) was used and treatment began with one 150 mg pill taken each day for 7 days and then two 150 mg pills/day (separated by 8 h) for days 8-63	27wk	①②	Exhaled carbon monoxide level ⩽10 ppm
David 2011[142]	Israel	Acute coronary syndrome	Bupropion<br>Placebo	75<br>76	57/18	52.4±11	32.3±16	**Bupropion** 150 mg, or identical placebo, once a day for 3 days, and then twice a day for 8 weeks	48wk	①②	Self-reported

Study	Country	Population	Intervention	Sample	Gender M/F	Age†	Cigarettes per day†	Implementation details (process, dosage or duration)	Follow -up	Outcome	Abstinence measurement
Lisa 2012[143]	USA	Normal smokers	Bupropion<br>Placebo	270<br>270	196/174	46.8±11.1	8.0±2.6	**Bupropion** 150 mg daily for 3 days and then 150 mg twice daily for the remaining 46 days	26wk	①②	Exhaled carbon monoxide level ≤10 ppm
Joel 2004[144]	USA	Normal smokers	Bupropion<br>Placebo	121<br>123	106/15	50±12	22±15	**Bupropion** 7-week course of sustained-release bupropion hydrochloride (150 mg daily for the first 3 days, then 150 mg twice daily)	52wk	①	Saliva cotinine levels of 15 ng/mL
Nancy 2006[145]	USA	Acute cardiovascular disease	Bupropion<br>Placebo	124<br>124	93/31	56.7±9.7	23.1±13.9	**Bupropion** 150 mg was prescribed daily for 3 days then twice daily for a total of 12 weeks	48wk	①②	Salivary cotinine level ＞20 ng/ml Exhaled carbon monoxide level
Weinberger 2008[146]	USA	Bipolar Disorder	Bupropion<br>Placebo	2<br>3	2/3	57.2 ± 6.8	—	Bupropion initiated on day 1 of the 10 weeks trial at 75 mg×3 days, then increased to 150 mg×4 days, and then increased to a final dose of up to 150 mg po bid (300 mg/day) by Day 15 (target quit date; TQD) as tolerated.	—	—	Exhaled carbon monoxide level ＜ 10 ppm
Elaine 2011[147]	USA	Schizophrenia	Varenicline<br>Placebo	4<br>4	3/1<br>3/1	46.3 ± 9.0<br>44.3 ± 5.1	⩾10 cigarettes	**Varenicline** 1mg twice daily, until the end of 12 weeks treatment.	—	①	Exhaled carbon monoxide level ＜ 10 ppm
George 2007[148]	Canada	Schizophrenia	Bupropion +NRT<br>NRT	29<br>29	29<br>29	41.2 ± 9.2<br>39.3 ± 6.9	24.3 ± 10.3<br>22.4 ± 11.9	**Bupropion** Day 8 of the trial at 150 mg× 3 days, and then increased to 150 mg po bid and continued until the end of the trial (Day 70).<br>**NRT** was applied at day 15 concurrent with the target quit date, and continued until Day 70.	24wk	①②	Exhaled carbon monoxide level ＜ 10 ppm
Mao 2020[149]	France	COPD	Varenicline<br>Placebo	42<br>39	24/18<br>25/14	56.0±8.8<br>57.6±8.7	Mean: 23.5<br>Mean: 22.8	**Varenicline** 0.5 mg/d, 3 days, 1 mg/d, 4 days, 2 mg/d, to the end of 12weeks treatment.	52wk	①②	Exhaled carbon monoxide level ⩽ 10 ppm
Qin 2021[150]	China	COPD	Varenicline<br>Bupropion	68<br>68	67/1<br>65/3	55~70 years	—	**Varenicline** 0.5 mg once per day for the first 3 days; 0.5 mg twice per day for the next 4 days; 1 mg twice per day from day 8.<br>**Bupropion** one 150 mg tablet per day, through 12 weeks the end of the trial.	24wk	②	Exhaled carbon monoxide level ＜ 10 ppm
Daniel 2014[151]	USA	Heavy drinking	Naltrexone<br>Placebo	34<br>35	43/26	35.5 ± 10.8	15.6 ± 5.6	**Naltrexone** 12.5 mg/d (day 1) to 25 mg/d (days 2 and 3) and then to the target dose of 50 mg/d, day 4, through the end of the trial.	48wk	①	Exhaled carbon monoxide level ＜ 10 ppm

Study	Country	Population	Intervention	Sample	Gender M/F	Age†	Cigarettes per day†	Implementation details (process, dosage or duration)	Follow -up	Outcome	Abstinence measurement
Andrea 2009[152]	USA	Heavy drinking	Naltrexone<br>Placebo	34<br>44	19/15<br>24/20	42.2 ± 2.0<br>42.5 ± 1.8	23.4 ± 1.4<br>19.90 ± 1.0	**Naltrexone** 3 days prior to the quit date with an initial dose of 25 mg oral naltrexone, until the end of 8 weeks treatment.	—	①	Exhaled carbon monoxide level ＜ 10 ppm
Lisa 2011[153]	USA	Heavy drinking	Varenicline<br>Placebo	15<br>15	16/14	42.87±8.52<br>43.47±7.99	22.17 ± 7.31<br>18.88 ± 6.85	**Varenicline** 2 mg daily, until the end of 8weeks treatment.	—	②	Exhaled carbon monoxide level ⩽ 10 ppm
Kathleen 2007[154]	USA	Alcohol dependence	Bupropion<br>Placebo	30<br>28	25/5<br>24/4	38.5±11.7<br>40.8±11.3	27.0±9.8<br>23.2±5.9	**Bupropion** 21 mg (four weeks), 14 mg (two weeks), and 7 mg (two weeks).	24wk	①②	Exhaled carbon monoxide level
Stephanie 2009[155]	USA	Alcohol dependence	Naltrexone<br>Placebo	28<br>26	18/10<br>15/11	46.21±7.84<br>41.19±11.5 7	27.55±10.35<br>26.59±7.83	**Naltrexone** 25mg,50mg,100mg/day, until the end of 6weeks treatment.	—	①	Exhaled carbon monoxide level ⩽ 10 ppm
Robert 2017[156]	USA	Alcohol dependence	Topiramate<br>Placebo	63<br>66	129/0	47.2±9.0<br>46.9±9.8	19.7±7.0<br>21.2 ±7.4	**Topiramate** up to 100 mg twice daily, until the end of 12weeks treatment.	24wk	②	Exhaled carbon monoxide level ⩽ 10 ppm
Krysten 2019[157]	USA	Alcohol use disorder	Varenicline<br>Placebo	64<br>67	92/39	42.7±11.70	11.6±7.24	**Varenicline** 1mg twice daily to the end of 16weeks treatment.	48wk	①	Urine cotinine level of ⩾ 30 ng/mL<br>plasma cotinine level of ⩾ 6 ng/mL
Stephanie 2017[158]	USA	Alcohol use disorder	Varenicline<br>Placebo	64<br>67	45/19<br>47/20	42.7±11.7	13±8<br>12±7<br>10±6<br>8±6	**Varenicline** 0.5 mg once daily for 3 days, 0.5 mg twice daily for 4 days, and 1 mg twice daily for the remainder of the 16-week treatment.	19wk	①	Plasma cotinine levels of less than 6 ng/mL
Tindle 2022[159]	USA	Heavy drinking	Varenicline<br>Cytisine	100<br>100	66/34<br>69/31	39 ± 6<br>38 ± 6	22 ± 8<br>21 ± 7	**Varenicline** received 12 weeks of active varenicline and 8 weeks of NRT placebo mouth spray.<br>**Cytisine** dosing followed the traditional 25-day downward titration schedule of 1.5 mg tablets: 6 tablets/d (days 1-3), 5 tablets/d (days 4-12), 4 tablets/d (days 13-16), 3 tablets/d (days 17-20), and 1 to 2 tablets/d (days 21-25).	48wk	②	Exhaled carbon monoxide level ＜ 10 ppm

Gender: M: male; F: female; NRT: Nicotine Replacement Therapy; USA: United States of America; UK: United Kingdom; wk: week; ppm: parts per million

①: Continued abstinence; ②: 7-day point abstinence. †Data can be presented in the following formats: mean± standard deviation, median (range), mean, or range.

Supplementary Table 3. The results of bias risk assessment

Author (year)	Random sequence generation	Allocation concealment	Blinding of participants and personnel	Blinding of outcome assessment	Incomplete outcome	Selective reporting	Other biases	Overall
King 2022	Low	Unclear	Unclear	Unclear	Low	Low	Low	Unclear
King 2006	Low	Unclear	Low	Unclear	Low	Low	Low	Unclear
Hughes 2003	Unclear	Unclear	Unclear	Unclear	Low	Low	Low	Unclear
Hoogsteder 2014	Low	Low	Low	Low	Low	Low	Low	Low
Nakamura 2017	Low	Low	Low	Low	Low	Low	Low	Low
Hughes 2011	Unclear	Low	Low	Unclear	Low	Low	Low	Unclear
Pfeifer 2019	Unclear	Unclear	Unclear	Unclear	Low	Low	Low	Unclear
Gray 2014	Unclear	Low	Low	Low	Low	Low	Low	Unclear
Bohadana 2019	Low	Low	Low	Low	Low	Low	Low	Low
Sun 2009	Low	Unclear	Unclear	Unclear	Low	Low	Low	Unclear
Gray 2020	Low	Low	Low	Unclear	Low	Low	Low	Unclear
Kalman 2011	Low	Low	Unclear	Unclear	Low	Low	Low	Unclear
Jamerson 2001	Unclear	Unclear	Unclear	Unclear	Low	Low	Low	Unclear
Scherphof 2014	Low	Unclear	Unclear	Unclear	Low	Low	Low	Unclear
Benjamin 2010	Unclear	Unclear	Low	Unclear	Low	Low	Low	Unclear
Nides 2021	Unclear	High	High	Low	Low	Low	Low	High
Dalsgarð 2003	Low	Unclear	Unclear	Unclear	Low	Low	Low	Unclear
Tønnesen 2003	Unclear	Unclear	Unclear	Unclear	Low	Low	High	High
Ebbert 2014	Low	Low	Low	Low	Low	Low	Low	Low
Covey 2014	Unclear	Unclear	Unclear	Unclear	Low	Low	Low	Unclear
Oncken 2019	Low	Low	Low	Unclear	Low	Low	Low	Unclear
Oncken 2008	Low	Low	Unclear	Unclear	Low	Low	Low	Unclear
Caldwell 2016	Low	Low	Low	Unclear	Low	Low	Low	Unclear
Bohadana 2000	Low	Unclear	Unclear	Unclear	Low	Low	Low	Unclear
Hjalmerson 1997	Unclear	Unclear	Low	Unclear	Low	Low	Low	Unclear
Hurt 1994	Unclear	Unclear	Unclear	Unclear	Low	Low	Low	Unclear
WGSTNP 1994	Low	Low	Unclear	Unclear	Low	Low	Low	Unclear
Etter 2001	Low	Low	High	Unclear	Low	Low	High	High
Rose 1998	Unclear	Unclear	Unclear	Unclear	Low	Low	Low	Unclear
Aveyard 2008	Low	Low	Low	Unclear	Low	Low	Low	Unclear
Wong 2013	Low	Low	Low	Unclear	Low	Low	Low	Unclear
Ebbert 2011	Low	Low	Low	Low	Low	Low	Low	Low
Cooper 2004	Unclear	Unclear	Low	Unclear	Low	Low	Low	Unclear
George 2002	Unclear	Low	Unclear	Unclear	Low	Low	Low	Unclear

Author (year)	Random sequence generation	Allocation concealment	Blinding of participants and personnel	Blinding of outcome assessment	Incomplete outcome	Selective reporting	Other biases	Overall
Etter 2006	Low	Low	High	Unclear	Low	Low	High	High
Etter 2004	Low	Low	High	Unclear	Low	Low	High	High
George 2006	Unclear	Low	Low	Unclear	Low	Low	Low	Unclear
Hertzberg 2001	Unclear	Unclear	Unclear	Unclear	Unclear	Low	Low	Unclear
Killen 2004	Unclear	Unclear	Low	Unclear	Low	Low	Low	Unclear
Croghan 2007	Low	Unclear	Unclear	Unclear	Low	Low	Low	Unclear
Tsukahara 2015	Low	Unclear	High	High	Unclear	Low	Low	High
Schnoll 2019	Unclear	Low	Low	Unclear	Low	Low	Low	Unclear
Piper 2009	Unclear	Unclear	Low	Low	Unclear	Low	Low	Unclear
Rennard 2011	Low	Low	Low	Low	Unclear	Low	Low	Unclear
Wong 1999	Unclear	Low	Unclear	Unclear	Unclear	Low	Low	Unclear
John 2012	Low	Low	Unclear	Unclear	Unclear	Low	Low	Unclear
Evins 2007	Unclear	Unclear	Low	Low	Unclear	Low	Low	Unclear
Hays 2009	Unclear	Unclear	Low	Low	Unclear	Low	Low	Unclear
Franks 1989	Unclear	Unclear	Low	Low	Low	Low	Low	Unclear
Elbert 2007	Low	Unclear	Low	Low	Unclear	Low	Low	Unclear
Williams 2012	Unclear	Unclear	Low	Low	Unclear	Low	Low	Unclear
Cinciripini 2018	Unclear	Unclear	Low	Low	Low	Low	Low	Unclear
Zawertailo 2020	Unclear	Unclear	Unclear	Unclear	Low	Low	Low	Unclear
Poling 2010	Unclear	Unclear	Low	Low	Unclear	Low	Low	Unclear
Scherphof 2014	Unclear	Unclear	Low	Low	Low	Low	Low	Unclear
Sarah 2018	Unclear	Unclear	Low	Low	Unclear	Low	Low	Unclear
Cooney 2009	Unclear	Unclear	Low	Low	Unclear	Low	Low	Unclear
Tonstad 2006	Unclear	Unclear	Low	Low	Unclear	Low	Low	Unclear
Eva 2009	Unclear	Unclear	Low	Low	Unclear	Low	Low	Unclear
Nides 2006	Unclear	Unclear	Low	Low	Unclear	Low	Low	Unclear
Duška 2003	Unclear	Low	Low	Low	Unclear	Low	Low	Unclear
Cooper 2014	Low	Low	Low	Low	Unclear	Low	Low	Unclear
Fagerstrom 2010	Unclear	Unclear	Low	Low	Unclear	Low	Low	Unclear
Costa 2002	Low	Low	Low	Low	Unclear	Low	Low	Unclear
Paul 2016	Unclear	Low	Low	Low	Unclear	Low	Low	Unclear
Hays 2001	Low	Low	Low	Low	Unclear	Low	Low	Unclear
Steinberg 2011	Low	Low	Low	Low	Low	Low	Low	Unclear
Cheryl 2013	Low	Low	Low	Low	Unclear	Low	Low	Unclear
Allan 1992	Unclear	Unclear	Unclear	Unclear	Unclear	Low	Low	Unclear
John 2011	Low	Low	Low	Low	Unclear	Low	Low	Unclear

**Author (year)**	**Random sequence generation**	**Allocation concealment**	**Blinding of participants and personnel**	**Blinding of outcome assessment**	**Incomplete outcome**	**Selective reporting**	**Other biases**	**Overall**
Michael 2009	Low	Low	High	High	Unclear	Low	Low	High
Jamshid 2003	Unclear	Unclear	Low	Low	Unclear	Low	Low	Unclear
Elin 2006	Unclear	Unclear	Unclear	Unclear	Unclear	Low	Low	Unclear
Bankole 2005	Unclear	Unclear	Low	Low	Unclear	Low	Low	Unclear
Hajek 2011	Low	Low	Low	Low	Low	Low	Low	Low
Piper 2008	Unclear	Unclear	Low	Low	Unclear	Low	Low	Unclear
Tuisku 2016	Unclear	Low	Unclear	Unclear	Unclear	Low	Low	Unclear
Evan 2018	Unclear	Low	Low	Low	Unclear	Low	Low	Unclear
Stein 2013	Unclear	Unclear	Low	Low	Unclear	Low	Low	Unclear
Ebbert 2016	Low	Low	Low	Low	Low	Low	Low	Low
Antoniu 2011	Unclear	Unclear	Low	Low	Unclear	Low	Low	Unclear
Wang 2009	Unclear	Unclear	Low	Low	Unclear	Low	Low	Unclear
Hurt 2018	Low	Unclear	Unclear	Unclear	Unclear	Low	Low	Unclear
Hajek 2009	Unclear	Unclear	Unclear	Unclear	Unclear	Low	Low	Unclear
Nides 2008	Low	Unclear	Low	Low	Unclear	Low	Low	Unclear
Gray 2011	Unclear	Unclear	Low	Low	Unclear	Low	Low	Unclear
Rohsenow 2017	Unclear	Unclear	Low	Low	Unclear	Low	Low	Unclear
Dogar 2018	Low	Low	Low	Low	Unclear	Low	Low	Unclear
Aubin 2008	Low	High	Unclear	Unclear	Low	Low	Low	High
Daniel 2012	Low	Low	Low	Unclear	Low	Low	Low	Unclear
Yudkin 1996	Unclear	Unclear	Low	Unclear	Low	Low	Low	Unclear
David 2006	Low	Unclear	Low	Unclear	Low	Low	Low	Unclear
Fagerström 2010	Unclear	Unclear	Low	Unclear	Unclear	Low	Low	Unclear
Eisenberg 2012	Low	Unclear	Low	Unclear	Low	Low	Low	Unclear
Lisa 2004	Unclear	High	Low	Unclear	Low	Low	Low	High
Aubin 2004	Unclear	Unclear	Low	Unclear	Low	Low	Low	Unclear
Wagena 2005	Unclear	Unclear	Low	Unclear	Low	Low	Low	Unclear
Megan 2007	Unclear	Unclear	Low	Unclear	Low	Low	Low	Unclear
Schneider 1995	Unclear	Unclear	Low	Unclear	Low	Low	Low	Unclear
Shiffman 2002	Unclear	Unclear	Low	Unclear	Unclear	Low	Low	Unclear
Nakamura 2007	Low	Unclear	Low	Unclear	Unclear	Low	Low	Unclear
Niaura 2008	Low	Unclear	Low	Unclear	Low	Low	Low	Unclear
Mercié 2018	Low	High	Low	Unclear	Low	Low	Low	High
Rigotti 2010	Low	Low	Low	Unclear	Low	Low	Low	Unclear
Oncken 2006	Unclear	Unclear	Low	Unclear	High	Low	Low	High
Catherine 2020	Low	Unclear	Low	Unclear	Low	Low	Low	Unclear

Author (year)	Random sequence generation	Allocation concealment	Blinding of participants and personnel	Blinding of outcome assessment	Incomplete outcome	Selective reporting	Other biases	Overall
Nides 2020	Low	Unclear	Low	Unclear	Low	Low	Low	Unclear
Spring 1995	Unclear	Unclear	Low	Unclear	Unclear	Low	Low	Unclear
Doran 2019	Unclear	Unclear	Low	Unclear	Low	Low	Low	Unclear
Murphy 2017	Low	Unclear	Low	Unclear	Unclear	Low	Low	Unclear
Tashkin 2011	Low	Unclear	Low	Unclear	Unclear	Low	Low	Unclear
Anthenelli 2013	Unclear	Unclear	Low	Unclear	Low	Low	Low	Unclear
Timothy 2016	Low	High	Low	Unclear	Low	Low	Low	High
Blondal 1999	Low	Unclear	Low	Unclear	Low	Low	Low	Unclear
Timothy 2021	Low	Unclear	Low	Unclear	Low	Low	Low	Unclear
David 1995	Unclear	Unclear	Low	Unclear	Low	Low	Low	Unclear
Shiffman 2005	Unclear	Unclear	Low	Unclear	Low	Low	Low	Unclear
Ebbert 2015	Low	Unclear	Low	Low	Unclear	Low	Low	Unclear
Christian 2015	Unclear	Low	Low	Unclear	Low	Low	Low	Unclear
Agneta 1994	Unclear	Unclear	Low	Unclear	Low	Low	Low	Unclear
Serena 2006	Low	High	Low	Unclear	Low	Low	Low	High
Ryan 2021	Low	High	Low	Low	Low	Low	Low	High
Andrea 2022	Low	Unclear	Low	Unclear	Low	Low	Low	Unclear
Roldano 2007	Unclear	Unclear	Low	Unclear	Low	Low	Low	Unclear
Andrea 2011	Unclear	Unclear	Low	Unclear	Low	Low	Low	Unclear
Kathryn 2007	Unclear	Unclear	Low	Unclear	Unclear	Low	Low	Unclear
Christopher 2017	Low	Unclear	Low	Unclear	Low	Low	Low	Unclear
Dahlia 2011	Low	Unclear	Low	Unclear	Low	Low	Low	Unclear
Evins 2005	Unclear	Unclear	Low	Unclear	Low	Low	Low	Unclear
Omara 2020	Low	Unclear	Low	Unclear	Low	Low	Low	Unclear
Stephanie 2006	Low	Unclear	Low	Unclear	Low	Low	Low	Unclear
Hurt 1997	Unclear	Unclear	Low	Unclear	Unclear	Low	Low	Unclear
Josep 2014	Low	Unclear	Low	Unclear	Low	Low	Low	Unclear
Ebbert 2014	Low	High	Low	Unclear	Low	Low	Low	High
Brent 2014	Low	Unclear	Low	Unclear	Low	Low	Low	Unclear
Brent 2016	Unclear	Unclear	Low	Unclear	Low	Low	Low	Unclear
Tatiana 2017	Low	Unclear	Low	Low	Low	Low	Low	Unclear
Lowell 2007	Unclear	Unclear	Low	Unclear	Low	Low	Low	Unclear
Tonstad 2003	Unclear	Unclear	Low	Unclear	Low	Low	Low	Unclear
Gonzales 2001	Unclear	Unclear	Low	Unclear	Low	Low	Low	Unclear
Robert 2010	Unclear	Unclear	Low	Unclear	Low	Low	Low	Unclear
David 2011	Unclear	Unclear	Low	Unclear	Low	Low	Low	Unclear
Lisa 2012	Unclear	Unclear	Low	Unclear	Low	Low	Low	Unclear

**Author (year)**	**Random sequence generation**	**Allocation concealment**	**Blinding of participants and personnel**	**Blinding of outcome assessment**	**Incomplete outcome**	**Selective reporting**	**Other biases**	**Overall**
Joel 2004	Low	Unclear	Low	Unclear	Low	Low	Low	Unclear
Nancy 2006	Unclear	Unclear	Low	Unclear	Low	Low	Low	Unclear
Qin 2021	Low	Low	Low	Unclear	Low	Low	Low	Unclear
Mao 2020	Low	Low	Low	Unclear	Low	Low	Low	Unclear
Tindle 2022	High	Unclear	Unclear	Unclear	Low	Low	Low	High
Stephanie 2017	Low	Low	Low	Low	Low	Low	Low	Low
Krysten 2019	Low	Unclear	Low	Unclear	Low	Low	Low	Unclear
Robert 2017	Low	Low	Low	Low	Low	Low	Low	Low
Stephanie 2009	Low	Unclear	Low	Low	Low	Low	Low	Unclear
Kathleen 2007	Low	Low	Low	Low	Low	Low	Low	Low
Lisa 2011	Low	Low	Low	Low	Low	Low	Low	Low
Andrea 2009	Low	Unclear	Low	Low	Low	Low	Low	Unclear
Daniel 2014	Low	Unclear	Low	Low	Low	Low	Low	Unclear
Elaine 2011	Unclear	Unclear	Unclear	Unclear	Low	Low	Low	Unclear
Weinberger 2008	Unclear	Unclear	Unclear	Unclear	Low	Low	Low	Unclear
George 2007	Unclear	Unclear	Unclear	Unclear	Low	Low	Low	Unclear

Supplementary table 4. The evidence findings for all comparisons

№ of studies	Certainty assessment					№ of patients		Effect	Certainty
	Risk of bias	Inconsistency	Indirectness	Imprecision	Publication bias	Intervention-1	Intervention-2	OR with 95% CI	
**Bupropion – Bupropion + NRT**									
**8**	Serious	Not serious	Not serious	Not serious	Undetected	2066	1886	0.87 (0.63,1.20)	Moderate
**Bupropion - NRT**									
**5**	Serious	Not serious	Not serious	Not serious	Undetected	1534	1544	0.93 (0.75,1.16)	Moderate
**Bupropion - Nortriptyline**									
**1**	Serious	Not serious	Not serious	Very serious	Undetected	86	80	1.10 (0.58,2.09)	Very low
**Bupropion - Placebo**									
**34**	Very serious	Not serious	Not serious	Not serious	Undetected	5389	5503	1.70 (1.43,2.03)	Low
**Bupropion - Varenicline**									
**6**	Serious	Not serious	Not serious	Not serious	Undetected	1659	1591	0.66 (0.53,0.82)	Moderate
**Bupropion + NRT - NRT**									
**8**	Serious	Not serious	Not serious	Not serious	Undetected	1621	1854	1.07 (0.78,1.47)	Moderate
**Bupropion + NRT - Placebo**									
**5**	Serious	Not serious	Not serious	Serious	Undetected	1075	809	1.95 (1.42,2.68)	Low
**Clonidine - NRT**									
**1**	Serious	Not serious	Not serious	Very serious	Undetected	57	57	0.63 (0.32,1.22)	Very low
**Clonidine - Naltrexone**									
**1**	Serious	Not serious	Not serious	Very low	Undetected	57	57	1.16 (0.54,2.49)	Very low
**Clonidine - Placebo**									
**2**	Serious	Not serious	Not serious	Serious	Undetected	198	200	1.15 (0.60,2.20)	Low
**Cytisine - Placebo**									
**2**	Serious	Not serious	Not serious	Not serious	Undetected	1290	1284	2.06 (1.20,3.52)	Moderate

№ of studies	Certainty assessment					№ of patients		Effect	Certainty
	Risk of bias	Inconsistency	Indirectness	Imprecision	Publication bias	Intervention-1	Intervention-2	OR with 95% CI	
**Cytisine - Varenicline**									
**2**	Serious	Not serious	Not serious	Serious	Undetected	825	827	0.80 (0.47,1.36)	Low
**Fluoxetine - Placebo**									
**2**	Serious	Not serious	Not serious	Very serious	Undetected	97	100	1.23 (0.52,2.92)	Very low
**NRT – NRT + Mecamylamine**									
**2**	Very serious	Not serious	Not serious	Very serious	Undetected	41	41	0.29 (0.08,1.08)	Very low
**NRT - Naltrexone**									
**2**	Serious	Very serious	Not serious	Very serious	Undetected	80	82	1.84 (1.16,2.93)	Very low
**NRT - Placebo**									
**39**	Very Serious	Very serious	Not serious	Not serious	Undetected	8616	7908	1.83 (1.56,2.14)	Very low
**NRT - Topiramate**									
**1**	Serious	Not serious	Not serious	Very serious	Undetected	19	19	1.30 (0.53,3.18)	Very low
**NRT - Varenicline**									
**9**	Very serious	Not serious	Not serious	Not serious	Undetected	1211	1390	0.71 (0.58,0.87)	Low
**NRT – Varenicline + NRT**									
**2**	Serious	Not serious	Not serious	Very serious	Undetected	122	122	0.54 (0.31,0.93)	Very low
**NRT + Mecamylamine - Placebo**									
**1**	Serious	Not serious	Not serious	Very serious	Undetected	20	20	6.22 (1.69,22.94)	Very low
**Naltrexone - Placebo**									
**9**	Very serious	Not serious	Not serious	Serious	Undetected	459	480	0.99 (0.64,1.54)	Very low
**Nortriptyline - Placebo**									
**2**	Serious	Not serious	Not serious	Serious	Undetected	513	532	1.87 (1.00,3.50)	Very low
**Placebo - Selegiline**									

№ of studies	Certainty assessment					№ of patients		Effect	Certainty
	Risk of bias	Inconsistency	Indirectness	Imprecision	Publication bias	Intervention-1	Intervention-2	OR with 95% CI	
**2**	Serious	Not serious	Not serious	Very serious	Undetected	70	71	0.87 (0.25,3.03)	Very low
**Placebo - Topiramate**									
**3**	Very serious	Not serious	Not serious	Very serious	Undetected	131	130	0.71 (0.29,1.73)	Very low
**Placebo - Varenicline**									
**49**	Serious	Not serious	Not serious	Not serious	Undetected	8461	9202	0.39 (0.33,0.45)	Moderate
**Placebo – Varenicline + Bupropion**									
**1**	Not serious	Not serious	Not serious	Serious	Undetected	56	166	0.29 (0.17,0.52)	Moderate
**Varenicline – Varenicline + Bupropion**									
**3**	Not serious	Not serious	Not serious	Serious	Undetected	680	661	1.32 (0.76,2.29)	Moderate
**Varenicline – Varenicline + NRT**									
**2**	Serious	Not serious	Not serious	Serious	Undetected	482	481	0.77 (0.45,1.31)	Very low
**Bupropion vs Clonidine**									
**-**	Not serious	Not serious	Serious	Serious	Undetected	-	-	1.48 (0.76,2.91)	Low
**Bupropion vs Cytisine**									
**-**	Not serious	Not serious	Serious	Not serious	Undetected	-	-	0.83 (0.47,1.45)	Moderate
**Bupropion vs Fluoxetine**									
**-**	Not serious	Serious	Very serious	Serious	Undetected	-	-	1.38 (0.57,3.33)	Very low
**Bupropion vs NRT+ Mecamylamine**									
**-**	Not serious	Not serious	Serious	Not serious	Undetected	-	-	0.27 (0.07,1.02)	Moderate
**Bupropion vs Naltrexone**									
**-**	Not serious	Not serious	Serious	Not serious	Undetected	-	-	1.72 (1.07,2.76)	Moderate
**Bupropion vs Selegiline**									
**-**	Not serious	Very serious	Serious	Serious	Undetected	-	-	1.49 (0.42,5.22)	Very low
**Bupropion vs Topiramate**									

№ of studies	Certainty assessment					№ of patients		Effect	Certainty
	Risk of bias	Inconsistency	Indirectness	Imprecision	Publication bias	Intervention-1	Intervention-2	OR with 95% CI	
-	Not serious	Serious	Serious	Not serious	Undetected	-	-	1.21 (0.49,2.99)	Low
**Bupropion vs Varenicline+ Bupropion**									
-	Not serious	Not serious	Very serious	Not serious	Undetected	-	-	0.76 (0.44,1.31)	Low
**Bupropion vs Varenicline+ NRT**									
-	Not serious	Not serious	Very serious	Not serious	Undetected	-	-	0.51 (0.29,0.89)	Low
**Bupropion+ NRT vs Clonidine**									
-	Not serious	Very serious	Serious	Not serious	Undetected	-	-	1.70 (0.83,3.50)	Very low
**Bupropion+ NRT vs Cytisine**									
-	Not serious	Not serious	Serious	Not serious	Undetected	-	-	0.95 (0.51,1.77)	Moderate
**Bupropion+ NRT vs Fluoxetine**									
--	Not serious	Serious	Serious	Not serious	Undetected	-	-	1.58 (0.63,3.97)	Low
**Bupropion+ NRT vs NRT+ Mecamylamine**									
-	Not serious	Serious	Very serious	Not serious	Undetected	-	-	0.31 (0.08,1.19)	Very low
**Bupropion+ NRT vs Naltrexone**									
-	Not serious	Not serious	Very serious	Serious	Undetected	-	-	1.97 (1.15,3.39)	Very low
**Bupropion+ NRT vs Nortriptyline**									
-	Not serious	Not serious	Serious	Not serious	Undetected	-	-	1.04 (0.52,2.09)	Moderate
**Bupropion+ NRT vs Selegiline**									
-	Not serious	Serious	Very serious	Serious	Undetected	-	-	1.71 (0.47,6.16)	Very low
**Bupropion+ NRT vs Topiramate**									
-	Not serious	Not serious	Serious	Not serious	Undetected	-	-	1.39 (0.55,3.56)	Moderate
**Bupropion+ NRT vs Varenicline**									
-	Not serious	Not serious	Very serious	Not serious	Undetected	-	-	0.76 (0.54,1.07)	Low

№ of studies	Certainty assessment					№ of patients		Effect	Certainty
	Risk of bias	Inconsistency	Indirectness	Imprecision	Publication bias	Intervention-1	Intervention-2	OR with 95% CI	
**Bupropion+ NRT vs Varenicline+ Bupropion**									
-	Not serious	Not serious	Serious	Not serious	Undetected	-	-	0.57 (0.30,1.09)	Low
**Bupropion+ NRT vs Varenicline+ NRT**									
-	Not serious	Not serious	Very serious	Not serious	Undetected	-	-	0.58 (0.31,1.08)	Very low
**Clonidine vs Cytisine**									
-	Not serious	Serious	Serious	Not serious	Undetected	-	-	0.56 (0.24,1.30)	Low
**Clonidine vs Fluoxetine**									
-	Not serious	Serious	Serious	Not serious	Undetected	-	-	0.93 (0.32,2.74)	Low
**Clonidine vs NRT+ Mecamylamine**									
-	Not serious	Not serious	Serious	Not serious	Undetected	-	-	0.18 (0.04,0.79)	Moderate
**Clonidine vs Nortriptyline**									
-	Not serious	Not serious	Serious	Not serious	Undetected	-	-	0.61 (0.25,1.51)	Low
**Clonidine vs Selegiline**									
-	Not serious	Serious	Serious	Serious	Undetected	-	-	1.00 (0.25,4.08)	Very low
**Clonidine vs Topiramate**									
-	Not serious	Not serious	Serious	Not serious	Undetected	-	-	0.82 (0.27,2.45)	Moderate
**Clonidine vs Varenicline**									
-	Not serious	Not serious	Serious	Not serious	Undetected	-	-	0.44 (0.23,0.87)	Moderate
**Clonidine vs Varenicline+ Bupropion**									
-	Not serious	Very serious	Serious	Not serious	Undetected	-	-	0.34 (0.14,0.80)	Very low
**Clonidine vs Varenicline+ NRT**									
-	Not serious	Not serious	Serious	Not serious	Undetected	-	-	0.34 (0.15,0.79)	Moderate
**Cytisine vs Fluoxetine**									
-	Not serious	Serious	Serious	Serious	Undetected	-	-	1.67 (0.60,4.61)	Very low

№ of studies	Certainty assessment					№ of patients		Effect	Certainty
	Risk of bias	Inconsistency	Indirectness	Imprecision	Publication bias	Intervention-1	Intervention-2	OR with 95% CI	
**Cytisine vs NRT**									
-	Not serious	Serious	Serious	Not serious	Undetected	-	-	1.13 (0.65,1.97)	Low
**Cytisine vs NRT+ Mecamylamine**									
-	Not serious	Not serious	Very serious	Not serious	Undetected	-	-	0.33 (0.08,1.35)	Low
**Cytisine vs Naltrexone**									
-	Not serious	Not serious	Very serious	Not serious	Undetected	-	-	2.08 (1.04,4.16)	Low
**Cytisine vs Nortriptyline**									
-	Not serious	Serious	Serious	Not serious	Undetected	-	-	1.10 (0.48,2.50)	Low
**Cytisine vs Selegiline**									
-	Not serious	Not serious	Very serious	Serious	Undetected	-	-	1.80 (0.46,6.97)	Very low
**Cytisine vs Topiramate**									
-	Not serious	Not serious	Serious	Serious	Undetected	-	-	1.47 (0.52,4.13)	Low
**Cytisine vs Varenicline+ Bupropion**									
-	Not serious	Very serious	Serious	Not serious	Undetected	-	-	0.60 (0.28,1.30)	Very low
**Cytisine vs Varenicline+ NRT**									
-	Not serious	Serious	Very serious	Not serious	Undetected	-	-	0.61 (0.29,1.30)	Very low
**Fluoxetine vs NRT**									
-	Not serious	Not serious	Serious	Not serious	Undetected	-	-	0.67 (0.28,1.62)	Moderate
**Fluoxetine vs NRT+ Mecamylamine**									
-	Not serious	Serious	Serious	Not serious	Undetected	-	-	0.20 (0.04,0.95)	Low
**Fluoxetine vs Naltrexone**									
-	Not serious	Not serious	Serious	Not serious	Undetected	-	-	1.24 (0.47,3.28)	Moderate
**Fluoxetine vs Nortriptyline**									

№ of studies	Certainty assessment					№ of patients		Effect	Certainty
	Risk of bias	Inconsistency	Indirectness	Imprecision	Publication bias	Intervention-1	Intervention-2	OR with 95% CI	
-	Not serious	Not serious	Serious	Serious	Undetected	-	-	0.66 (0.23,1.91)	Low
**Fluoxetine vs Selegiline**									
-	Not serious	Serious	Serious	Serious	Undetected	-	-	1.08 (0.24,4.89)	Very low
**Fluoxetine vs Topiramate**									
-	Not serious	Not serious	Serious	Not serious	Undetected	-	-	0.88 (0.26,3.02)	Moderate
**Fluoxetine vs Varenicline**									
-	Not serious	Not serious	Serious	Not serious	Undetected	-	-	0.48 (0.20,1.14)	Moderate
**Fluoxetine vs Varenicline+ Bupropion**									
-	Not serious	Very serious	Serious	Not serious	Undetected	-	-	0.36 (0.13,1.01)	Very low
**Fluoxetine vs Varenicline+ NRT**									
-	Not serious	Not serious	Serious	Not serious	Undetected	-	-	0.37 (0.13,1.01)	Moderate
**NRT vs Nortriptyline**									
-	Not serious	Serious	Serious	Not serious	Undetected	-	-	0.97 (0.51,1.86)	Low
**NRT vs Selegiline**									
-	Not serious	Serious	Very serious	Serious	Undetected	-	-	1.60 (0.45,5.59)	Very low
**NRT vs Varenicline + Bupropion**									
-	Not serious	Serious	Serious	Not serious	Undetected	-	-	0.54 (0.30,0.96)	Low
**NRT+ Mecamylamine vs Naltrexone**									
-	Not serious	Not serious	Serious	Very serious	Undetected	-	-	6.29 (1.59,24.90)	Very low
**NRT+ Mecamylamine vs Nortriptyline**									
-	Not serious	Serious	Serious	Very serious	Undetected	-	-	3.32 (0.78,14.12)	Very low
**NRT+ Mecamylamine vs Selegiline**									
-	Not serious	Not serious	Serious	Very serious	Undetected	-	-	5.44 (0.90,32.99)	Very low
**NRT+ Mecamylamine vs Topiramate**									

№ of studies	Certainty assessment					№ of patients		Effect	Certainty
	Risk of bias	Inconsistency	Indirectness	Imprecision	Publication bias	Intervention-1	Intervention-2	OR with 95% CI	
-	Not serious	Not serious	Serious	Very serious	Undetected	-	-	4.44 (0.92,21.44)	Very low
**NRT+ Mecamylamine vs Varenicline**									
-	Not serious	Serious	Very serious	Serious	Undetected	-	-	2.41 (0.65,8.95)	Very low
**NRT+ Mecamylamine vs Varenicline+ Bupropion**									
-	Not serious	Not serious	Serious	Serious	Undetected	-	-	1.83 (0.44,7.56)	Low
**NRT+ Mecamylamine vs Varenicline+ NRT**									
-	Not serious	Serious	Serious	Serious	Undetected	-	-	1.85 (0.45,7.55)	Very low
**Naltrexone vs Nortriptyline**									
-	Not serious	Serious	Very serious	Not serious	Undetected	-	-	0.53 (0.25,1.14)	Very low
**Naltrexone vs Selegiline**									
-	Not serious	Serious	Very serious	Not serious	Undetected	-	-	0.86 (0.23,3.24)	Very low
**Naltrexone vs Topiramate**									
-	Not serious	Serious	Very serious	Not serious	Undetected	-	-	0.71 (0.26,1.90)	Very low
**Naltrexone vs Varenicline**									
-	Not serious	Serious	Serious	Not serious	Undetected	-	-	0.38 (0.24,0.61)	Low
**Naltrexone vs Varenicline+ Bupropion**									
-	Not serious	Not serious	Very serious	Not serious	Undetected	-	-	0.29 (0.14,0.60)	Low
**Naltrexone vs Varenicline+ NRT**									
-	Not serious	Serious	Serious	Not serious	Undetected	-	-	0.29 (0.15,0.59)	Low
**Nortriptyline vs Selegiline**									
-	Not serious	Very serious	Serious	Serious	Undetected	-	-	1.64 (0.41,6.59)	Very low
**Nortriptyline vs Topiramate**									
-	Not serious	Not serious	Very serious	Not serious	Undetected	-	-	1.34 (0.45,3.95)	Low

№ of studies	Certainty assessment					№ of patients		Effect	Certainty
	Risk of bias	Inconsistency	Indirectness	Imprecision	Publication bias	Intervention-1	Intervention-2	OR with 95% CI	
**Nortriptyline vs Varenicline**									
-	Not serious	Serious	Serious	Not serious	Undetected	-	-	0.73 (0.38,1.38)	Low
**Nortriptyline vs Varenicline+ Bupropion**									
-	Not serious	Not serious	Very serious	Not serious	Undetected	-	-	0.55 (0.24,1.28)	Low
**Nortriptyline vs Varenicline+ NRT**									
-	Not serious	Not serious	Serious	Not serious	Undetected	-	-	0.56 (0.24,1.27)	Moderate
**Placebo vs Varenicline+ NRT**									
-	Not serious	Not serious	Serious	Not serious	Undetected	-	-	0.30 (0.17,0.51)	Moderate
**Selegiline vs Topiramate**									
-	Not serious	Serious	Very serious	Not serious	Undetected	-	-	0.82 (0.18,3.76)	Very low
**Selegiline vs Varenicline**									
-	Not serious	Not serious	Serious	Not serious	Undetected	-	-	0.44 (0.13,1.55)	Moderate
**Selegiline vs Varenicline+ Bupropion**									
-	Not serious	Very serious	Serious	Not serious	Undetected	-	-	0.34 (0.09,1.32)	Very low
**Selegiline vs Varenicline+ NRT**									
-	Not serious	Not serious	Serious	Not serious	Undetected	-	-	0.34 (0.09,1.32)	Moderate
**Topiramate vs Varenicline**									
-	Not serious	Not serious	Serious	Not serious	Undetected	-	-	1.84 (0.75,4.52)	Moderate
**Topiramate vs Varenicline+ Bupropion**									
-	Not serious	Serious	Serious	Not serious	Undetected	-	-	0.41 (0.14,1.18)	Low
**Topiramate vs Varenicline+ NRT**									
-	Not serious	Not serious	Serious	Not serious	Undetected	-	-	0.42 (0.15,1.17)	Moderate
**Varenicline+ Bupropion vs Varenicline+ NRT**									
-	Not serious	Not serious	Serious	Not serious	Undetected	-	-	1.01 (0.47,2.18)	Moderate

Note:
All included studies were randomized controlled trials.
OR: Odds Ratio; CI: Confidence interval; NRT: Nicotine replacement therapy

Reference list of included trials

1. King A, Vena A, de Wit H, Grant JE, Cao D. Effect of Combination Treatment With Varenicline and Nicotine Patch on Smoking Cessation Among Smokers Who Drink Heavily: A Randomized Clinical Trial. *JAMA network open.* 2022;5(3):e220951-e220951.
2. King A, de Wit H, Riley RC, Cao D, Niaura R, Hatsukami D. Efficacy of naltrexone in smoking cessation: a preliminary study and an examination of sex differences. *Nicotine & tobacco research : official journal of the Society for Research on Nicotine and Tobacco.* 2006;8(5):671-682.
3. Hughes JR, Novy P, Hatsukami DK, Jensen J, Callas PW. Efficacy of nicotine patch in smokers with a history of alcoholism. *Alcohol Clin Exp Res.* 2003;27(6):946-954.
4. Hoogsteder PH, Kotz D, van Spiegel PI, Viechtbauer W, van Schayck OC. Efficacy of the nicotine vaccine 3'-AmNic-rEPA (NicVAX) co-administered with varenicline and counselling for smoking cessation: a randomized placebo-controlled trial. *Addiction (Abingdon, England).* 2014;109(8):1252-1259.
5. Nakamura M, Abe M, Ohkura M, Treadow J, Yu CR, Park PW. Efficacy of Varenicline for Cigarette Reduction Before Quitting in Japanese Smokers: A Subpopulation Analysis of the Reduce to Quit Trial. *Clin Ther.* 2017;39(4):863-872.
6. Hughes JR, Rennard SI, Fingar JR, Talbot SK, Callas PW, Fagerstrom KO. Efficacy of varenicline to prompt quit attempts in smokers not currently trying to quit: a randomized placebo-controlled trial. *Nicotine & tobacco research : official journal of the Society for Research on Nicotine and Tobacco.* 2011;13(10):955-964.
7. Pfeifer P, Fehr C. Efficacy of Varenicline in Patients With Severe Alcohol Dependence: A Pilot Double-Blind Randomized and Controlled Study. *J Clin Psychopharmacol.* 2019;39(4):398-402.
8. Gray KM, McClure EA, Baker NL, Hartwell KJ, Carpenter MJ, Saladin ME. An exploratory short-term double-blind randomized trial of varenicline versus nicotine patch for smoking cessation in women. *Addiction (Abingdon, England).* 2015;110(6):1027-1034.
9. Bohadana A, Freier-Dror Y, Peles V, Babai P, Izbicki G. Extending varenicline preloading to 6 weeks facilitates smoking cessation: A single-site, randomised controlled trial. *EClinicalMedicine.* 2020;19:100228.
10. Sun HQ, Guo S, Chen DF, et al. Family support and employment as predictors of smoking cessation success: a randomized, double-blind, placebo-controlled trial of nicotine sublingual tablets in chinese smokers. *The American journal of drug and alcohol abuse.* 2009;35(3):183-188.
11. Gray KM, Rubinstein ML, Prochaska JJ, et al. High-dose and low-dose varenicline for smoking cessation in adolescents: a randomised, placebo-controlled trial. *The Lancet Child & Adolescent Health.* 2020;4(11):837-845.
12. Kalman D, Herz L, Monti P, et al. Incremental efficacy of adding bupropion to the nicotine patch for smoking cessation in smokers with a recent history of alcohol dependence: results from a randomized, double-blind, placebo-controlled study. *Drug and alcohol dependence.* 2011;118(2-3):111-118.
13. Jamerson BD, Nides M, Jorenby DE, et al. Late-term smoking cessation despite initial failure: an evaluation of bupropion sustained release, nicotine patch, combination therapy, and placebo. *Clin Ther.* 2001;23(5):744-752.
14. Scherphof CS, van den Eijnden RJ, Engels RC, Vollebergh WA. Long-term efficacy of nicotine replacement therapy for smoking cessation in adolescents: a randomized controlled trial. *Drug*

and alcohol dependence. 2014;140:217-220.
15. Toll BA, White M, Wu R, et al. Low-dose naltrexone augmentation of nicotine replacement for smoking cessation with reduced weight gain: a randomized trial. *Drug and alcohol dependence.* 2010;111(3):200-206.
16. Nides M, Rigotti NA, Benowitz N, Clarke A, Jacobs C. A Multicenter, Double-Blind, Randomized, Placebo-Controlled Phase 2b Trial of Cytisinicline in Adult Smokers (The ORCA-1 Trial). *Nicotine & tobacco research : official journal of the Society for Research on Nicotine and Tobacco.* 2021;23(10):1656-1663.
17. Dalsgareth OJ, Hansen NC, Soes-Petersen U, et al. A multicenter, randomized, double-blind, placebo-controlled, 6-month trial of bupropion hydrochloride sustained-release tablets as an aid to smoking cessation in hospital employees. *Nicotine & tobacco research : official journal of the Society for Research on Nicotine and Tobacco.* 2004;6(1):55-61.
18. Tønnesen P, Tonstad S, Hjalmarson A, et al. A multicentre, randomized, double-blind, placebo-controlled, 1-year study of bupropion SR for smoking cessation. *J Intern Med.* 2003;254(2):184-192.
19. Ebbert JO, Hatsukami DK, Croghan IT, et al. Combination Varenicline and Bupropion SR for Tobacco-Dependence Treatment in Cigarette Smokers A Randomized Trial. *Jama-Journal of the American Medical Association.* 2014;311(2):155-163.
20. Covey LS, Glassman AH, Stetner F. Naltrexone effects on short-term and long-term smoking cessation. *J Addict Dis.* 1999;18(1):31-40.
21. Oncken C, Dornelas EA, Kuo CL, et al. Randomized Trial of Nicotine Inhaler for Pregnant Smokers. *Am J Obstet Gynecol MFM.* 2019;1(1):10-18.
22. Oncken C, Dornelas E, Greene J, et al. Nicotine gum for pregnant smokers: a randomized controlled trial. *Obstetrics and gynecology.* 2008;112(4):859-867.
23. Caldwell BO, Crane J. Combination Nicotine Metered Dose Inhaler and Nicotine Patch for Smoking Cessation: A Randomized Controlled Trial. *Nicotine & Tobacco Research.* 2016;18(10):1944-1951.
24. Bohadana A, Nilsson F, Rasmussen T, Martinet Y. Nicotine inhaler and nicotine patch as a combination therapy for smoking cessation: a randomized, double-blind, placebo-controlled trial. *Archives of internal medicine.* 2000;160(20):3128-3134.
25. Hjalmarson A, Nilsson F, Sjostrom L, Wiklund O. The nicotine inhaler in smoking cessation. *Archives of internal medicine.* 1997;157(15):1721-1728.
26. Hurt RD, Dale LC, Fredrickson PA, et al. Nicotine Patch Therapy for SmokingCessation Combined With PhysicianAdvice and Nurse Follow-up One-Year Outcome and Percentage of Nicotine Replacement. *Jama.* 1994;271(8):595-600.
27. Nicotine replacement therapy for patients with coronary artery disease. Working Group for the Study of Transdermal Nicotine in Patients with Coronary artery disease. *Archives of internal medicine.* 1994;154(9):989-995.
28. Etter JF, Laszlo E, Zellweger JP, Perrot C, Perneger TV. Nicotine replacement to reduce cigarette consumption in smokers who are unwilling to quit: a randomized trial. *J Clin Psychopharmacol.* 2002;22(5):487-495.
29. Rose JE, Behm FM, Westman EC. Nicotine-mecamylamine treatment for smoking cessation: the role of pre-cessation therapy. *Experimental and clinical psychopharmacology.* 1998;6(3):331-343.
30. Aveyard P, Johnson C, Fillingham S, Parsons A, Murphy M. Nortriptyline plus nicotine replacement versus placebo plus nicotine replacement for smoking cessation: pragmatic randomised controlled trial. *BMJ (Clinical research ed).* 2008;336(7655):1223-1227.

31. Wong J, Abrishami A, Yang Y, et al. A perioperative smoking cessation intervention with varenicline: a double-blind, randomized, placebo-controlled trial. *Anesthesiology.* 2012;117(4):755-764.
32. Ebbert JO, Croghan IT, Severson HH, Schroeder DR, Hays JT. A pilot study of the efficacy of varenicline for the treatment of smokeless tobacco users in Midwestern United States. *Nicotine & tobacco research : official journal of the Society for Research on Nicotine and Tobacco.* 2011;13(9):820-826.
33. Cooper TV, Klesges RC, Debon MW, Zbikowski SM, Johnson KC, Clemens LH. A placebo controlled randomized trial of the effects of phenylpropanolamine and nicotine gum on cessation rates and postcessation weight gain in women. *Addictive behaviors.* 2005;30(1):61-75.
34. George TP, Vessicchio JC, Termine A, Jatlow PI, Kosten TR, O'Malley SS. A preliminary placebo-controlled trial of selegiline hydrochloride for smoking cessation. *Biological psychiatry.* 2003;53(2):136-143.
35. Etter J-F, Laszlo E. Postintervention effect of nicotine replacement therapy for smoking reduction - A randomized trial with a 5-year follow-up. *Journal of Clinical Psychopharmacology.* 2007;27(2):151-155.
36. Etter JF, Laszlo E, Perneger TV. Postintervention effect of nicotine replacement therapy on smoking reduction in smokers who are unwilling to quit: Randomized trial. *Journal of Clinical Psychopharmacology.* 2004;24(2):174-179.
37. George TP, Vessicchio JC, Termine A, Jatlow PI, Kosten TR, O'Malley SS. A preliminary placebo-controlled trial of selegiline hydrochloride for smoking cessation. *Biological psychiatry.* 2003;53(2):136-143.
38. Hertzberg MA, Moore SD, Feldman ME, Beckham JC. A preliminary study of bupropion sustained-release for smoking cessation in patients with chronic posttraumatic stress disorder. *J Clin Psychopharmacol.* 2001;21(1):94-98.
39. Killen JD, Robinson TN, Ammerman S, et al. Randomized clinical trial of the efficacy of bupropion combined with nicotine patch in the treatment of adolescent smokers. *Journal of consulting and clinical psychology.* 2004;72(4):729-735.
40. Croghan IT, Hurt RD, Dakhil SR, et al. Randomized comparison of a nicotine inhaler and bupropion for smoking cessation and relapse prevention. *Mayo Clin Proc.* 2007;82(2):186-195.
41. Tsukahara H, Noda K, Saku K. A randomized controlled open comparative trial of varenicline vs nicotine patch in adult smokers: efficacy, safety and withdrawal symptoms (the VN-SEESAW study). *Circulation journal : official journal of the Japanese Circulation Society.* 2010;74(4):771-778.
42. Schnoll R, Leone F, Veluz-Wilkins A, et al. A randomized controlled trial of 24 weeks of varenicline for tobacco use among cancer patients: Efficacy, safety, and adherence. *Psycho-oncology.* 2019;28(3):561-569.
43. Piper ME, Smith SS, Schlam TR, et al. A randomized placebo-controlled clinical trial of 5 smoking cessation pharmacotherapies. *Arch Gen Psychiatry.* 2009;66(11):1253-1262.
44. Rennard S, Hughes J, Cinciripini PM, et al. A randomized placebo-controlled trial of varenicline for smoking cessation allowing flexible quit dates. *Nicotine & tobacco research : official journal of the Society for Research on Nicotine and Tobacco.* 2012;14(3):343-350.
45. Wong GY, Wolter TD, Croghan GA, Croghan IT, Offord KP, Hurt RD. A randomized trial of naltrexone for smoking cessation. *Addiction (Abingdon, England).* 1999;94(8):1227-1237.
46. Stapleton J, West R, Hajek P, et al. Randomized trial of nicotine replacement therapy (NRT), bupropion and NRT plus bupropion for smoking cessation: effectiveness in clinical practice. *Addiction (Abingdon, England).* 2013;108(12):2193-2201.

47. Evins AE, Cather C, Culhane MA, et al. A 12-week double-blind, placebo-controlled study of bupropion sr added to high-dose dual nicotine replacement therapy for smoking cessation or reduction in schizophrenia. *J Clin Psychopharmacol.* 2007;27(4):380-386.
48. Hays JT, Hurt RD, Decker PA, Croghan IT, Offord KP, Patten CA. A randomized, controlled trial of bupropion sustained-release for preventing tobacco relapse in recovering alcoholics. *Nicotine & tobacco research : official journal of the Society for Research on Nicotine and Tobacco.* 2009;11(7):859-867.
49. Franks P, Harp J, Bell B. Randomized, controlled trial of clonidine for smoking cessation in a primary care setting. *Jama.* 1989;262(21):3011-3013.
50. Glover ED, Laflin MT, Schuh KJ, et al. A randomized, controlled trial to assess the efficacy and safety of a transdermal delivery system of nicotine/mecamylamine in cigarette smokers. *Addiction (Abingdon, England).* 2007;102(5):795-802.
51. Williams JM, Anthenelli RM, Morris CD, et al. A randomized, double-blind, placebo-controlled study evaluating the safety and efficacy of varenicline for smoking cessation in patients with schizophrenia or schizoaffective disorder. *The Journal of clinical psychiatry.* 2012;73(5):654-660.
52. Cinciripini PM, Minnix JA, Green CE, et al. An RCT with the combination of varenicline and bupropion for smoking cessation: clinical implications for front line use. *Addiction (Abingdon, England).* 2018.
53. Zawertailo L, Ivanova A, Ng G, Le Foll B, Selby P. Safety and Efficacy of Varenicline for Smoking Cessation in Alcohol-Dependent Smokers in Concurrent Treatment for Alcohol Use Disorder: A Pilot, Randomized Placebo-Controlled Trial. *J Clin Psychopharmacol.* 2020;40(2):130-136.
54. Poling J, Rounsaville B, Gonsai K, Severino K, Sofuoglu M. The safety and efficacy of varenicline in cocaine using smokers maintained on methadone: a pilot study. *The American journal on addictions.* 2010;19(5):401-408.
55. Scherphof CS, van den Eijnden RJ, Engels RC, Vollebergh WA. Short-term efficacy of nicotine replacement therapy for smoking cessation in adolescents: a randomized controlled trial. *Journal of substance abuse treatment.* 2014;46(2):120-127.
56. Windle SB, Dehghani P, Roy N, et al. Smoking abstinence 1 year after acute coronary syndrome: follow-up from a randomized controlled trial of varenicline in patients admitted to hospital. *CMAJ : Canadian Medical Association journal = journal de l'Association medicale canadienne.* 2018;190(12):E347-e354.
57. Cooney NL, Cooney JL, Perry BL, et al. Smoking cessation during alcohol treatment: a randomized trial of combination nicotine patch plus nicotine gum. *Addiction (Abingdon, England).* 2009;104(9):1588-1596.
58. Tonstad S. Smoking cessation efficacy and safety of varenicline, an alpha4beta2 nicotinic receptor partial agonist. *The Journal of cardiovascular nursing.* 2006;21(6):433-436.
59. Kralikova E, Kozak JT, Rasmussen T, Gustavsson G, Le Houezec J. Smoking cessation or reduction with nicotine replacement therapy: a placebo-controlled double blind trial with nicotine gum and inhaler. *BMC public health.* 2009;9:433.
60. Nides M, Oncken C, Gonzales D, et al. Smoking cessation with varenicline, a selective alpha4beta2 nicotinic receptor partial agonist: results from a 7-week, randomized, placebo- and bupropion-controlled trial with 1-year follow-up. *Archives of internal medicine.* 2006;166(15):1561-1568.
61. Glavas D, Rumboldt M, Rumboldt Z. Smoking cessation with nicotine replacement therapy among health care workers: randomized double-blind study. *Croat Med J.* 2003;44(2):219-224.

62. Cooper S, Lewis S, Thornton JG, et al. The SNAP trial: a randomised placebo-controlled trial of nicotine replacement therapy in pregnancy--clinical effectiveness and safety until 2 years after delivery, with economic evaluation. *Health technology assessment (Winchester, England).* 2014;18(54):1-128.
63. Fagerström K, Gilljam H, Metcalfe M, Tonstad S, Messig M. Stopping smokeless tobacco with varenicline: randomised double blind placebo controlled trial. *BMJ (Clinical research ed).* 2010;341:c6549.
64. da Costa CL, Younes RN, Lourenço MT. Stopping smoking: a prospective, randomized, double-blind study comparing nortriptyline to placebo. *Chest.* 2002;122(2):403-408.
65. Dennis PA, Kimbrel NA, Dedert EA, Beckham JC, Dennis MF, Calhoun PS. Supplemental nicotine preloading for smoking cessation in posttraumatic stress disorder: Results from a randomized controlled trial. *Addictive behaviors.* 2016;59:24-29.
66. Hays JT, Hurt RD, Rigotti NA, et al. Sustained-release bupropion for pharmacologic relapse prevention after smoking cessation. a randomized, controlled trial. *Annals of internal medicine.* 2001;135(6):423-433.
67. Steinberg MB, Randall J, Greenhaus S, Schmelzer AC, Richardson DL, Carson JL. Tobacco dependence treatment for hospitalized smokers: a randomized, controlled, pilot trial using varenicline. *Addictive behaviors.* 2011;36(12):1127-1132.
68. Oncken C, Arias AJ, Feinn R, et al. Topiramate for Smoking Cessation: A Randomized, Placebo-Controlled Pilot Study. *Nicotine & Tobacco Research.* 2014;16(3):288-296.
69. Prochazka AV, Petty TL, Nett L, et al. Transdermal clonidine reduced some withdrawal symptoms but did not increase smoking cessation. *Archives of internal medicine.* 1992;152(10):2065-2069.
70. Stapleton JA, Sutherland G. Treating heavy smokers in primary care with the nicotine nasal spray: randomized placebo-controlled trial. *Addiction (Abingdon, England).* 2011;106(4):824-832.
71. Steinberg MB, Greenhaus S, Schmelzer AC, et al. Triple-combination pharmacotherapy for medically ill smokers: a randomized trial. *Annals of internal medicine.* 2009;150(7):447-454.
72. Ahmadi J, Ashkani H, Ahmadi M, Ahmadi N. Twenty-four week maintenance treatment of cigarette smoking with nicotine gum, clonidine and naltrexone. *Journal of substance abuse treatment.* 2003;24(3):251-255.
73. Roddy E, Romilly N, Challenger A, Lewis S, Britton J. Use of nicotine replacement therapy in socioeconomically deprived young smokers: a community-based pilot randomised controlled trial. *Tobacco control.* 2006;15(5):373-376.
74. Johnson BA, Ait-Daoud N, Akhtar FZ, Javors MA. Use of oral topiramate to promote smoking abstinence among alcohol-dependent smokers: a randomized controlled trial. *Archives of internal medicine.* 2005;165(14):1600-1605.
75. Hajek P, McRobbie HJ, Myers KE, Stapleton J, Dhanji AR. Use of varenicline for 4 weeks before quitting smoking: decrease in ad lib smoking and increase in smoking cessation rates. *Archives of internal medicine.* 2011;171(8):770-777.
76. Piper ME, Federmen EB, McCarthy DE, et al. Using mediational models to explore the nature of tobacco motivation and tobacco treatment effects. *J Abnorm Psychol.* 2008;117(1):94-105.
77. Tuisku A, Salmela M, Nieminen P, Toljamo T. Varenicline and Nicotine Patch Therapies in Young Adults Motivated to Quit Smoking: A Randomized, Placebo-controlled, Prospective Study. *Basic Clin Pharmacol Toxicol.* 2016;119(1):78-84.
78. Herrmann ES, Cooper ZD, Bedi G, et al. Varenicline and nabilone in tobacco and cannabis co-users: effects on tobacco abstinence, withdrawal and a laboratory model of cannabis relapse. *Addiction biology.* 2019;24(4):765-776.

79. Stein MD, Caviness CM, Kurth ME, Audet D, Olson J, Anderson BJ. Varenicline for smoking cessation among methadone-maintained smokers: a randomized clinical trial. *Drug and alcohol dependence.* 2013;133(2):486-493.
80. Ebbert JO, Croghan IT, Hurt RT, Schroeder DR, Hays JT. Varenicline for Smoking Cessation in Light Smokers. *Nicotine & tobacco research : official journal of the Society for Research on Nicotine and Tobacco.* 2016;18(10):2031-2035.
81. Antoniu SA, Trofor AC. Varenicline for smoking cessation intervention in chronic obstructive pulmonary disease. *Expert Opin Pharmacother.* 2011;12(16):2595-2597.
82. Wang C, Xiao D, Chan KP, Pothirat C, Garza D, Davies S. Varenicline for smoking cessation: a placebo-controlled, randomized study. *Respirology (Carlton, Vic).* 2009;14(3):384-392.
83. Hurt RT, Ebbert JO, Croghan IT, Schroeder DR, Hurt RD, Hays JT. Varenicline for tobacco-dependence treatment in alcohol-dependent smokers: A randomized controlled trial. *Drug and alcohol dependence.* 2018;184:12-17.
84. Hajek P, Tønnesen P, Arteaga C, Russ C, Tonstad S. Varenicline in prevention of relapse to smoking: effect of quit pattern on response to extended treatment. *Addiction (Abingdon, England).* 2009;104(9):1597-1602.
85. Nides M, Glover ED, Reus VI, et al. Varenicline versus bupropion SR or placebo for smoking cessation: a pooled analysis. *American journal of health behavior.* 2008;32(6):664-675.
86. Gray KM, Carpenter MJ, Lewis AL, Klintworth EM, Upadhyaya HP. Varenicline versus bupropion XL for smoking cessation in older adolescents: a randomized, double-blind pilot trial. *Nicotine & tobacco research : official journal of the Society for Research on Nicotine and Tobacco.* 2012;14(2):234-239.
87. Rohsenow DJ, Tidey JW, Martin RA, et al. Varenicline versus nicotine patch with brief advice for smokers with substance use disorders with or without depression: effects on smoking, substance use and depressive symptoms. *Addiction (Abingdon, England).* 2017;112(10):1808-1820.
88. Dogar O, Zahid R, Mansoor S, et al. Varenicline versus placebo for waterpipe smoking cessation: a double-blind randomized controlled trial. *Addiction (Abingdon, England).* 2018;113(12):2290-2299.
89. Aubin HJ, Bobak A, Britton JR, et al. Varenicline versus transdermal nicotine patch for smoking cessation: results from a randomised open-label trial. *Thorax.* 2008;63(8):717-724.
90. Bolt DM, Piper ME, Theobald WE, Baker TB. Why two smoking cessation agents work better than one: role of craving suppression. *Journal of consulting and clinical psychology.* 2012;80(1):54-65.
91. Yudkin PL, Jones L, Lancaster T, Fowler GH. Which smokers are helped to give up smoking using transdermal nicotine patches? Results from a randomized, double-blind, placebo-controlled trial. *The British journal of general practice : the journal of the Royal College of General Practitioners.* 1996;46(404):145-148.
92. Gonzales D, Rennard SI, Nides M, et al. Varenicline, an alpha4beta2 nicotinic acetylcholine receptor partial agonist, vs sustained-release bupropion and placebo for smoking cessation: a randomized controlled trial. *Jama.* 2006;296(1):47-55.
93. Fagerström K, Nakamura M, Cho HJ, et al. Varenicline treatment for smoking cessation in Asian populations: a pooled analysis of placebo-controlled trials conducted in six Asian countries. *Current medical research and opinion.* 2010;26(9):2165-2173.
94. Eisenberg MJ, Grandi SM, Gervais A, et al. Bupropion for smoking cessation in patients hospitalized with acute myocardial infarction: a randomized, placebo-controlled trial. *Journal of the American College of Cardiology.* 2013;61(5):524-532.

95. Cox LS, Patten CA, Niaura RS, et al. Efficacy of bupropion for relapse prevention in smokers with and without a past history of major depression. *Journal of general internal medicine.* 2004;19(8):828-834.
96. Aubin HJ, Lebargy F, Berlin I, Bidaut-Mazel C, Chemali-Hudry J, Lagrue G. Efficacy of bupropion and predictors of successful outcome in a sample of French smokers: a randomized placebo-controlled trial. *Addiction (Abingdon, England).* 2004;99(9):1206-1218.
97. Wagena EJ, Knipschild PG, Huibers MJ, Wouters EF, van Schayck CP. Efficacy of bupropion and nortriptyline for smoking cessation among people at risk for or with chronic obstructive pulmonary disease. *Archives of internal medicine.* 2005;165(19):2286-2292.
98. Piper ME, Federman EB, McCarthy DE, et al. Efficacy of bupropion alone and in combination with nicotine gum. *Nicotine & tobacco research : official journal of the Society for Research on Nicotine and Tobacco.* 2007;9(9):947-954.
99. Schneider NG, Olmstead R, Mody FV, et al. Efficacy of a nicotine nasal spray in smoking cessation: a placebo-controlled, double-blind trial. *Addiction (Abingdon, England).* 1995;90(12):1671-1682.
100. Shiffman S, Dresler CM, Hajek P, Gilburt SJ, Targett DA, Strahs KR. Efficacy of a nicotine lozenge for smoking cessation. *Archives of internal medicine.* 2002;162(11):1267-1276.
101. Nakamura M, Oshima A, Fujimoto Y, Maruyama N, Ishibashi T, Reeves KR. Efficacy and tolerability of varenicline, an alpha4beta2 nicotinic acetylcholine receptor partial agonist, in a 12-week, randomized, placebo-controlled, dose-response study with 40-week follow-up for smoking cessation in Japanese smokers. *Clin Ther.* 2007;29(6):1040-1056.
102. Niaura R, Hays JT, Jorenby DE, et al. The efficacy and safety of varenicline for smoking cessation using a flexible dosing strategy in adult smokers: a randomized controlled trial. *Current medical research and opinion.* 2008;24(7):1931-1941.
103. Mercié P, Arsandaux J, Katlama C, et al. Efficacy and safety of varenicline for smoking cessation in people living with HIV in France (ANRS 144 Inter-ACTIV): a randomised controlled phase 3 clinical trial. *Lancet HIV.* 2018;5(3):e126-e135.
104. Rigotti NA, Pipe AL, Benowitz NL, Arteaga C, Garza D, Tonstad S. Efficacy and safety of varenicline for smoking cessation in patients with cardiovascular disease: a randomized trial. *Circulation.* 2010;121(2):221-229.
105. Oncken C, Gonzales D, Nides M, et al. Efficacy and safety of the novel selective nicotinic acetylcholine receptor partial agonist, varenicline, for smoking cessation. *Archives of internal medicine.* 2006;166(15):1571-1577.
106. Ayers CR, Heffner JL, Russ C, et al. Efficacy and safety of pharmacotherapies for smoking cessation in anxiety disorders: Subgroup analysis of the randomized, active- and placebo-controlled EAGLES trial. *Depress Anxiety.* 2020;37(3):247-260.
107. Nides M, Danielsson T, Saunders F, et al. Efficacy and Safety of a Nicotine Mouth Spray for Smoking Cessation: A Randomized, Multicenter, Controlled Study in a Naturalistic Setting. *Nicotine & tobacco research : official journal of the Society for Research on Nicotine and Tobacco.* 2020;22(3):339-345.
108. Spring B, Wurtman J, Wurtman R, et al. Efficacies of dexfenfluramine and fluoxetine in preventing weight gain after smoking cessation. *Am J Clin Nutr.* 1995;62(6):1181-1187.
109. Doran N, Dubrava S, Anthenelli RM. Effects of Varenicline, Depressive Symptoms, and Region of Enrollment on Smoking Cessation in Depressed Smokers. *Nicotine & tobacco research : official journal of the Society for Research on Nicotine and Tobacco.* 2019;21(2):156-162.
110. Murphy CM, MacKillop J, Martin RA, Tidey JW, Colby SM, Rohsenow DJ. Effects of varenicline versus transdermal nicotine replacement therapy on cigarette demand on quit day in individuals with substance use disorders. *Psychopharmacology.* 2017;234(16):2443-2452.

111. Tashkin DP, Rennard S, Hays JT, Ma W, Lawrence D, Lee TC. Effects of varenicline on smoking cessation in patients with mild to moderate COPD: a randomized controlled trial. *Chest.* 2011;139(3):591-599.
112. Anthenelli RM, Morris C, Ramey TS, et al. Effects of varenicline on smoking cessation in adults with stably treated current or past major depression: a randomized trial. *Annals of internal medicine.* 2013;159(6):390-400.
113. Baker TB, Piper ME, Stein JH, et al. Effects of Nicotine Patch vs Varenicline vs Combination Nicotine Replacement Therapy on Smoking Cessation at 26 Weeks: A Randomized Clinical Trial. *Jama.* 2016;315(4):371-379.
114. Blondal T, Gudmundsson LJ, Tomasson K, et al. The effects of fluoxetine combined with nicotine inhalers in smoking cessation--a randomized trial. *Addiction (Abingdon, England).* 1999;94(7):1007-1015.
115. Baker TB, Piper ME, Smith SS, Bolt DM, Stein JH, Fiore MC. Effects of Combined Varenicline With Nicotine Patch and of Extended Treatment Duration on Smoking Cessation: A Randomized Clinical Trial. *Jama.* 2021;326(15):1485-1493.
116. Sachs DP. Effectiveness of the 4-mg dose of nicotine polacrilex for the initial treatment of high-dependent smokers. *Archives of internal medicine.* 1995;155(18):1973-1980.
117. Shiffman S, Di Marino ME, Pillitteri JL. The effectiveness of nicotine patch and nicotine lozenge in very heavy smokers. *Journal of substance abuse treatment.* 2005;28(1):49-55.
118. Ebbert JO, Hughes JR, West RJ, et al. Effect of Varenicline on Smoking Cessation Through Smoking Reduction A Randomized Clinical Trial. *Jama-Journal of the American Medical Association.* 2015;313(7):687-694.
119. Westergaard CG, Porsbjerg C, Backer V. The effect of Varenicline on smoking cessation in a group of young asthma patients. *Respiratory medicine.* 2015;109(11):1416-1422.
120. Hjalmarson A, Franzon M, Westin A, Wiklund O. Effect of nicotine nasal spray on smoking cessation. A randomized, placebo-controlled, double-blind study. *Archives of internal medicine.* 1994;154(22):2567-2572.
121. Tonstad S, Tønnesen P, Hajek P, Williams KE, Billing CB, Reeves KR. Effect of maintenance therapy with varenicline on smoking cessation: a randomized controlled trial. *Jama.* 2006;296(1):64-71.
122. Courtney RJ, Tutka P, Farrell M. The Effect of Cytisine vs Varenicline on Smoking Cessation-Reply. *Jama.* 2021;326(18):1872-1873.
123. King A, Vena A, de Wit H, Grant JE, Cao D. Effect of Combination Treatment With Varenicline and Nicotine Patch on Smoking Cessation Among Smokers Who Drink Heavily: A Randomized Clinical Trial. *JAMA Netw Open.* 2022;5(3):e220951.
124. Fossati R, Apolone G, Negri E, et al. A double-blind, placebo-controlled, randomized trial of bupropion for smoking cessation in primary care. *Archives of internal medicine.* 2007;167(16):1791-1797.
125. Weinberger AH, Reutenauer EL, Jatlow PI, O'Malley SS, Potenza MN, George TP. A double-blind, placebo-controlled, randomized clinical trial of oral selegiline hydrochloride for smoking cessation in nicotine-dependent cigarette smokers. *Drug and alcohol dependence.* 2010;107(2-3):188-195.
126. Williams KE, Reeves KR, Billing CB, Jr., Pennington AM, Gong J. A double-blind study evaluating the long-term safety of varenicline for smoking cessation. *Current medical research and opinion.* 2007;23(4):793-801.
127. Kahler CW, Cioe PA, Tzilos GK, et al. A Double-Blind Randomized Placebo-Controlled Trial of Oral Naltrexone for Heavy-Drinking Smokers Seeking Smoking Cessation Treatment.

Alcohol Clin Exp Res. 2017;41(6):1201-1211.
128. Garza D, Murphy M, Tseng LJ, Riordan HJ, Chatterjee A. A double-blind randomized placebo-controlled pilot study of neuropsychiatric adverse events in abstinent smokers treated with varenicline or placebo. *Biological psychiatry.* 2011;69(11):1075-1082.
129. Evins AE, Cather C, Deckersbach T, et al. A double-blind placebo-controlled trial of bupropion sustained-release for smoking cessation in schizophrenia. *Journal of Clinical Psychopharmacology.* 2005;25(3):218-225.
130. Dogar O, Keding A, Gabe R, et al. Cytisine for smoking cessation in patients with tuberculosis: a multicentre, randomised, double-blind, placebo-controlled phase 3 trial. *The Lancet Global health.* 2020;8(11):e1408-e1417.
131. O'Malley SS, Cooney JL, Krishnan-Sarin S, et al. A controlled trial of naltrexone augmentation of nicotine replacement therapy for smoking cessation. *Archives of internal medicine.* 2006;166(6):667-674.
132. Hurt RD, Sachs DP, Glover ED, et al. A comparison of sustained-release bupropion and placebo for smoking cessation. *The New England journal of medicine.* 1997;337(17):1195-1202.
133. Ramon JM, Morchon S, Baena A, Masuet-Aumatell C. Combining varenicline and nicotine patches: a randomized controlled trial study in smoking cessation. *BMC medicine.* 2014;12:172.
134. Ebbert JO, Hatsukami DK, Croghan IT, et al. Combination varenicline and bupropion SR for tobacco-dependence treatment in cigarette smokers: a randomized trial. *Jama.* 2014;311(2):155-163.
135. Caldwell BO, Adamson SJ, Crane J. Combination Rapid-Acting Nicotine Mouth Spray and Nicotine Patch Therapy in Smoking Cessation. *Nicotine & Tobacco Research.* 2014;16(10):1356-1364.
136. Caldwell BO, Crane J. Combination Nicotine Metered Dose Inhaler and Nicotine Patch for Smoking Cessation: A Randomized Controlled Trial. *Nicotine & tobacco research : official journal of the Society for Research on Nicotine and Tobacco.* 2016;18(10):1944-1951.
137. Nanovskaya TN, Oncken C, Fokina VM, et al. Bupropion sustained release for pregnant smokers: a randomized, placebo-controlled trial. *Am J Obstet Gynecol.* 2017;216(4):420.e421-420.e429.
138. Dale LC, Ebbert JO, Glover ED, et al. Bupropion SR for the treatment of smokeless tobacco use. *Drug and alcohol dependence.* 2007;90(1):56-63.
139. Tonstad S, Farsang C, Klaene G, et al. Bupropion SR for smoking cessation in smokers with cardiovascular disease: a multicentre, randomised study. *European heart journal.* 2003;24(10):946-955.
140. Gonzales DH, Nides MA, Ferry LH, et al. Bupropion SR as an aid to smoking cessation in smokers treated previously with bupropion: a randomized placebo-controlled study. *Clinical pharmacology and therapeutics.* 2001;69(6):438-444.
141. Schnoll RA, Martinez E, Tatum KL, et al. A bupropion smoking cessation clinical trial for cancer patients. *Cancer causes & control : CCC.* 2010;21(6):811-820.
142. Planer D, Lev I, Elitzur Y, et al. Bupropion for smoking cessation in patients with acute coronary syndrome. *Archives of internal medicine.* 2011;171(12):1055-1060.
143. Cox LS, Nollen NL, Mayo MS, et al. Bupropion for smoking cessation in African American light smokers: a randomized controlled trial. *Journal of the National Cancer Institute.* 2012;104(4):290-298.
144. Simon JA, Duncan C, Carmody TP, Hudes ES. Bupropion for Smoking Cessation A Randomized Trial. *Archives of internal medicine.* 2004;164:1797-1803.
145. Rigotti NA, Thorndike AN, Regan S, et al. Bupropion for smokers hospitalized with acute cardiovascular disease. *The American journal of medicine.* 2006;119(12):1080-1087.

146. Weinberger AH, Vessicchio JC, Sacco KA, Creeden CL, Chengappa KN, George TP. A preliminary study of sustained-release bupropion for smoking cessation in bipolar disorder. *J Clin Psychopharmacol.* 2008;28(5):584-587.
147. Weiner E, Buchholz A, Coffay A, et al. Varenicline for smoking cessation in people with schizophrenia: a double blind randomized pilot study. *Schizophr Res.* 2011;129(1):94-95.
148. George TP, Vessicchio JC, Sacco KA, et al. A placebo-controlled trial of bupropion combined with nicotine patch for smoking cessation in schizophrenia. *Biological psychiatry.* 2008;63(11):1092-1096.
149. Le Mao R, Tromeur C, Paleiron N, et al. Effect of Early Initiation of Varenicline on Smoking Cessation in COPD Patients Admitted for Exacerbation: The Save Randomized Clinical Trial. *Copd.* 2020;17(1):7-14.
150. Qin R, Liu Z, Zhou X, et al. Adherence and Efficacy of Smoking Cessation Treatment Among Patients with COPD in China. *International journal of chronic obstructive pulmonary disease.* 2021;16:1203-1214.
151. Fridberg DJ, Cao D, Grant JE, King AC. Naltrexone improves quit rates, attenuates smoking urge, and reduces alcohol use in heavy drinking smokers attempting to quit smoking. *Alcohol Clin Exp Res.* 2014;38(10):2622-2629.
152. King A, Cao D, Vanier C, Wilcox T. Naltrexone decreases heavy drinking rates in smoking cessation treatment: an exploratory study. *Alcohol Clin Exp Res.* 2009;33(6):1044-1050.
153. Fucito LM, Toll BA, Wu R, Romano DM, Tek E, O'Malley SS. A preliminary investigation of varenicline for heavy drinking smokers. *Psychopharmacology.* 2011;215(4):655-663.
154. Grant KM, Kelley SS, Smith LM, Agrawal S, Meyer JR, Romberger DJ. Bupropion and nicotine patch as smoking cessation aids in alcoholics. *Alcohol.* 2007;41(5):381-391.
155. O'Malley SS, Krishnan-Sarin S, McKee SA, et al. Dose-dependent reduction of hazardous alcohol use in a placebo-controlled trial of naltrexone for smoking cessation. *The international journal of neuropsychopharmacology.* 2009;12(5):589-597.
156. Anthenelli RM, Heffner JL, Wong E, et al. A Randomized Trial Evaluating Whether Topiramate Aids Smoking Cessation and Prevents Alcohol Relapse in Recovering Alcohol-Dependent Men. *Alcohol Clin Exp Res.* 2017;41(1):197-206.
157. Bold KW, Zweben A, Fucito LM, et al. Longitudinal Findings from a Randomized Clinical Trial of Varenicline for Alcohol Use Disorder with Comorbid Cigarette Smoking. *Alcohol Clin Exp Res.* 2019;43(5):937-944.
158. O'Malley SS, Zweben A, Fucito LM, et al. Effect of Varenicline Combined With Medical Management on Alcohol Use Disorder With Comorbid Cigarette Smoking: A Randomized Clinical Trial. *JAMA psychiatry.* 2018;75(2):129-138.
159. Tindle HA, Freiberg MS, Cheng DM, et al. Effectiveness of Varenicline and Cytisine for Alcohol Use Reduction Among People With HIV and Substance Use: A Randomized Clinical Trial. *JAMA Netw Open.* 2022;5(8):e2225129.
